# Computational Characterization of the Energetics, Structure, and Spectroscopy of Biofuel Precursors: The Case of Furfural‐Acetone‐Furfural

**DOI:** 10.1002/jcc.70297

**Published:** 2025-12-29

**Authors:** Neyla Cherif Benmoussa, Silvia Alessandrini, Laurens de Boer, Sihem Azizi, Hela Friha, Redouane Bachir, Majdi Hochlaf, Cristina Puzzarini, Roberto Linguerri

**Affiliations:** ^1^ Laboratory of Catalysis & Synthesis in Organic Chemistry, University of Tlemcen Tlemcen Algeria; ^2^ Dipartimento di Chimica “Giacomo Ciamician” Università di Bologna Bologna Italy; ^3^ Université Gustave Eiffel, COSYS/IMSE Champs sur Marne France; ^4^ Institut Polytechnique des Sciences Avancées (IPSA) Ivry‐sur‐Seine France

## Abstract

Using density functional theory, we identify the 30 low‐lying structures of the furfural‐acetone‐furfural adduct, a valuable biofuel precursor. Focusing on the most stable species, advanced first‐principles techniques are employed to provide their equilibrium geometries, the full set of their vibrational frequencies, and the pattern of their lowest singlet and triplet electronic states. A high density of isomers/conformers is found even at low energies, which complicates their identification in mixtures. The gas‐phase characterization is complemented by the investigation of the water solvent effects. Our work provides the first accurate structural and spectroscopic characterization of the furfural‐acetone‐furfural adduct and lays the foundation for subsequent studies aiming to optimize their production, to identify them in biofuel reactors, and to investigate their reactivity.

## Graphical abstract

First‐principles computational characterization of the furfural‐acetone‐furfural adduct from structural motif and conformers to vibrational and electronic spectra.

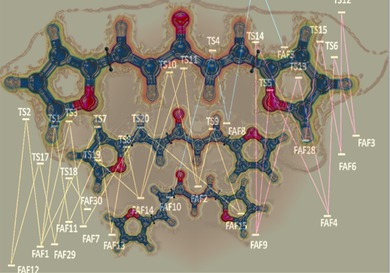

## 1 Introduction

The production of biofuels from biomass feedstocks is a carbon‐neutral and sustainable alternative to the exploitation of fossil resources [1]. Furthermore, it constitutes an effective method of reducing air pollution without necessitating significant alterations to vehicle technology. To this end, second‐generation biofuels can be produced from non‐edible lignocellulosic biomass, such as agricultural and municipal wastes, forestry residues, specialized non‐food crops, and so forth. A well‐known and exploited example of industrial biofuel synthesis from lignocellulosic biomass is the production of bioethanol. The process typically involves pre‐treatment of the raw material, followed by enzymatic hydrolysis and microbial fermentation of the resulting sugar mixtures [2]. In this case, some of the compounds derived from biomass pre‐treatments, such as furfural and 5‐hydroxymethylfurfural, inhibit the fermentation process and their production should be avoided as far as possible [3].

A different approach to second generation biofuels is the production of high‐quality biodiesel, composed mainly of medium and long chain (C_8_–C_21_) alkanes, obtained by catalytic reduction of suitable precursors. These biodiesels are seen as alternatives to fatty acid methyl ester (FAME) ones, which are generally produced by transesterification of vegetable oils and animal fats [4]. Among the most promising precursors for the production of biodiesels and other useful bio‐derived chemicals by catalytic hydrodeoxygenation are furfural‐acetone adducts, obtained by aldol condensation of furfural and acetone, where the furfural moiety can be extracted from the hemicellulose component of the lignocellulosic biomass [5, 6, 7]. Indeed, these platform molecules provide a direct and accessible means of obtaining medium‐ and long‐chain alkanes and alcohols [8]. Furanic compounds such as furfural or 5‐hydroximethylfurfural can be obtained from sugars by catalytic dehydration under mild conditions [9]. While their direct hydrogenation leads to linear C_5_–C_6_ hydrocarbons [10], their aldol condensation produces C_13_–C_15_ adducts, which can be transformed upon hydrogenation and deep hydrodeoxygenation in high‐quality diesel fuels. Several catalytic systems have been proposed for the efficient aldol condensation of furfural and acetone [11, 12, 13, 14, 15, 16, 17, 18, 19, 20].

**FIGURE 1:**
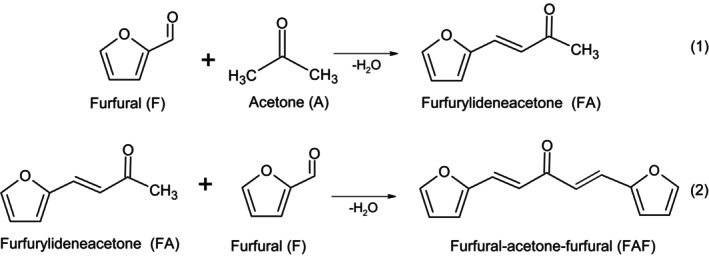
The aldol condensation leading to furfurylideneacetone (FA), followed by a further condensation forming the furfural‐acetone‐furfural (FAF) adduct.

The aldol condensation of furfural (F) and acetone (A) leads to the conjugated enone furfurylideneacetone (FA) as shown in Figure 1. Further condensation of FA with another F unit leads to the furfural‐acetone‐furfural adduct (FAF), known also as difurfurylideneacetone (step 2 in Figure 1). It should be noted that, since furfural has no alpha‐hydrogens, self‐condensation of furfural under conditions favorable for reactions (1) and (2) is not possible. Furthermore, the acetone required for these reactions can itself be produced from renewable biomass, either by fermentation [21] or by decarboxylative ketonization of acetic acid [22]. Starting from the FA or FAF adducts, C_8_ and C_13_ alkanes may be prepared by catalytic hydrodeoxygenation at solvent‐free conditions [23, 24, 25]. More complex and heavier molecules can be synthesized by reduction of other furfural aldol condensation products, such as furfural‐cyclopentanone [26] or furfural‐methyl isobutyl ketone [27, 28].

In order to design optimal catalytic systems for the conversion of FAF precursors into biofuel, it is essential to have a comprehensive understanding of the structural properties of the inherent species. Indeed, an accurate comprehension of the equilibrium configuration of a molecule can provide substantial insights into the reactivity of its different structures, particularly in the context of their interactions with diverse catalysts. Distinct conformers of a reactive molecule, that is, the different spatial arrangements that result from rotation around a single bond, often exhibit distinct chemical behaviors. These differences arise from the influence of the spatial arrangement of atoms on the distribution of electron density and the potential energy surface (PES) of the molecule. Ab initio computations permit chemists to identify the most stable isomers/conformers, determine their relative energies, and predict their interactions with specific catalytic surfaces and/or solvents. This information is vital for the rational design of catalysts, where the objective is to enhance the reactivity or selectivity of a particular conformer, potentially leading to more efficient chemical processes.

An accurate comprehension of the most stable structures is not only fundamental for the study and interpretation of the reactivity, but is also a necessary starting point for the prediction of the relevant parameters for the spectroscopic characterization of FAF in complex biomass mixture. Spectroscopy offers a non‐destructive and highly sensitive approach to the identification and quantification of target molecules, and the calculated parameters provide a reference point for the interpretation of experimental data.

A review of the available literature reveals no comprehensive theoretical studies of the structures and spectroscopic parameters of the stable FAF structures. In light of this, we thoroughly investigated the FAF conformational PES and determined the equilibrium geometries of the lowest‐energy species in the gas phase and solution. Then, to characterize the vibrational (IR) and electronic (UV–VIS) spectra of these species, advanced computational methodologies have been employed to predict their vibrational frequencies and the energies of the transitions from their ground states to the lowest excited electronic states. This comprehensive study paves the way for other investigations aimed at understanding the reactivity of different FAF conformers over catalytic surfaces. This will enable the selection of highly effective catalytic systems, optimizing both yields and catalytic performance.

## 2 Methods

In this section, the methodology employed for the structural and spectroscopic characterization of FAF isomers/conformers is described in detail. Starting from the investigation of the conformational PES, we move to the prediction of the spectroscopic features for the vibrational and electronic structure.

### 2.1 Geometry Optimizations and Force Fields

The conformational PES of FAF has been investigated following a three‐step approach:

- A preliminary study has been performed using the global hybrid B3LYP functional [29, 30] in conjunction with the jun‐cc‐pVDZ basis set [31]. This level of theory, hereafter denoted as B3/junDZ, has been employed to localize minima and connecting transition states in the 0–100 kJ/mol energy range. The nature of each stationary point has been verified by computing the corresponding Hessian at the same level of theory, thus also providing the harmonic zero‐point energy (hZPE) correction to electronic energy.
- To obtain an improved description of the conformers identified in the first step, their equilibrium structures have been re‐investigated using the double‐hybrid rev‐DSDPBEP86 functional [32] in conjunction with the jun‐cc‐pVTZ basis set. In the following, this level of theory is shortly denoted as revDSD/junTZ. While all the minima have been re‐optimized at the revDSD/junTZ level, the harmonic force field has been evaluated only for those lying within a 10 kJ/mol threshold.
- Only for the four most stable species at the revDSD/junTZ level of theory, also including the corresponding harmonic ZPE, cubic and semi‐diagonal quartic force fields have been computed. These have been employed within vibrational perturbation theory to second order (VPT2) to derive relevant anharmonic information, such as anharmonic correction to ZPE, anharmonicity effects on vibrational frequencies and intensity of overtones and combination bands. The level of theory employed for such step is the B3/junDZ because of the large dimension of the systems.

All the calculations described above incorporate the D3(BJ) dispersion correction [33, 34], and have been performed using the Gaussian 16 suite of programs [35].

To account for solvation effects on structures and harmonic frequencies, we resorted to the integral equation formalism variant of the polarizable continuum model (PCM) [36, 37], as implemented in Gaussian 16. The B3/junDZ level of theory and the default settings have been employed for the corresponding calculations. The solvent considered is water (*ε* = 78.3553) and the gas‐phase structures were used as starting points.

### 2.2 Electronic Excited States

Vertical excitation energies for the two lowest singlet (S_0_, S_1_) and two lowest triplet (T_1_, T_2_) electronic states of each stable molecular geometry obtained at the B3/junDZ and revDSD/junTZ levels were calculated using the explicitly correlated internally contracted multi‐reference configuration interaction (MRCI‐F12) method [38, 39, 40] as implemented in MOLPRO 2015 [41]. For these calculations, the reference wavefunctions were constructed using molecular orbitals derived from state‐averaged complete active space self‐consistent field (SA‐CASSCF) computations [42, 43], where equal weights were assigned to the electronic states of each spin multiplicity using the state‐averaging procedure of MOLPRO. These computations were performed in the C_1_ symmetry group. In all cases, the active space in the SA‐CASSCF calculations consists of 8 active electrons in 8 active orbitals (8,8), resulting in 1764 and 2352 configuration state functions (CSFs) for the singlet and triplet electronic states, respectively. The active space was selected as the best compromise between computational cost and accuracy, following tests with active spaces (6,6), (8,8), (10,10), and (12,12). Since, for the most stable isomer, the energy differences between electronic states calculated with the 8‐ and 12‐orbital spaces remain within 0.18 eV at the MRCI‐F12 level, the results obtained with the selected active space (8,8) can thus be considered satisfactorily converged.

In the MRCI‐F12 procedure, the reference wavefunctions were built from the CAS vectors by selecting only those configurations with CI coefficients larger than 0.01. This resulted in a number of uncontracted CSFs of around 4.5 × 10^7^ and 8.3 × 10^7^ for the singlet and triplet states, respectively. The Davidson correction [44] (+Q) was added to the electronic energies. The aforementioned computations were performed with the aug‐cc‐pVDZ basis set [45, 46]. For the many‐electron integral calculations, the cc‐pVDZ/JKFIT and aug‐cc‐pVDZ/MP2FIT basis sets, generated according to MOLPRO's default settings, were employed for the resolution of the identity expansions and the density fitting approximations, respectively. Such a basis set in conjunction with the MRCI‐F12 technique should produce results as accurate as those obtained using the standard MRCI/aug‐cc‐pVQZ level, while greatly reducing the computational cost.

To assess the impact of spin–orbit coupling on the low‐energy states of FAF conformers, spin–orbit matrix elements were evaluated in Cartesian coordinates using the Breit–Pauli Hamiltonian [47]. The CASSCF wave‐functions, generated with an active space of 8 electrons in 8 molecular orbitals and state‐averaged over two singlet and two triplet states with equal weights, served as the multielectron basis for the two‐step spin–orbit coupling calculations. The spin–orbit‐coupled states were obtained by diagonalizing the electronic spin–orbit Hamiltonian.

## 3 Results

The results obtained in this work are presented and discussed following the outline introduced in the methodology section. We start from the structural characterization and then we move to spectroscopy following an increasing frequency order.

### 3.1 Conformational Analysis

**FIGURE 2:**
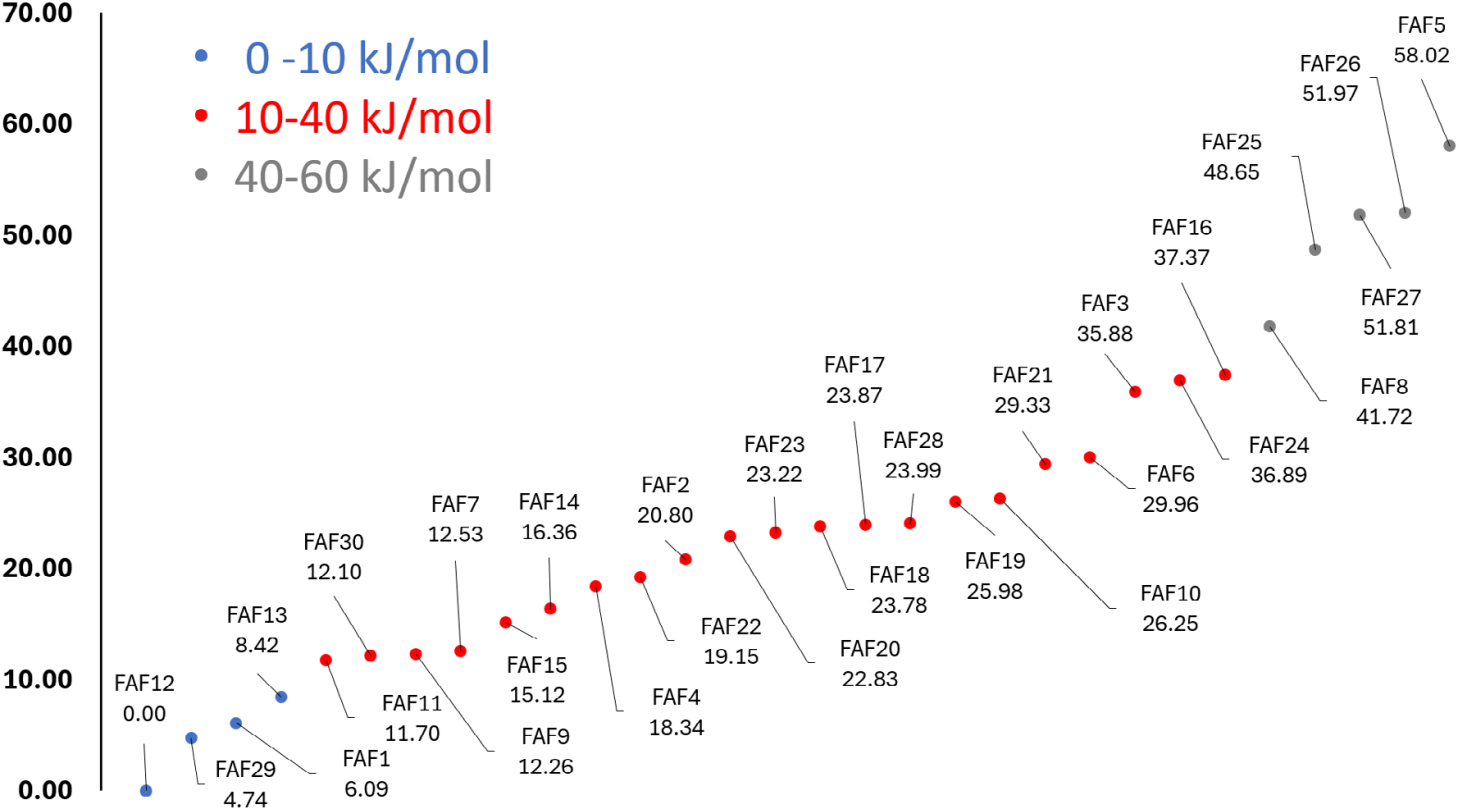
Relative energies of the thirty FAF species considered in this work as obtained from the revDSD/junTZ level of theory corrected for the harmonic ZPE at the B3/junDZ level.

The investigation of the conformational PES at the B3/junDZ level led to the identification of 30 structures lying in an energy interval of about 60 kJ/mol. About ten of these species have been generated using CREST with the GFN‐FF method [48, 49], while additional species were obtained as a result of intrinsic reaction coordinate (IRC) analysis [50]. In this case, the new minimum was re‐optimized, and the nature of the stationary point was verified by computing the corresponding Hessian matrix. The graphical representation of all the species identified together with their cartesian coordinates is provided in the Figure S1 of the Supporting Information (SI). The stability order, based on the revDSD/junTZ energy corrected for the hZPE at the B3/junDZ level, is graphically shown in Figure 2. The numbering of the conformers is due to the chronological order of their identification and not related to the energy order. In Table 1, the energetics at the B3/junDZ and revDSD/junTZ levels, with and without hZPE correction, are compared for the nine FAF structures that result to be the most stable at the revDSD/junTZ level (with B3/junDZ hZPE correction incorporated). For helping the reader, a graphical representation of their structure is also reported.

**TABLE 1:** Relative electronic (ΔE) and free (ΔG; at 298 K) energies and their harmonic ZPE (hZPE)‐corrected counterparts (kJ/mol) for the nine most stable FAF species at the B3/junDZ and revDSD/junTZ levels. For the four most stable structures, the anharmonic contribution to harmonic ZPE (anhΔZPE, B3/junDZ) has also been considered.

Structure		B3/junDZ	B3/junDZ + ZPE: B3/junDZ	revDSD/junTZ	revDSD/junTZ + hZPE: B3/junDZ	ΔG(298) revDSD/junTZ + thc: B3/junDZ^a^	revDSD/junTZ + hZPE: revDSD/junTZ	revDSD/junTZ + hZPE revDSD/junTZ + anhΔZPE B3/junDZ^b^	ΔG(298 K) revDSD/junTZ + thc: revDSD/junTZ^a^
FAF12	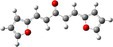	0.00	0.00	0.00	0.00	0.00	0.00	0.00	0.00
FAF29	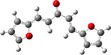	5.87	6.05	4.56	4.74	2.37	4.61	4.49	1.80
FAF1	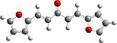	5.93	5.37	6.65	6.09	3.83	6.36	6.60	5.34
FAF13	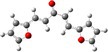	9.01	8.72	8.70	8.42	5.63	8.22	8.37	4.97
FAF9	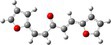	7.66	8.73	11.18	12.26	11.70	—	—	—
FAF7	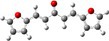	12.18	11.11	13.60	12.53	11.54	—		—
FAF11	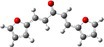	12.84	12.38	12.16	11.70	8.56	—		—
FAF15	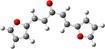	15.54	14.72	15.94	15.12	11.67	—		—
FAF30	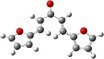	16.17	15.96	12.32	12.10	10.49	—		—

^a^ thc stands for thermal correction (entropic contribution).

^b^ hZPE revDSD/junTZ + anhΔZPE B3/junDZ means harmonic ZPE correction at the revDSD/junTZ level augmented by the anharmonic contribution to ZPE at the B3/junDZ level.

From the inspection of this table, it is noted that, at both levels of theory, the FAF12 structure is the most stable one, while FAF29 is the second most stable with the only exception of B3/junDZ when the corresponding hZPE correction is incorporated. Indeed, at the B3/junDZ level, FAF29 and FAF1 energies differ by only 0.06 kJ/mol but hZPE correction increases the relative energy of FAF29 while it decreases that of FAF1. Differently, at the revDSD/junTZ, FAF29 is more stable than FAF1 by about 2.1 kJ/mol, the difference decreasing to ~1.3 kJ/mol once the B3/junDZ hZPE correction is added. Moving higher in energy, we find FAF13 and FAF9, which are separated by about 0.01 kJ/mol at the B3/junDZ level with the corresponding hZPE correction incorporated and by ~3.8 kJ/mol when the revDSD/junTZ energy is considered together with the B3/junTZ hZPE contribution. The next conformers are FAF7 and FAF11 whose stability is reversed at the B3/junDZ and revDSD/junTZ levels. The last two conformers considered in Table 1 are, in ascending order, FAF15 and FAF30, with the stability order reversing when moving from B3/junDZ to revDSD/junTZ (both corrected for B3/junDZ hZPE). The hZPE contribution at the revDSD/junTZ level does not alter the energy order for the species lying within 10 kJ/mol, thus confirming FAF12 as more stable than FAF29 and FAF1. Only for the four most stable species of Table 1, the anharmonic contribution to the ZPE (anhΔZPE) has been computed at the B3/junDZ level. The incorporation of this anharmonic correction does not affect the energy order, but reduces the energy difference between FAF12 and FAF29 to only 2.39 kJ/mol. With the aim of supporting future comparison with experiment, Table 1 also reports the relative free energy computed at 298 K, ΔG (298 K), at two levels of theory. The first is based on entropic correction computed from the harmonic B3/junDZ force field and it is provided for all species. No major change is observed in the stability order, with only FAF11 becoming about 3 kJ/mol more stable than FAF9 and FAF7. Instead, for the four stable species, ΔG (298 K) incorporating the thermal correction (thc) at the revDSD/junTZ energy level is also provided. This leads to an inversion of the stability order of FAF1 and FAF13. Furthermore, they become closer in energy, being all located within 5 kJ/mol. Before moving on, the accuracy of the energetics reported in Table 1 deserves to be addressed. According to the literature (see, e.g., refs. [32, 51, 52]), the revDSD/junTZ level of theory is expected to provide relative energies with an accuracy of a few kJ/mol whenever the system, as in the present case, is well described by a single‐reference wave function. The accuracy of revDSD/junTZ energies is not significantly affected by the incorporation of hZPE corrections at the B3/junDZ level due to their small contribution, but especially because ZPE differences enter the evaluation of relative energies. Finally, using harmonic approximation to calculate ZPE corrections has an even lower impact on accuracy because hZPE accounts for about 95%–98% of its anharmonic counterpart [53, 54]. Indeed, for the non‐revised DSDPBEP86 functional in conjunction with a partially augmented basis set, the ZPE scaling factor is about 0.982–0.983 [55, 56]. This is further confirmed by the results reported in Table 1 for the four most stable species.

**FIGURE 3:**
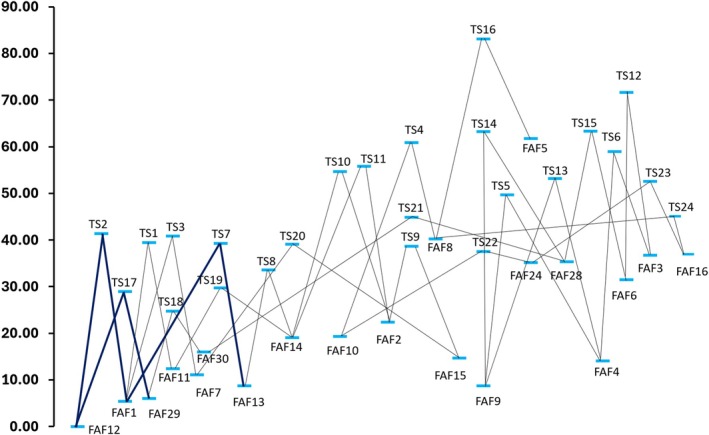
Conformational PES at the B3/junDZ level, also incorporating harmonic ZPE B3/junDZ corrections. Energies in kJ/mol. In dark blue the interconversion path of the low‐energy conformers.

In Figure 3, the conformational PES also showing the transition states connecting the minima is reported. The corresponding list of energies is provided in Tables S1 and S2 of the SI. From these tables and Figure 3, it is apparent that the conformational barriers vary from a minimum of ~10 kJ/mol (from FAF30 to FAF29) to a maximum of ~50 kJ/mol (from FAF9 to FAF28), with most of them lying in the 30–50 kJ/mol range. Therefore, the overall conclusion is that these barriers are sufficiently high to avoid interconversion of the system from one conformer to the other at room temperature (this latter implying a thermal energy of about 2.5 kJ/mol). From Figure 3 it is also evident that the second and third most stable forms (FAF29 and FAF1) are directly connected to the most stable one (FAF12) through TS17 for the interconversion FAF29‐FAF12 and through TS2 for that between FAF1 and FAF12. The barrier ruled by TS17 is about 29 kJ/mol, while that ruled by TS2 is about 36 kJ/mol. The fourth most stable species, FAF13, is only connected to FAF1, via TS7 located at 27 kJ/mol above FAF1. It has to be noted that, on the PES, no direct rotations along the C=C bond connecting the furan ring to the C=O moiety (which leads to isomerization) have been found. Therefore, rotations of the ethylenic‐H atoms in a trans to cis conformation more than likely require higher energies than those scanned, and the corresponding TSs are probably located above the 100 kJ/mol threshold considered. To give an example, the direct C=C rotation from FAF1 to FAF9 was not observed on the PES. All the FAF conformers are characterized by the delocalization of the π electrons over all the molecule from one furanic ring to the other. The resulting structures derive from a balance between electron delocalization, intramolecular non‐covalent interactions and steric effects.

**FIGURE 4:**
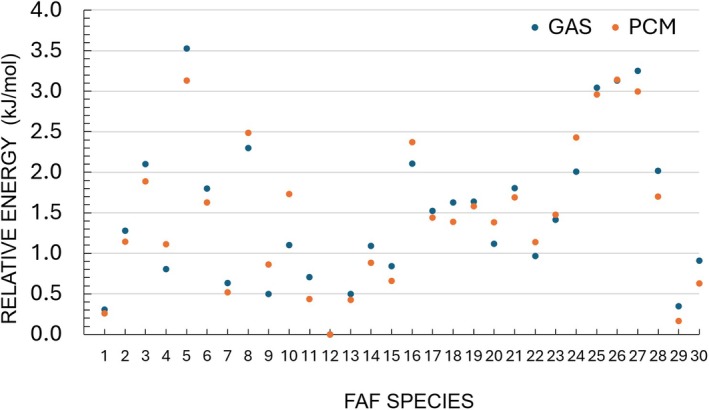
B3/junDZ relative energies of the FAF species incorporating hZPE at the same level of theory: Comparison between gas‐phase and PCM results.

In Figure 4, the comparison between the gas‐phase energetics and that in solution obtained by means of the PCM approach is graphically provided. From the inspection of this figure, it is noted that the variation of relative energy moving from the gas phase to solution is quite small, this being on average 0.2 kJ/mol. The maximum difference observed is 0.63 kJ/mol for FAF10. This might be related to the peculiar structure of this conformer (see Figure S1), which is planar with a cis‐cis orientation of the two furanic rings (with the O atoms facing the outer part of the molecule) forming two hydrogen bonds with the carbonyl oxygen.

### 3.2 Molecular Structure

To the best of our knowledge, for FAF, there is a complete lack of studies on its molecular structure and conformational analysis in the literature. In ref. [15], the catalytic aqueous‐phase aldol‐condensation of acetone and furfural catalyzed by three different mixed‐oxides was investigated. To gain insights into the reaction mechanism, the authors performed ^1^H‐NMR spectroscopic analysis of the reaction products. On the base of the spin–spin J‐coupling constants, such analysis led them to the identification of three different structures defined by the authors as trans‐trans, cis‐trans and cis‐cis isomers, where cis and trans refer to the position of the furanic‐oxygen with respect to carbonyl group. According to the study of ref. [15], the cis‐cis form is first formed in high concentration, while after some time (about 2 h) the trans‐trans species becomes dominant. The cis‐trans FAF was found to be an unstable form (with a constant concentration of ~5%) and was considered as an intermediate in the isomerization reaction. Unfortunately, the authors of ref. [15] did not provide detailed information on the geometrical parameters, but only their graphical representation. We can probably assume that the structures reported in ref. [15] are mainly qualitative pictures of the FAF systems. From a comparison of the structures reported on figure 5 in ref. [15] with ours, the cis‐cis form resembles our FAF5, which is the one lying highest in the energy range we considered, the trans‐trans species seems to be our FAF1, which is the third conformer in the stability order, and the cis‐trans isomer might be our FAF8, which is the fourth least stable form. Indeed, FAF8 and FAF5 are connected through the transition state TS16. Differently, we did not find any direct connection between either FAF5 or FAF8 and FAF1 (see Figure 3).

In ref. [57], Agrawal and coworkers investigated the conversion of xylose to linear alkanes in aqueous phase and FAF is among the products considered. More precisely, they evaluated the thermochemical properties associated to all the reaction steps. The structures of all the compounds involved in the study were optimized in the gas phase at the B3LYP/6‐31+G(d,p) and M06‐2X/6‐31+G(d,p) levels and then re‐optimized in the aqueous‐phase using the SMD solvation model (see ref. [57] for details). Unfortunately, the authors did not report any geometrical parameters at any level. The only structural information is a graphical (and thus qualitative) representation of the optimized geometries in the aqueous phase for both the levels of theory considered. It is noted that the only conformer considered by the authors in ref. [57] corresponds to our FAF7, which is only the 8th most stable conformer. Therefore, it can be supposed that the corresponding thermochemical properties are affected by a systematic bias.

The consideration of water solvent, via PCM model, leads to optimized structures that are quite similar to those found in gas phase. In Figure S2, the graphical representation of the structural differences is provided, while Table S3 collects the absolute mean difference, for each FAF species, between the gas‐phase and PCM‐solvated cartesian coordinates. It is noted that the values are small (few hundredths of Å).

### 3.3 Infrared Spectrum

**FIGURE 5:**
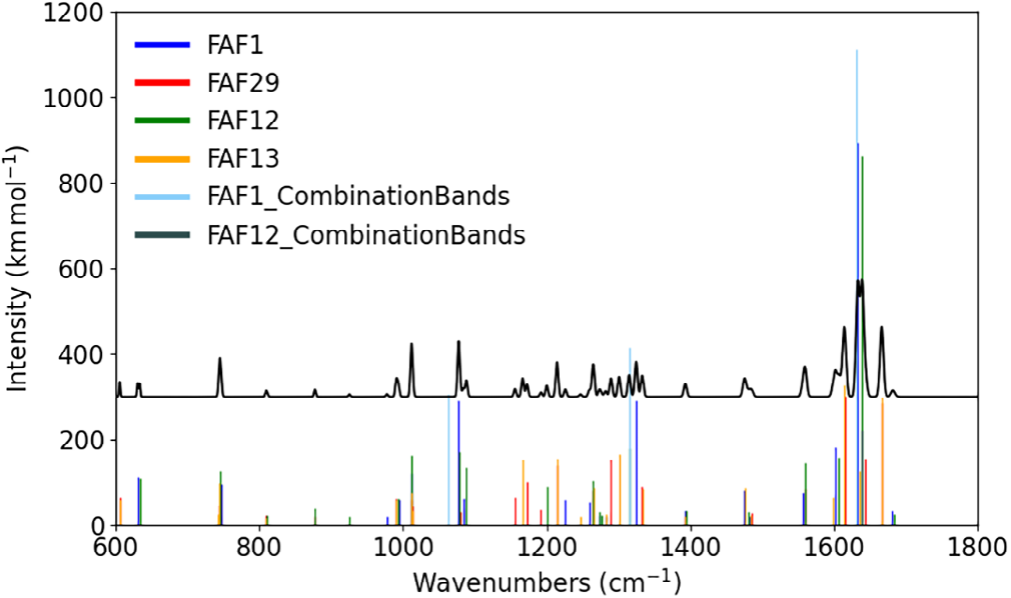
Simulation of the IR spectra of the four most stable conformers of FAF (FAF12, FAF29, FAF1, FAF13) at the revDSD/junTZ level. Only transitions with intensities greater than 20 km/mol are reported for fundamentals, while for combination a threshold of 200 km/mol was applied. The black trace (offset for clarity) plots the IR spectrum resulting from the inclusion of Boltzmann's factor, which is then convoluted using Gaussian functions with a HWHM of 4 cm^−1^.

The scaled harmonic wavenumbers and the corresponding IR intensities at the revDSD/junTZ level for the four most stable FAF conformers (i.e., those lying within the 10 kJ/mol range) are collected in the SI (Tables S4–S7). For FAF12, FAF29, FAF1 and FAF13, the stick IR spectrum within the double‐harmonic approximation is graphically represented in Figure 5, where the Boltzmann population is not accounted for but instead all the four species are considered equally populated. For all of them, it is noted that the IR spectrum is very weak above 1800 cm^−1^ and below 600 cm^−1^, with the most intense bands lying in the 1000–1100, 1250–1350, and 1600–1700 cm^−1^ regions. Going into details, FAF12 and FAF1 show similar spectra. For them, the two most (very) intense bands lie close in frequency: 1681 cm^−1^ for FAF12 and 1675 cm^−1^ for FAF1. The vibrational mode is in both cases a concerted stretching of the two CC double bonds (left and right with respect to the C=O group). The other rather intense features are in the 1000–1100 cm^−1^ range and around 1350 cm^−1^; in all these cases, the vibrations involved are CCH in‐plane rocking modes. Similarly, the IR spectrum of FAF29 is paired with that of FAF13. The 1650–1700 cm^−1^ region is dominated by three bands, which are however by far less intense than those discussed above for FAF12 and FAF1 (around 1680 cm^−1^). Such a difference is mainly related to the lack of symmetry in the C=C—C(O)—C=C moiety.

The C=O stretching is the most prominent feature for FAF29 and FAF13 IR spectra. Indeed, at 1656 cm^−1^ (FAF29) and 1654 cm^−1^ (FAF13), we find the C=O stretching with an intensity which is double than that of the corresponding bands of FAF12 and FAF1, the frequencies being instead very similar (1648 cm^−1^ for FAF12 and 1643 cm^−1^ for FAF1). A bit less intense are the other two bands lying at 1686/1679 cm^−1^ (FAF29/FAF13) and 1709 cm^−1^. The former is the C=C stretching of the ethylene group that, based on the geometries shown in Table 1, is on the right side of carbonyl moiety. The feature at 1709 cm^−1^ is instead a concerted C=O and C=C stretching vibration, involving the other ethylene moiety (left side). For FAF29 and FAF13, five bands, with intensities ranging between ~90 and 170 km/mol, lie between 1190 and 1370 cm^−1^. In analogy to FAF1 and FAF12, the corresponding vibrations are CCH in‐plane rocking modes.

For quantitative predictions of vibrational spectra, in addition to an accurate level of theory (noted is that revDSD/junTZ performs similarly to CCSD(T)), anharmonicity effects need to be taken into account. However, for large systems as those under consideration in this work, these require expensive anharmonic force‐field calculations. A possible way‐out is offered by scaling factors. In ref. [55], Martin and coworkers evaluated those for the DSDPBEP86 functional in combination with different basis sets and obtained values ranging from 0.9972 and 0.9993 when triple‐zeta basis sets were considered. In ref. [32], in addition to provide different revisions of the DSDPBEP86 functional, Martin and coworkers used “harmonic frequencies as a sanity check”. From the inspection of Figure 3 of ref. [32], we can note that revDSD‐PBEP86‐D3(BJ) in conjunction with the def‐QZVP basis set shows a slightly improved performance, with the derived scaling factors for harmonic frequencies negligibly differing from 1.0. However, a partially augmented triple‐zeta basis set has been employed in this work. VPT2 calculations performed on top of the B3/junDZ anharmonic force field allowed us to explicitly investigate the effect of anharmonicity. This decreases the transition frequencies by about 2.5% in a rather systematic manner for the vibration above 200 cm^−1^. Therefore, a scaling factor of 0.975 is suggested.

Furthermore, anharmonic B3/junDZ calculations allowed us to consider overtones and combination bands, whose intensities are not accessible within the double‐harmonic approximation. The results for the most intense bands are reported in Tables S8–S11 of the SI. FAF12 has a strong combination mode predicted at 1638.5 cm^−1^. Similarly, FAF1 has a strong combination band at 1631.5 cm^−1^ that is predicted to be about five times more intense than the one of FAF12. FAF1 has other seven intense combination bands in the region 1050–1670 cm^−1^, which might be used to recognize this species in the experimental spectrum. For this reason, they have been added to Figure 5. Instead, FAF13 and FAF29 have several combination bands of medium intensity, but no clear features able to unequivocally assign these conformers within a mixture.

In Figure 5, the IR spectrum resulting from the convolution of the stick spectra with a Gaussian function with the half‐width at half maximum (HWHM) of 4 cm^−1^ and accounting for the Boltzmann distribution is also depicted. From its inspection, it is apparent that each of the four FAF species considered shows at least a feature, either intense or rather weak, able to unequivocally demonstrate its presence in the gas mixture.

Table S12 provides the harmonic frequencies, at the B3/junDZ level, for all the 30 FAF species computed both in the gas phase and in solution using the PCM model. The table also reports the differences, thus allowing an easier inspection of the solvation effects. It is noted that the deviations are small, the mean unsigned deviation being 4 cm^−1^. The only relevant differences are observed for some C=O stretching modes, whose frequencies change by a few tens of wavenumbers, as already noticed for carbonyl compounds [58, 59, 60].

### 3.4 Electronic Excited States and UV Spectrum

In Table 2, we provide the vertical excitation energies, calculated at the MRCI‐F12+Q/aug‐cc‐pVDZ level, from the ground to the lowest excited electronic states of the nine most stable FAF structures, that is, those lying within ~15 kJ/mol (~0.155 eV) threshold when considering the hZPE‐corrected revDSD/junTZ energetics (see Figure 2). In particular, excitations to the lowest singlet (S_1_) and two lowest triplet (T_1_, T_2_) states are considered. Calculations of vertical transitions involving higher levels of excitation could not be performed in the present study because of the excessive dimension of the required active spaces. In Table 2, the excitation energies are computed on top of both the B3/junDZ and revDSD/junTZ optimized geometries. The results for the complete set of structures (at the B3/junDZ(geom)//MRCI‐F12+Q/aug‐cc‐pVDZ level) are given in Table S13 of the SI. As shown in Table S14, the ground states (S_0_) of most conformers considered here can be accurately described by a single electronic configuration, although notable exceptions exist (e.g., FAF5). In contrast, while the first excited singlet states (S_1_) are predominantly characterized by single configurations, several conformers (e.g., FAF5, FAF14, FAF16, FAF18, FAF25, and FAF27) exhibit significant multiconfigurational character in this state. This multiconfigurational nature becomes particularly pronounced in the triplet states. Consequently, the electronic structures of these low‐energy states are inherently challenging to model, limiting the applicability of single‐reference methods such as TD‐DFT.

**TABLE 2:** Vertical excitation energies^a^ (in eV) of the lowest electronic states (S1, T1, and T2) for the most stable FAF conformers at the revDSD/junTZ(geom)//MRCI‐F12+Q/aug‐cc‐pVDZ level. In parentheses, the B3/junDZ(geom)//MRCI‐F12+Q/aug‐cc‐pVDZ results are reported.

	S_0_	S_1_	T_1_	T_2_
FAF12	0	4.44 (4.38)	3.27 (3.20)	3.28 (3.21)
FAF29^b^	0	4.87 (4.88)	3.91 (3.92)	4.05 (4.05)
FAF1	0	4.46 (4.41)	2.69 (2.61)	5.24 (5.18)
FAF13	0	4.56 (4.57)	3.28 (3.29)	3.51 (3.52)
FAF11	0	4.53 (4.54)	3.33 (3.32)	3.42 (3.42)
FAF30	0	5.55 (5.52)	3.03 (2.98)	3.08 (3.15)
FAF9	0	4.25 (4.18)	2.73 (2.65)	5.09 (5.04)
FAF7	0	4.46 (4.40)	2.68 (2.61)	5.23 (5.17)
FAF15	0	4.45 (4.39)	3.33 (3.26)	3.46 (3.39)

^a^ Relative energy given with respect to the S_0_ energy.

^b^ For FAF29, values are computed at the CASSCF/aug‐cc‐pVDZ level of theory.

To better understand the nature of the low‐lying excited states of the FAF conformers, we examined the frontier orbitals of all 30 FAF conformers. These orbitals, obtained at the CASSCF/aug‐cc‐pVDZ level with 8 electrons in 8 orbitals optimized for the lowest singlet state, are depicted in Figure S3 of the SI. As illustrated in this figure, the frontier orbitals are either localized on a small group of atoms—either at the center of the molecular framework or along the furfural rings—or completely delocalized. They exhibit, as expected, π‐character, originating primarily from combinations of p orbitals of heavier atoms. Although there is no systematic correlation between orbital localization or delocalization and the multireference character of the wavefunction, it can be observed that, for the triplet states T_1_ and T_2_, the wavefunction is predominantly mono‐configurational when the frontier orbitals are localized at the center of the molecular framework. Indeed, delocalized orbitals that extend over multiple atoms in conjugated systems tend to exhibit smaller orbital energy gaps, meaning that the orbitals are energetically closer and can mix more readily. This can result in significant contributions from multiple electronic configurations, thereby increasing the multiconfigurational character of the wavefunction.

Finally, Figure S4, which displays the outermost molecular orbitals of the most stable conformer (FAF12), shows that the selected active space (8,8) includes the relevant oxygen lone pair orbitals and the π, π* orbitals of FAF, thus ensuring a good description of the *n*—π and π—π* electronic transitions.

It is worth noting that we encountered significant challenges in converging the MRCI‐F12 calculations of the patterns of the electronic states of the FAF structures, in particular for FAF29. Indeed, the calculations for FAF29 could not be converged even by significantly increasing the active space. The difficulty in converging these MRCI‐F12 computations is due to the complex nature of the electronic states of the FAF molecule, particularly for this species, as well documented in the literature for furan, substituted furan and FA compounds [61, 62, 63, 64, 65, 66]. This complicates the description of the electronic structure at the chosen level of theory. Some refined approaches, incorporating more appropriate (diffuse) one‐electron atomic sets, active spaces beyond those based on valence molecular orbitals [67, 68, 69], and better treatment of electron correlation may be required to achieve more accurate results for FAF29. However, this would be computationally intensive and particularly challenging for a molecule of this size. Therefore, in Table 2, for FAF29, we present the results obtained using the CASSCF method rather than those derived from MRCI‐F12+Q.

Table 2 shows that the excitation energies calculated at the MRCI‐F12+Q/aug‐cc‐pVDZ level using either the B3/junDZ or revDSD/junTZ geometries are very similar to each other, with an average difference of 0.04 eV, well below the typical experimental error bars for such quantities. In view of such a good agreement and since the hybrid approach using the revDSD/junTZ geometry is more computationally demanding, we recommend performing electronic excitation calculations at either the CASSCF or MRCI‐F12 level on top of the cheaper but still accurate enough B3/junDZ optimized geometries for large molecular systems such those under consideration in this work.

Table 2 shows that the calculated excitation energies lie between 4.25 and 5.55 eV for the S_1_ ← S_0_ transition and within 2.68 and 5.24 eV for the T_1_ ← S_0_ and T_2_ ← S_0_ ones. For the four most stable conformers FAF12, FAF29, FAF1, and FAF13, that is, those in an energy window of 0.1 eV (10 kJ/mol) above the most stable conformer, the spin‐allowed S_1_ ← S_0_ transition averages 4.58 eV (271 nm), while for the spin‐forbidden T_1_ ← S_0_ we compute an average value of 3.29 eV (377 nm). These transitions lie in the UV and visible regions of the electromagnetic spectrum, respectively. For these four species, the T_2_ ← S_0_ transitions have instead a greater variability, ranging from about 3.3–5.2 eV. For the S_1_ ← S_0_ absorption, the computed transition dipole moments are calculated as 9.83, 8.31, 7.27, and 7.86 debye for FAF12, FAF29, FAF1 and FAF13, in that order. These values are quite large, i.e., well above 5 debye, and correspond to very intense electronic transitions, easily detectable in the UV–VIS spectra of these species. Other structures show similar values of the transition dipole moment for the same transition.

Singlet to triplet transitions are expected to be weak because of their spin‐forbidden nature. In principle, they could gain intensity through spin‐orbit coupling and play a role in experiments in which phosphorescence is measured after excitation of FAF by UV or visible light. However, for all FAF species examined at their equilibrium geometries, the computed energy shift between spin–orbit and non–spin–orbit states, as described in the Computational Details, is found to be negligible. This result aligns with the expected behavior of molecules consisting only of first‐ and second‐row elements.

## 4 Conclusions

In the present work, a thorough investigation of the conformational PES of FAF has been carried out. After a preliminary study at the B3/junDZ level, the structural and energetic characterization has been improved by exploiting the revDSD/junTZ level of theory. Among the 30 structures identified in a 60 kJ/mol energy interval, the four most stable conformers (i.e., those lying within the 10 kJ/mol range above the most stable form) have been considered for a deeper study. For them, the harmonic force field has also been evaluated at the revDSD/junTZ level, thus allowing the accurate prediction of the IR spectra within the double‐harmonic approximation. To go beyond such approximation, anharmonic force‐field calculations have also been performed at the B3/junDZ level. Concerning the equilibrium geometries, according to the literature on this topic (see, e.g., ref. [65]), the uncertainties of revDSD/junTZ are ~4 mÅ for bond lengths and 0.15 deg. for angles. The accuracy of the revDSD/junTZ harmonic frequencies, once corrected for accounting for anharmonicity, is expected to be ~5 cm^−1^ in terms of mean absolute error [70, 71], while the corresponding intensities should be accurate to about 2 km/mol. Moreover, our PCM calculations show that solvent effects only slightly affect the equilibrium geometries of FAF species. The same applies to most of the vibrational frequencies, the only exceptions being some appreciable frequency shifts in the C=O stretching modes.

For all the calculated structures, the UV–VIS spectra show absorptions in the UV region at around 270 nm, due to the S_1_ ← S_0_ transition, while the spin‐forbidden T_1_ ← S_0_ transition is expected at the limit of the visible spectrum, that is, at around 380 nm. From a methodological point of view, we showed that the less computationally demanding B3/junDZ(geom)//MRCI‐F12+Q/aug‐cc‐pVDZ hybrid approach is accurate enough to compute the pattern of the lowest electronic states of FAF compounds and more generally can be extended to calculate those of larger molecular systems. Spin‐orbit coupling effects have been found entirely negligible.

The spin allowed transitions of lowest energy are predicted to be very intense, as confirmed by the computed values of the transition dipole moment, and can be used for the detection of FAF conformers in complex mixtures of biomass derivatives. Although the low‐energy species in Table 2 display similar intensities of transition, subtle differences in their absorption and emission spectra (peak positions, shape, bandwidth) can result from variations in the electronic environment around the furfural units. These differences, although small, can be probed using advanced spectroscopic techniques, making it possible to identify the molecule of interest in a mixture of similar species.

Our results show that ensemble‐averaged UV–VIS and IR spectra of FAF formed under process‐relevant (bioreactor) conditions are expected to be intrinsically congested owing to the large number of accessible conformers. By contrast, in situ spectra acquired under the catalytic conditions that generate FAF are often comparatively simple, consistent with a restricted subset of conformers contributing measurably to the signal [62, 63, 65]. In fact, the conformers populated during catalysis need not coincide with the thermodynamic minima predicted for isolated molecules: kinetic control, catalyst‐substrate interactions, and the reaction environment can bias populations toward higher‐energy yet long‐lived structures. As noted in the Introduction, experiments aimed at producing biofuel precursors are not only difficult to perform but also challenging to interpret, due to the expected, yet often unobserved, contributions of multiple low‐lying conformers.

## Author Contributions

Conceptualization: M.H., R.L., S.Al. Data curation: S.Al., L.d.B., C.P., R.L. Formal analysis: S.Al., R.L., C.P. Funding acquisition: C.P., M.H., R.L. Investigation: N.C.B., R.B., H.F., S.Az., L.d.B. Methodology: S.Al., M.H., R.L., C.P. Project administration: C.P., S.Al., R.L. Software: S.Al. and R.B. Resources: C.P. and M.H. Supervision: R.L. and M.H. Validation: S.Al. and C. P. Visualization: S.Az. and R.B. Writing – original draft: C.P., R.L., S.Al. Writing – review and editing: S.Al., M.H., R.L., C.P.

## Funding

This work was supported by Ministero dell'Università e della Ricerca [202082CE3T, P2022ZFNBL, 20225228K5]; University of Bologna (RFO funds).

## Conflicts of Interest

The authors declare no conflicts of interest.

## Supporting information


**Data S1:** jcc70297‐sup‐0001‐DataS1.pdf.

**Data S2:** jcc70297‐sup‐0002‐DataS2.zip.

## Data Availability

The data supporting this article have been included as part of the Supporting Information.
