# Supplementary material for: Computational Characterization of the Energetics, Structure, and Spectroscopy of Biofuel Precursors: The Case of Furfural‐Acetone‐Furfural
Source: J Comput Chem. 2025 Dec 29;47(1):e70297. doi: 10.1002/jcc.70297 (PMC12747218; doi:10.1002/jcc.70297)
Supplement: Supplementary file 1 — Data S1: jcc70297‐sup‐0001‐DataS1.pdf. [file JCC-47-0-s002.pdf]

SUPPORTING INFORMATION

Computational characterization of the energetics, structure and spectroscopy of biofuel precursors: The case of furfural-acetone-furfural

Neyla Cherif Benmoussa,[1] Silvia Alessandrini,[2,*] Laurens de Boer,[3] Sihem Azizi,[1] Hela Friha,[3,4] Redouane Bachir,[1] Majdi Hochlaf,[3] Cristina Puzzarini,[2] Roberto Linguerri [3,*]

[1] Laboratory of Catalysis & Synthesis in Organic Chemistry, University of Tlemcen, Algeria.

[2] Dipartimento di Chimica "Giacomo Ciamician", Università di Bologna, Via P. Gobetti 85, 40129 Bologna, Italy.

[3] Université Gustave Eiffel, COSYS/IMSE, 5 Bd Descartes 77454, Champs sur Marne, France.

[4] Institut Polytechnique des Sciences Avancées (IPSA), 63 Bd de Brandebourg Bis, 94200, Ivry-sur-Seine, France.

---

* Authors for correspondence:

S. Alessandrini: e-mail: silvia.alessandrini7@unibo.it

R. Linguerri: e-mail: roberto.linguerri@univ-eiffel.fr

Contents

1. Technical computational details 3
2. Furfural-Acetone-Furfural (FAF) species 3
   2.1. Energies 3
   2.2. Structures and Cartesian Coordinates 5
      2.2.1. Graphical representation of the equilibrium geometries of the identified FAF structures 5
      2.2.2. Cartesian Coordinates of the identified FAF species at the B3/junDZ level of theory. 6
3. Transitions States for the intercoversion between FAF species 14
   3.1. Energies 14
4. Comparison of the gas-phase and PCM structures 15
5. Infrared Spectrum: 17
   5.1. Fundamental bands 17
   5.2. Combinations bands and Overtones 21
6. Vertical excitation energies for the FAF species 50
7. Configurations and Frontiers orbital for the thirty FAF species at the CASSCF(8,8)/aug-cc-pVDZ level.. 51

1. Technical computational details

All the quantum-chemical calculations for the electronic ground state have been performed using the Gaussian 16 suite of programs.

Gas phase. In all calculations the SCF convergence was set to 10^{-8} for both energy and density. For geometry optimizations, the "verytight" criterium was employed (which means at least three of the following thresholds must be fulfilled: Max Force: 0.000015, RMS Force: 0.000010, Max Displacement: 0.00006, RMS Displacement: 0.00004). For transition state optimization, the criterium was set to "tight" (the corresponding thresholds are about thirty-times larger than "verytight" option). For numerical quadrature grids, the "UltraFine" grid was employed, i.e. the default for Gaussian16, with 99 radial points for 590 angular points per atom.

Solution. For the PCM calculations, the default settings have been employed.

2. Furfural-Acetone-Furfural (FAF) species

2.1. Energies

Table S1. Absolute and relative energies for the FAF conformers/isomers at different level of theory.

	ABSOLUTE ENERGIES (Hartree)				RELATIVE ENERGIES (kJ/mol)		
	B3/junDZ	revDSD/junTZ	ZPE(B3/junDZ)	ZPE(revDSD/junTZ)	B3/junDZ+ ZPE(B3/junDZ)	revDSD/junTZ+ ZPE(B3/junDZ)	revDSD/junTZ+ ZPE(revDSD/junTZ)
**FAF12**	-727.12121	-725.94130	0.19698	0.19764	0.00	0.00	0.00
**FAF29**	-727.11897	-725.93956	0.19705	0.19765	6.05	4.74	4.61
**FAF1**	-727.11895	-725.93877	0.19677	0.19753	5.37	6.09	6.36
**FAF13**	-727.11778	-725.93799	0.19687	0.19745	8.72	8.42	8.22
**FAF11**	-727.11631	-725.93667	0.19681		12.38	11.70	11.51
**FAF30**	-727.11505	-725.93661	0.19690		15.96	12.10	
**FAF9**	-727.11829	-725.93704	0.19739		8.73	12.26	

**FAF7**	-727.11657	-725.93612	0.19657	11.11	12.53
**FAF15**	-727.11529	-725.93523	0.19667	14.72	15.12
**FAF14**	-727.11370	-725.93483	0.19674	19.09	16.36
**FAF4**	-727.11604	-725.93451	0.19718	14.07	18.34
**FAF22**	-727.11519	-725.93442	0.19740	16.90	19.15
**FAF2**	-727.11228	-725.93298	0.19658	22.41	20.80
**FAF20**	-727.11399	-725.93284	0.19721	19.55	22.83
**FAF23**	-727.11179	-725.93247	0.19699	24.75	23.22
**FAF18**	-727.11006	-725.93194	0.19668	28.47	23.78
**FAF17**	-727.11094	-725.93212	0.19689	26.72	23.87
**FAF28**	-727.10751	-725.93194	0.19676	35.38	23.99
**FAF19**	-727.10998	-725.93112	0.19669	28.70	25.98
**FAF10**	-727.11469	-725.93214	0.19782	19.31	26.25
**FAF21**	-727.10890	-725.92987	0.19672	31.61	29.33
**FAF6**	-727.10929	-725.92996	0.19706	31.47	29.96
**FAF3**	-727.10700	-725.92743	0.19677	36.73	35.88
**FAF24**	-727.10818	-725.92762	0.19735	35.15	36.89
**FAF16**	-727.10741	-725.92733	0.19725	36.92	37.37
**FAF8**	-727.10621	-725.92575	0.19732	40.25	41.72
**FAF27**	-727.09934	-725.92137	0.19678	56.90	51.81
**FAF26**	-727.10011	-725.92127	0.19674	54.76	51.97
**FAF25**	-727.10054	-725.92238	0.19659	53.23	48.65
**FAF5**	-727.09752	-725.91903	0.19681	61.74	58.02

2.2. Structures and Cartesian Coordinates

2.2.1. Graphical representation of the equilibrium geometries of the identified FAF structures

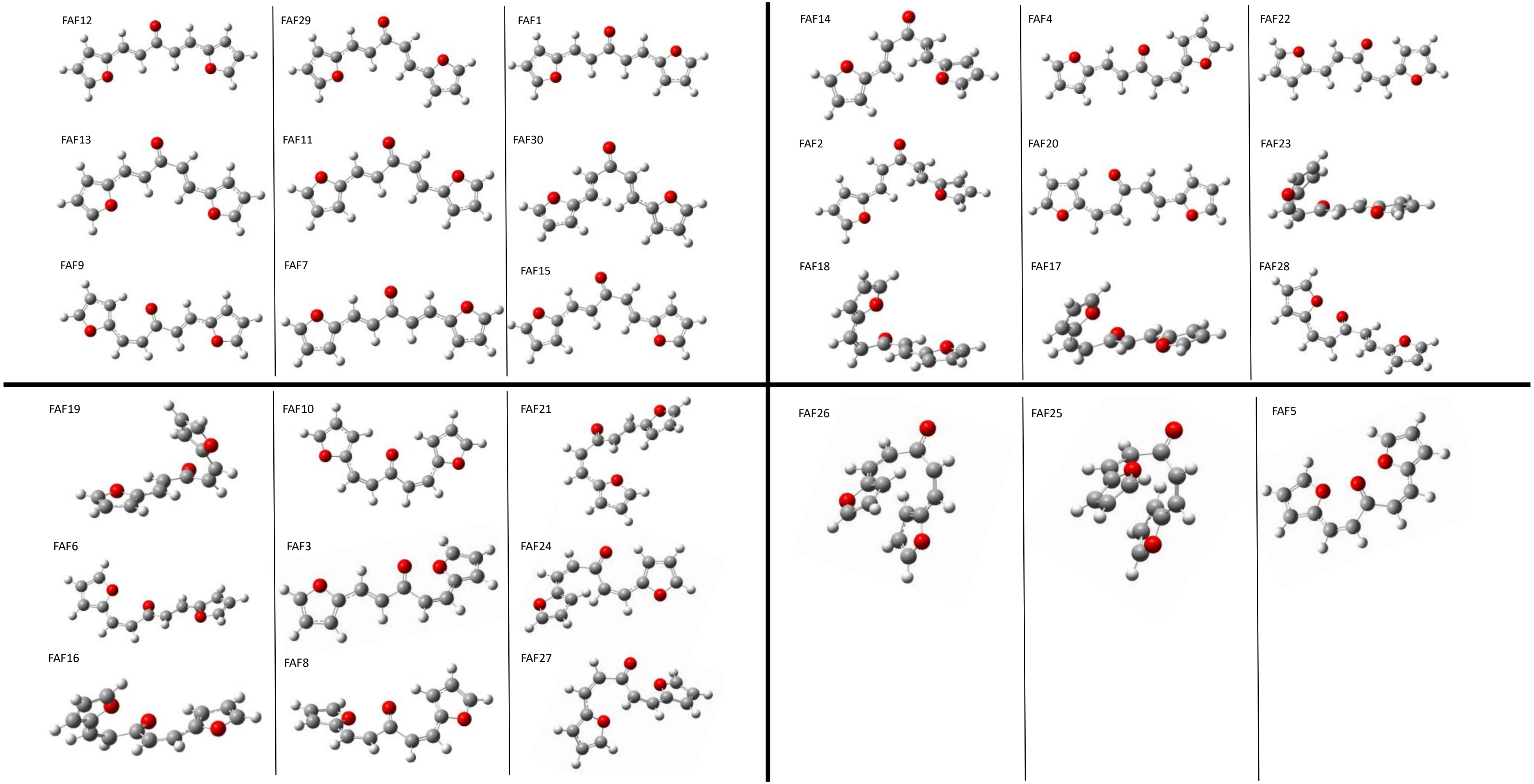

Figure S1. Graphical representation of the 30 structures of FAF studied in this work following the order of Table S1.

2.2.2. Cartesian Coordinates of the identified FAF species at the B3/junDZ level of theory.

FAF1				FAF16			
C	5.05124	-0.71393	0.00000	C	-3.18647	0.52178	-0.62150
C	5.97841	0.37351	0.00000	C	-4.50620	0.02673	-0.83440
C	5.22061	1.51389	0.00000	C	-4.62796	-1.09473	-0.05518
C	3.78672	-0.16204	0.00000	C	-2.57655	-0.33289	0.27967
C	2.48744	-0.76358	0.00000	C	-1.31029	-0.47268	0.93595
C	1.30130	-0.11119	0.00000	C	-0.15442	0.24658	0.88072
C	0.02475	-0.85976	0.00000	C	0.11732	1.46828	0.11598
C	-1.20233	-0.03312	0.00000	C	1.45833	2.10643	0.26606
C	-2.42950	-0.60464	0.00000	C	2.69252	1.55060	0.25846
C	-3.68162	0.09566	0.00000	C	3.10186	0.18947	0.01712
C	-4.05340	1.42519	0.00000	C	4.32944	-0.41932	0.17449
C	-5.47983	1.45697	0.00000	C	4.17592	-1.77774	-0.24569
C	-5.88885	0.14847	0.00000	C	2.87235	-1.90363	-0.64315
O	-4.81836	-0.68506	0.00000	O	2.21163	-0.72376	-0.49236
O	3.89452	1.21057	0.00000	O	-3.48241	-1.33148	0.62394
O	-0.01384	-2.09413	0.00000	O	-0.72985	2.05024	-0.57050
H	5.46452	2.57161	0.00000	H	-5.43624	-1.79986	0.11441
H	7.06364	0.32091	0.00000	H	-5.27123	0.44802	-1.48114
H	5.27513	-1.77747	0.00000	H	-2.70977	1.39395	-1.05192
H	2.46828	-1.85731	0.00000	H	-1.29651	-1.34129	1.60218
H	1.26722	0.98050	0.00000	H	0.66558	-0.11483	1.50180
H	-1.08623	1.05385	0.00000	H	1.39882	3.19653	0.33299
H	-2.48279	-1.69743	0.00000	H	3.54284	2.22267	0.41067
H	-3.37699	2.27538	0.00000	H	5.22966	0.05863	0.55210
H	-6.12286	2.33296	0.00000	H	4.93238	-2.55764	-0.25976
H	-6.86367	-0.32869	0.00000	H	2.28789	-2.72332	-1.04854

FAF2				FAF17			
C	2.85058	-0.29196	0.06593	C	2.84823	-2.63319	-0.30545
C	4.03580	0.02698	-0.56165	C	3.42209	-1.59399	0.49159
C	4.92475	-1.07022	-0.34735	C	1.83743	-2.04637	-1.01778
C	4.22763	-1.98634	0.39502	C	2.71121	-0.44310	0.22128
C	1.57782	0.36070	0.22835	C	-3.88613	0.20179	-0.53466
C	1.26739	1.58823	-0.24975	C	-4.48476	-0.99472	-0.03044
C	0.00000	2.30946	0.00000	C	-3.51267	-1.62260	0.70081
C	-1.26739	1.58823	0.24975	C	-2.58578	0.22305	-0.07524
C	-1.57782	0.36070	-0.22835	O	1.74444	-0.72412	-0.71304
C	-2.85058	-0.29196	-0.06593	O	-2.35825	-0.90219	0.68461
C	-4.03580	0.02698	0.56165	C	2.87540	0.89766	0.72375
C	-4.92475	-1.07022	0.34735	C	2.08127	1.98834	0.60306
C	-4.22763	-1.98634	-0.39502	C	0.72721	2.12402	-0.00161
O	-2.97320	-1.53268	-0.65395	C	-0.27652	1.06844	0.25119
O	2.97320	-1.53268	0.65395	C	-1.52384	1.16707	-0.26223
O	0.00000	3.54405	0.00000	O	0.45463	3.15544	-0.62002
H	-5.94891	-1.16938	0.69682	H	1.12946	-2.41142	-1.75483
H	-4.47309	-2.96311	-0.79940	H	3.14809	-3.67653	-0.35171
H	-4.24131	0.94246	1.10933	H	4.25371	-1.67598	1.18665
H	-0.84662	-0.20187	-0.81291	H	3.82905	1.03364	1.24346
H	-2.01842	2.18760	0.77172	H	2.48431	2.94373	0.95060
H	2.01842	2.18760	-0.77172	H	0.00156	0.22034	0.87586
H	0.84662	-0.20187	0.81291	H	-1.76187	2.03611	-0.88180
H	4.24131	0.94246	-1.10933	H	-4.34700	0.96234	-1.15950
H	5.94891	-1.16938	-0.69682	H	-5.50121	-1.34551	-0.18662
H	4.47309	-2.96311	0.79940	H	-3.48608	-2.54696	1.26921
FAF3				FAF18			
C	-5.55799	-0.60846	-0.18597	C	2.25612	-2.79861	-0.48641
C	-4.96680	0.68022	-0.05034	C	2.63992	-2.02742	0.65702
C	-3.59346	0.49424	-0.02830	C	1.70443	-1.91197	-1.37016
C	-4.50851	-1.49098	-0.24563	C	2.28399	-0.72213	0.39470
C	4.30891	1.01847	-0.24062	C	-2.82050	-0.89395	1.13949
C	5.65608	0.54906	-0.28359	C	-4.07761	-1.13357	0.50199
C	3.50773	-0.08163	-0.01041	C	-4.09301	-0.33595	-0.61049
C	5.59342	-0.80454	-0.07754	C	-2.15338	0.03571	0.36964
O	-3.32515	-0.84268	-0.15193	O	1.71689	-0.65302	-0.85269
O	4.30626	-1.20148	0.08888	O	-2.93808	0.37690	-0.70660
C	-2.57421	1.49859	0.06939	C	2.43261	0.47609	1.18579
C	-1.21578	1.47022	0.14481	C	1.90898	1.70427	0.98081
C	-0.29499	0.31628	0.24160	C	1.00745	2.16656	-0.12408
C	1.14081	0.66346	0.07937	C	-0.31443	1.55145	-0.32209
C	2.09580	-0.29285	0.13965	C	-0.86166	0.64145	0.51671
O	-0.65902	-0.83925	0.45960	O	1.36121	3.12265	-0.81337
H	-4.44701	-2.57001	-0.34813	H	1.28445	-2.00873	-2.36608
H	-6.61481	-0.85608	-0.23391	H	2.37836	-3.86769	-0.63718
H	-5.47550	1.63818	0.02342	H	3.11702	-2.38457	1.56605
H	-3.02112	2.49836	0.05883	H	3.09633	0.34312	2.04551
H	-0.72680	2.44843	0.14987	H	2.23307	2.51442	1.64039
H	1.40406	1.70938	-0.09841	H	-0.86268	1.91345	-1.19472
H	1.77352	-1.32311	0.31849	H	-0.30164	0.30854	1.39353
H	3.96525	2.04169	-0.36405	H	-2.44299	-1.34642	2.05277
H	6.55682	1.13495	-0.44591	H	-4.86777	-1.80650	0.82336
H	6.34354	-1.58709	-0.02426	H	-4.81966	-0.16798	-1.39905

FAF4				FAF19			
C	-4.85909	-1.48107	0.00000	C	-2.07865	2.11799	1.36385
C	-3.49564	-1.06758	0.00000	C	-1.81353	0.74102	1.08253
C	-5.61033	-0.33353	0.00000	C	-2.68757	2.62544	0.24844
C	-3.49523	0.31658	0.00000	C	-2.27484	0.49740	-0.19140
C	4.36652	1.04530	0.00000	C	2.78245	1.10016	-1.08257
C	5.71963	0.59214	0.00000	C	4.03735	1.39687	-0.46673
C	3.56655	-0.07973	0.00000	C	4.14611	0.54061	0.59636
C	5.66160	-0.77741	0.00000	C	2.20992	0.08045	-0.35085
O	-4.81450	0.75938	0.00000	O	-2.81949	1.65984	-0.70026
O	4.37179	-1.19925	0.00000	O	3.05114	-0.26125	0.68219
C	-2.54214	1.38499	0.00000	C	-2.35903	-0.65082	-1.06649
C	-1.17846	1.40473	0.00000	C	-1.73423	-1.84281	-0.95515
C	-0.24608	0.26973	0.00000	C	-0.77617	-2.29317	0.10510
C	1.18941	0.63683	0.00000	C	0.50741	-1.60244	0.30223
C	2.15115	-0.31543	0.00000	C	0.96335	-0.60999	-0.49844
O	-0.60905	-0.91435	0.00000	O	-1.05729	-3.28872	0.77313
H	-6.67959	-0.14370	0.00000	H	-3.07298	3.60551	-0.01391
H	-5.23863	-2.49936	0.00000	H	-1.85337	2.65919	2.27888
H	-2.59781	-1.67391	0.00000	H	-1.35142	0.01504	1.74368
H	-3.03314	2.36346	0.00000	H	-3.06041	-0.51807	-1.89600
H	-0.71668	2.39509	0.00000	H	-2.01766	-2.63668	-1.65193
H	1.44943	1.69828	0.00000	H	1.10507	-1.96996	1.13960
H	1.83183	-1.36186	0.00000	H	0.35279	-0.27315	-1.33920
H	4.01815	2.07437	0.00000	H	2.34358	1.57428	-1.95662
H	6.62129	1.19868	0.00000	H	4.76532	2.14457	-0.76903
H	6.41723	-1.55648	0.00000	H	4.90780	0.38407	1.35366
FAF5				FAF20			
C	-4.27330	1.63091	0.10326	C	5.20534	-0.93781	0.00000
C	-4.30525	0.20851	0.21030	C	3.78011	-0.93479	0.00000
C	-2.94767	1.96414	-0.00420	C	5.59391	0.37714	0.00000
C	-2.99407	-0.22837	0.14640	C	3.38022	0.38987	0.00000
O	-2.16838	0.85600	0.02158	C	-4.36808	-1.05753	0.00000
C	4.30525	0.20851	0.21030	C	-5.71302	-0.57797	0.00000
C	2.99407	-0.22837	0.14640	C	-5.63057	0.78956	0.00000
C	4.27330	1.63091	0.10326	C	-3.54921	0.05208	0.00000
C	2.94767	1.96414	-0.00420	O	4.51641	1.19385	0.00000
O	2.16838	0.85600	0.02158	O	-4.33136	1.18740	0.00000
C	-2.48858	-1.57025	0.24037	C	2.16245	1.14584	0.00000
C	-1.24394	-2.09160	0.09272	C	0.84645	0.79148	0.00000
C	0.00000	-1.41655	-0.36166	C	0.27344	-0.56034	0.00000
C	1.24394	-2.09160	0.09272	C	-1.19711	-0.71961	0.00000
C	2.48858	-1.57025	0.24037	C	-2.12649	0.26503	0.00000
O	0.00000	-0.43975	-1.10334	O	0.97008	-1.58551	0.00000
H	-2.42140	2.90823	-0.10352	H	6.56279	0.86756	0.00000
H	-5.11463	2.31839	0.10582	H	5.86198	-1.80374	0.00000
H	-5.17957	-0.42822	0.31954	H	3.09788	-1.77590	0.00000
H	-3.27841	-2.27516	0.51973	H	2.36189	2.22224	0.00000
H	-1.13705	-3.15375	0.33176	H	0.14270	1.62411	0.00000
H	1.13705	-3.15375	0.33176	H	-1.50835	-1.76789	0.00000
H	3.27841	-2.27516	0.51973	H	-1.83088	1.31622	0.00000
H	5.17957	-0.42822	0.31954	H	-4.03892	-2.09280	0.00000
H	5.11463	2.31839	0.10582	H	-6.62539	-1.16820	0.00000
H	2.42140	2.90823	-0.10352	H	-6.37103	1.58297	0.00000

FAF6				FAF21			
C	5.61179	-0.44441	0.00000	C	3.00471	-2.59188	0.28068
C	4.93038	0.80573	0.00000	C	3.56297	-1.51623	-0.47869
C	4.62680	-1.40056	0.00000	C	1.94899	-2.05848	0.96920
C	3.57219	0.52639	0.00000	C	2.79945	-0.39884	-0.21200
C	-4.55369	-1.28596	-0.00000	C	-2.54135	-1.14185	-0.77409
C	-5.78077	-0.55257	0.00000	C	-3.86044	-1.66832	-0.63040
C	-5.42838	0.77023	0.00000	C	-4.54583	-0.77010	0.14547
C	-3.53270	-0.35837	0.00000	C	-2.50675	0.04800	-0.07553
O	3.39986	-0.83225	0.00000	O	1.81334	-0.73496	0.68359
O	-4.07453	0.90724	0.00000	O	-3.74435	0.27032	0.48943
C	2.49040	1.46762	-0.00000	C	2.93015	0.95759	-0.68280
C	1.13222	1.37182	-0.00000	C	2.09462	2.01698	-0.56521
C	0.26003	0.17809	-0.00000	C	0.71972	2.09292	0.00383
C	-1.19429	0.48534	-0.00000	C	-0.23748	1.01008	-0.30382
C	-2.10827	-0.51202	-0.00000	C	-1.50194	1.04262	0.17465
O	0.67375	-0.98200	-0.00000	O	0.39554	3.09769	0.63962
H	4.64225	-2.48611	0.00000	H	1.23375	-2.46353	1.67790
H	6.68397	-0.62020	0.00000	H	3.34484	-3.62311	0.31975
H	5.37005	1.79999	-0.00000	H	4.42040	-1.55274	-1.14569
H	2.88190	2.49051	-0.00000	H	3.89368	1.13742	-1.16992
H	0.59910	2.32632	-0.00000	H	2.47304	2.99267	-0.88269
H	-1.51459	1.52930	0.00000	H	0.10070	0.19440	-0.94338
H	-1.73917	-1.54191	-0.00000	H	-1.79497	1.88457	0.80816
H	-4.42523	-2.36518	-0.00000	H	-1.71329	-1.58157	-1.32307
H	-6.79202	-0.94999	0.00000	H	-4.25404	-2.59190	-1.04640
H	-5.99786	1.69428	0.00000	H	-5.56311	-0.73192	0.52181
FAF7				FAF22			
C	4.15085	-1.39911	0.00000	C	-5.15305	1.02805	0.00000
C	5.57751	-1.38359	0.00000	C	-3.72903	0.96773	0.00000
C	5.94281	-0.06218	0.00000	C	-5.59424	-0.27015	0.00000
C	3.73511	-0.08262	0.00000	C	-3.38279	-0.37189	0.00000
C	2.46073	0.57612	0.00000	C	4.56430	-1.26767	0.00000
C	1.25261	-0.03465	0.00000	C	5.79785	-0.54448	0.00000
C	0.00000	0.75330	0.00000	C	5.45648	0.78097	0.00000
C	-1.25261	-0.03465	0.00000	C	3.55264	-0.33029	0.00000
C	-2.46073	0.57612	0.00000	O	-4.55043	-1.12964	0.00000
C	-3.73511	-0.08262	0.00000	O	4.10357	0.92844	0.00000
C	-4.15085	-1.39911	0.00000	C	-2.19641	-1.17642	0.00000
C	-5.57751	-1.38359	0.00000	C	-0.86728	-0.87537	0.00000
C	-5.94281	-0.06218	0.00000	C	-0.23957	0.45276	0.00000
O	-4.84532	0.73526	0.00000	C	1.23653	0.55340	0.00000
O	4.84532	0.73526	0.00000	C	2.12306	-0.47044	0.00000
O	0.00000	1.98770	0.00000	O	-0.89649	1.50331	0.00000
H	6.90126	0.44708	0.00000	H	-6.58208	-0.72118	0.00000
H	6.24932	-2.23770	0.00000	H	-5.77424	1.91974	0.00000
H	3.50319	-2.27139	0.00000	H	-3.01348	1.78071	0.00000
H	2.47815	1.67008	0.00000	H	-2.43953	-2.24384	0.00000
H	1.17236	-1.12496	0.00000	H	-0.19839	-1.73637	0.00000
H	-1.17236	-1.12496	0.00000	H	1.60080	1.58347	0.00000
H	-2.47815	1.67008	0.00000	H	1.78416	-1.50841	0.00000
H	-3.50319	-2.27139	0.00000	H	4.42913	-2.34618	0.00000
H	-6.24932	-2.23770	0.00000	H	6.80557	-0.95055	0.00000
H	-6.90126	0.44708	0.00000	H	6.03220	1.70099	0.00000

FAF8				FAF23			
C	4.58205	-0.04578	0.12137	C	-2.17170	-2.25941	1.00550
C	4.70603	-1.44513	-0.12131	C	-1.79203	-0.88473	0.91835
C	3.43214	-1.89220	-0.36303	C	-3.17400	-2.43663	0.09009
C	3.23573	0.26268	0.02375	C	-2.58308	-0.31049	-0.05421
C	2.60989	1.54897	0.15132	C	3.95071	0.07674	0.51662
C	1.31541	1.96277	0.15989	C	4.48243	-1.16067	0.03799
C	0.07187	1.15468	0.17649	C	3.46964	-1.75982	-0.66200
C	-1.13827	1.90603	-0.20033	C	2.64591	0.14917	0.07441
C	-2.41920	1.45137	-0.30241	O	-3.44230	-1.27062	-0.55292
C	-3.01441	0.16714	-0.07865	O	2.35241	-0.98392	-0.65036
C	-2.62431	-1.09836	0.32285	C	-2.77073	1.01386	-0.59673
C	-3.79423	-1.91259	0.32095	C	-1.95437	2.09380	-0.54403
C	-4.82689	-1.10359	-0.07822	C	-0.57212	2.20790	-0.01002
O	-4.38420	0.15043	-0.32373	C	0.37357	1.09438	-0.24058
O	2.53973	-0.87700	-0.27895	C	1.63441	1.14835	0.24868
O	0.05175	-0.03383	0.50937	O	-0.22917	3.25494	0.54458
H	3.01639	-2.86664	-0.59975	H	-3.77274	-3.29194	-0.20680
H	5.61235	-2.04437	-0.12010	H	-1.76106	-3.01626	1.66831
H	5.37766	0.66121	0.34277	H	-1.04199	-0.37306	1.51256
H	3.36184	2.34165	0.22509	H	-3.74751	1.14801	-1.07273
H	1.16489	3.04564	0.18194	H	-2.35884	3.04978	-0.88737
H	-0.98760	2.95998	-0.44580	H	0.04119	0.24033	-0.83092
H	-3.16326	2.18974	-0.61831	H	1.92517	2.02405	0.83564
H	-1.60671	-1.36584	0.58033	H	4.45760	0.83052	1.11343
H	-3.86497	-2.96489	0.58316	H	5.48282	-1.55656	0.18975
H	-5.88932	-1.27119	-0.22818	H	3.38950	-2.69722	-1.20341
FAF9				FAF24			
C	4.59894	-1.27547	0.00000	C	-3.30124	0.52345	-0.56916
C	5.81985	-0.53242	0.00000	C	-4.61433	0.01320	-0.78465
C	5.45679	0.78767	0.00000	C	-4.69568	-1.15561	-0.07223
C	3.57048	-0.35584	0.00000	C	-2.65390	-0.37062	0.26582
C	2.14782	-0.52032	0.00000	C	-1.37234	-0.52101	0.88831
C	1.22693	0.47152	0.00000	C	-0.22612	0.21559	0.84067
C	-0.22172	0.16092	0.00000	C	0.01417	1.47298	0.12360
C	-1.10838	1.33192	0.00000	C	1.36287	2.09611	0.23442
C	-2.47160	1.36744	0.00000	C	2.58785	1.52129	0.18801
C	-3.46721	0.33826	0.00000	C	2.95130	0.14847	-0.07318
C	-3.52328	-1.04465	0.00000	C	2.34619	-0.94913	-0.64741
C	-4.90237	-1.40305	0.00000	C	3.31518	-1.99958	-0.65959
C	-5.60682	-0.22626	0.00000	C	4.45046	-1.47733	-0.10076
O	-4.76751	0.83372	0.00000	O	4.25302	-0.18031	0.25183
O	4.10215	0.91400	0.00000	O	-3.53146	-1.40741	0.56780
O	-0.63143	-1.00840	0.00000	O	-0.86217	2.08133	-0.50149
H	6.01896	1.71619	0.00000	H	-5.48550	-1.88704	0.07104
H	6.83424	-0.92168	0.00000	H	-5.40211	0.45603	-1.38820
H	4.47929	-2.35572	0.00000	H	-2.85342	1.43151	-0.95410
H	1.78685	-1.55290	0.00000	H	-1.33221	-1.41879	1.51376
H	1.53941	1.51768	0.00000	H	0.61307	-0.16184	1.42655
H	-0.60625	2.30233	0.00000	H	1.32052	3.18699	0.29726
H	-2.92260	2.36497	0.00000	H	3.45445	2.17798	0.31541
H	-2.65030	-1.68622	0.00000	H	1.33248	-0.98760	-1.03274
FAF10				FAF25			
C	2.70856	1.15886	0.00000	C	-1.32070	-2.20533	-0.98355
C	3.88713	1.95997	0.00000	C	-1.81149	-2.85975	0.18892
C	4.94562	1.08813	0.00000	C	-2.20806	-1.86152	1.03779

C	3.11924	-0.16249	0.00000	C	-1.43149	-0.84919	-0.75964
C	2.52961	-1.46825	0.00000	C	-1.08087	0.26814	-1.59814
C	1.23695	-1.90145	0.00000	C	-0.86359	1.56622	-1.28648
C	0.00000	-1.10472	0.00000	C	-0.85598	2.24237	0.03717
C	-1.23695	-1.90145	0.00000	C	-0.34234	1.60186	1.27506
C	-2.52961	-1.46825	0.00000	C	0.69703	0.75694	1.45927
C	-3.11924	-0.16249	0.00000	C	1.66566	0.23588	0.52324
C	-2.70856	1.15886	0.00000	C	2.17259	0.60405	-0.70262
C	-3.88713	1.95997	0.00000	C	3.18595	-0.34660	-1.03843
C	-4.94562	1.08813	0.00000	C	3.23944	-1.23070	0.00548
O	-4.51027	-0.19205	0.00000	O	2.33815	-0.88917	0.96226
O	4.51027	-0.19205	0.00000	O	-1.98875	-0.64249	0.47701
O	0.00000	0.13719	0.00000	O	-1.28179	3.39611	0.10685
H	6.02295	1.22466	0.00000	H	-2.66019	-1.85412	2.02436
H	3.94675	3.04507	0.00000	H	-1.87122	-3.92809	0.37740
H	1.67171	1.47031	0.00000	H	-0.91780	-2.66826	-1.88055
H	3.29311	-2.25286	0.00000	H	-0.99351	-0.00777	-2.65373
H	1.10212	-2.98536	0.00000	H	-0.71868	2.26654	-2.11403
H	-1.10212	-2.98536	0.00000	H	-0.87489	1.94246	2.16673
H	-3.29311	-2.25286	0.00000	H	0.87108	0.41020	2.48306
H	-1.67171	1.47031	0.00000	H	1.86395	1.46458	-1.28594
H	-3.94675	3.04507	0.00000	H	3.80244	-0.36634	-1.93319
H	-6.02295	1.22466	0.00000	H	3.84003	-2.11141	0.20968
		FAF11				FAF26	
C	-3.96904	-1.72451	0.00000	C	2.02159	-0.23284	-0.90961
C	-5.37450	-1.46207	0.00000	C	2.57007	-1.51652	-1.21175
C	-5.50226	-0.09924	0.00000	C	2.53689	-2.22933	-0.04290
C	-3.33482	-0.49972	0.00000	C	1.68208	-0.24361	0.42529
C	-1.94286	-0.14637	0.00000	C	1.15576	0.69299	1.38654
C	-1.45416	1.11693	0.00000	C	0.46990	1.84899	1.22621
C	-0.02806	1.50108	0.00000	C	0.00000	2.54172	0.00000
C	1.01355	0.44727	0.00000	C	-0.46990	1.84899	-1.22621
C	2.32487	0.77929	0.00000	C	-1.15576	0.69299	-1.38654
C	3.42361	-0.14524	0.00000	C	-1.68208	-0.24361	-0.42529
C	3.54046	-1.52038	0.00000	C	-2.02159	-0.23284	0.90961
C	4.93633	-1.81833	0.00000	C	-2.57007	-1.51652	1.21175
C	5.58239	-0.60956	0.00000	C	-2.53689	-2.22933	0.04290
O	4.68582	0.40912	0.00000	O	-2.01617	-1.47577	-0.95789
O	-4.27959	0.49779	0.00000	O	2.01617	-1.47577	0.95789
O	0.26937	2.69974	0.00000	O	0.00000	3.77465	0.00000
H	-6.35571	0.57125	0.00000	H	2.83583	-3.23752	0.22608
H	-6.18512	-2.18545	0.00000	H	2.94598	-1.86472	-2.16998
H	-3.47666	-2.69355	0.00000	H	1.90052	0.60276	-1.58959
H	-1.27137	-1.00738	0.00000	H	1.38005	0.40120	2.41781
H	-2.14102	1.96640	0.00000	H	0.28144	2.44192	2.12565
H	0.71298	-0.60141	0.00000	H	-0.28144	2.44192	-2.12565
H	2.58397	1.84196	0.00000	H	-1.38005	0.40120	-2.41781
H	2.71787	-2.23008	0.00000	H	-1.90052	0.60276	1.58959
H	5.40441	-2.79903	0.00000	H	-2.94598	-1.86472	2.16998
H	6.62901	-0.32241	0.00000	H	-2.83583	-3.23752	-0.22608

FAF12				FAF27			
C	5.01974	0.65662	0.00000	C	4.07176	-0.58163	0.03868
C	5.91165	-0.45996	0.00000	C	3.76126	-1.97269	0.15359
C	5.11766	-1.57549	0.00000	C	2.40595	-2.04156	0.33431
C	3.73819	0.14545	0.00000	C	2.87915	0.10432	0.14207
C	2.45866	0.78814	0.00000	C	2.60840	1.52005	0.12115
C	1.25247	0.17360	0.00000	C	1.43690	2.19232	0.02281
C	0.00000	0.96136	0.00000	C	0.05142	1.68462	-0.20049
C	-1.25247	0.17360	0.00000	C	-0.13762	0.57223	-1.16048
C	-2.45866	0.78814	0.00000	C	-1.21245	-0.24008	-1.31405
C	-3.73819	0.14545	0.00000	C	-2.39443	-0.43822	-0.52109
C	-5.01974	0.65662	0.00000	C	-3.51106	-1.20453	-0.80296
C	-5.91165	-0.45996	0.00000	C	-4.37984	-1.10069	0.32447
C	-5.11766	-1.57549	0.00000	C	-3.72885	-0.29244	1.22041
O	-3.80209	-1.22977	0.00000	O	-2.53372	0.11073	0.72498
O	3.80209	-1.22977	0.00000	O	1.86069	-0.79594	0.33238
O	0.00000	2.19690	0.00000	O	-0.88442	2.29596	0.31146
H	5.32744	-2.64050	0.00000	H	1.71042	-2.86091	0.48495
H	6.99802	-0.44222	0.00000	H	4.45037	-2.81198	0.11629
H	5.27756	1.71245	0.00000	H	5.05039	-0.13203	-0.10826
H	2.47454	1.88192	0.00000	H	3.51533	2.11971	0.24861
H	1.18296	-0.91633	0.00000	H	1.46640	3.27614	0.16606
H	-1.18296	-0.91633	0.00000	H	0.68618	0.42805	-1.86392
H	-2.47454	1.88192	0.00000	H	-1.18767	-0.91668	-2.17442
H	-5.27756	1.71245	0.00000	H	-3.67707	-1.76786	-1.71784
H	-6.99802	-0.44222	0.00000	H	-5.35523	-1.56023	0.45867
H	-5.32744	-2.64050	0.00000	H	-3.97595	0.07404	2.21184

FAF13				FAF28			
C	-4.83763	0.22640	0.00000	C	5.03836	-0.06642	-1.10543
C	-5.43922	-1.07044	0.00000	C	4.49757	-0.99231	-0.16051
C	-4.40363	-1.96563	0.00000	C	4.02886	0.81753	-1.38034
C	-3.47199	0.03404	0.00000	C	3.19765	-0.59772	0.08502
C	-2.38005	0.96187	0.00000	C	-4.05376	-1.33317	-0.46863
C	-1.06504	0.64410	0.00000	C	-5.29666	-0.69630	-0.77570
C	-0.03870	1.71140	0.00000	C	-5.09252	0.64229	-0.57579
C	1.39112	1.34522	0.00000	C	-3.17490	-0.33604	-0.10132
C	1.89567	0.08842	0.00000	O	2.91305	0.50685	-0.67219
C	3.29666	-0.23607	0.00000	O	-3.81582	0.87788	-0.16825
C	4.46535	0.49616	0.00000	C	2.19995	-1.18679	0.93950
C	5.54079	-0.44289	0.00000	C	0.97334	-0.73717	1.29115
C	4.96256	-1.68503	0.00000	C	0.38801	0.60931	1.01659
O	3.60820	-1.57911	0.00000	C	-1.03643	0.68958	0.63683
O	-3.20729	-1.31705	0.00000	C	-1.79894	-0.37953	0.30688
O	-0.34981	2.90772	0.00000	O	1.04690	1.63884	1.14914
H	-4.35445	-3.04996	0.00000	H	3.94960	1.68390	-2.02928
H	-6.49880	-1.31087	0.00000	H	6.03970	-0.05473	-1.52697
H	-5.33886	1.19076	0.00000	H	4.99817	-1.84377	0.29350
H	-2.65372	2.02065	0.00000	H	2.50246	-2.16711	1.32058
H	-0.76132	-0.40256	0.00000	H	0.34892	-1.41099	1.88469
H	2.05944	2.21050	0.00000	H	-1.44569	1.70156	0.59603
H	1.23306	-0.77951	0.00000	H	-1.36697	-1.38348	0.32423
H	4.53835	1.58005	0.00000	H	-3.82526	-2.39500	-0.51098
H	6.60602	-0.22826	0.00000	H	-6.22059	-1.16577	-1.10191
H	5.36076	-2.69463	0.00000	H	-5.72633	1.51774	-0.67530

FAF14				FAF29			
C	3.16084	-1.70160	0.74346	C	-4.85700	0.31579	0.00000
C	4.50062	-1.91381	0.29718	C	-5.51280	-0.95463	0.00000
C	4.81973	-0.82443	-0.45453	C	-4.51606	-1.89267	0.00000
C	2.76267	-0.49518	0.22882	C	-3.50079	0.06620	0.00000
C	1.52366	0.22757	0.33981	C	-2.37082	0.94764	0.00000
C	1.28340	1.43542	-0.20659	C	-1.07026	0.57584	0.00000
C	0.04630	2.21806	0.00327	C	0.00069	1.59984	0.00000
C	-1.25063	1.55069	0.25066	C	1.41439	1.17425	0.00000
C	-1.58792	0.33239	-0.21333	C	1.86155	-0.10450	0.00000
C	-2.88945	-0.27121	-0.06555	C	3.24099	-0.50326	0.00000
C	-4.07293	0.10978	0.51212	C	3.83399	-1.74851	0.00000
C	-4.98985	-0.96319	0.30943	C	5.24740	-1.53317	0.00000
C	-4.30516	-1.92163	-0.37587	C	5.42065	-0.17533	0.00000
O	-3.03046	-1.52104	-0.61272	O	4.21871	0.46230	0.00000
O	3.77848	0.04751	-0.50845	O	-3.29315	-1.29502	0.00000
O	0.09355	3.44512	-0.02791	O	-0.26290	2.80702	0.00000
H	5.70463	-0.53185	-0.99379	H	-4.51282	-2.97810	0.00000
H	5.14025	-2.75593	0.50427	H	-6.58159	-1.15008	0.00000
H	2.56194	-2.34902	1.36412	H	-5.31711	1.30043	0.00000
H	0.76448	-0.26081	0.94039	H	-2.59990	2.01697	0.00000
H	2.06493	1.95529	-0.74953	H	-0.81135	-0.48282	0.00000
H	-1.98409	2.17645	0.74919	H	2.12750	2.00179	0.00000
H	-0.86603	-0.25697	-0.76804	H	1.16144	-0.94233	0.00000
H	-4.26136	1.04437	1.01540	H	3.30903	-2.70029	0.00000
H	-6.01843	-1.01753	0.62664	H	6.03342	-2.28323	0.00000
H	-4.57092	-2.89530	-0.75077	H	6.29611	0.46616	0.00000
FAF15				FAF30			
C	3.38417	-0.23083	0.00000	C	-4.62093	-1.84621	0.29829
C	4.53056	0.53564	0.00000	C	-3.26086	-1.69234	0.71416
C	5.63330	-0.37126	0.00000	C	-4.92307	-0.72301	-0.42255
C	5.09227	-1.62995	0.00000	C	-2.82663	-0.48247	0.21616
C	1.97407	0.05177	0.00000	C	3.25999	-1.69360	-0.71214
C	1.43113	1.29231	0.00000	C	4.62056	-1.84684	-0.29773
C	-0.00901	1.61820	0.00000	C	4.92360	-0.72250	0.42100
C	-1.00547	0.52307	0.00000	C	2.82647	-0.48280	-0.21574
C	-2.32953	0.80088	0.00000	O	-3.84939	0.11184	-0.48289
C	-3.38829	-0.16885	0.00000	O	3.85013	0.11258	0.48109
C	-3.44670	-1.54776	0.00000	C	-1.56020	0.19043	0.31381
C	-4.82862	-1.90439	0.00000	C	-1.27569	1.41076	-0.19812
C	-5.52533	-0.72402	0.00000	C	0.00010	2.13615	0.00002
O	-4.67287	0.33178	0.00000	C	1.27623	1.41133	0.19705
O	3.73513	-1.56421	0.00000	C	1.56005	0.19004	-0.31313
O	-0.35262	2.80495	0.00000	O	-0.00040	3.36971	0.00089
H	-5.25463	-2.90407	0.00000	H	-5.81689	-0.38150	-0.93484
H	-6.58322	-0.48157	0.00000	H	-5.28885	-2.67756	0.50637
H	-2.59476	-2.22198	0.00000	H	-2.66808	-2.38196	1.30963
H	-2.63296	1.85173	0.00000	H	-0.80479	-0.35473	0.88411
H	-0.66106	-0.51193	0.00000	H	-2.05352	1.98900	-0.70265
H	2.07339	2.17711	0.00000	H	2.05462	1.99038	0.69978
H	1.33905	-0.83655	0.00000	H	0.80390	-0.35605	-0.88154
H	4.57155	1.62122	0.00000	H	2.66643	-2.38418	-1.30571
H	6.69168	-0.12510	0.00000	H	5.28819	-2.67856	-0.50523
H	5.52026	-2.62728	0.00000	H	5.81804	-0.38028	0.93173

3. Transitions States for the intercoversion between FAF species

3.1. Energies

Table S2. B3/junDZ absolute and relative energies of the TSs located on the interconversion PES. For completeness, the connected minima are also reported.

	CONNECTED MINIMA		IMAGINARY FREQUENCY (cm^{-1})	ELE. ENERGY (Hartree)	ZPE (Hartree)	RELATIVE ENERGIES (kJ/mol)
**TS1**	FAF11	FAF1	-121.5	-727.10497	0.19577	33.39
**TS2**	FAF1	FAF12	-151.6	-727.10459	0.19613	35.33
**TS3**	FAF1	FAF7	-152.2	-727.10459	0.19592	34.80
**TS4**	FAF10	FAF8	-74.3	-727.09771	0.19668	54.85
**TS5**	FAF9	FAF4	-148.9	-727.10186	0.19654	43.60
**TS6**	FAF4	FAF3	-80.4	-727.09781	0.19603	52.88
**TS7**	FAF1	FAF13	-145.1	-727.10503	0.19575	33.19
**TS8**	FAF13	FAF14	-93.7	-727.10741	0.19598	27.54
**TS9**	FAF2	FAF15	-114.5	-727.10528	0.19577	32.57
**TS10**	FAF14	FAF28	-106.2	-727.09942	0.19601	48.62
**TS11**	FAF2	FAF14	-109.9	-727.09899	0.19601	49.74
**TS12**	FAF6	FAF3	-150.0	-727.09314	0.19621	65.60
**TS13**	FAF9	FAF4	-79.4	-727.10020	0.19624	47.15
**TS14**	FAF9	FAF28	-119.7	-727.09611	0.19596	57.16
**TS15**	FAF28	FAF6	-104.0	-727.09606	0.19596	57.30
**TS16**	FAF8	FAF5	-72.0	-727.08880	0.19622	77.04
**TS17**	FAF29	FAF12	-120.5	-727.10711	0.19595	28.25
**TS18**	FAF29	FAF30	-90.2	-727.10889	0.19614	24.06
**TS19**	FAF14	FAF11	-110.2	-727.10676	0.19592	29.10
**TS20**	FAF15	FAF7	-145.2	-727.10287	0.19557	38.38
**TS21**	FAF28	FAF30	-104.8	-727.10102	0.19617	44.83
**TS22**	FAF10	FAF24	-53.6	-727.10430	0.19667	37.52
**TS23**	FAF24	FAF16	-59.2	-727.09888	0.19697	52.55
**TS24**	FAF16	FAF8	-40.3	-727.10379	0.19664	38.77

4. Comparison of the gas-phase and PCM structures

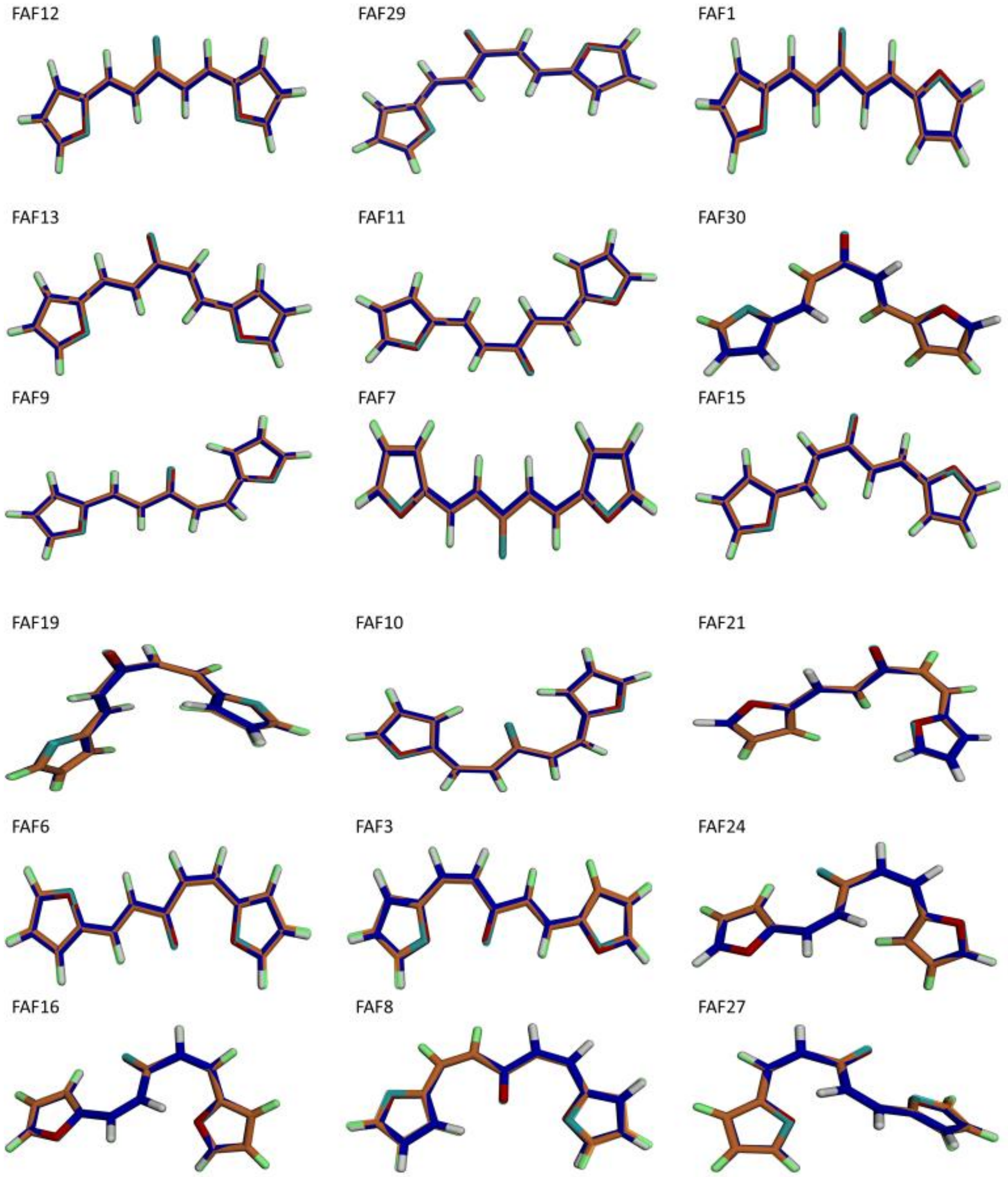

Figure S2. Overlay of PCM (C: bright orange, O: cyan, H: bright green) and gas-phase (C: dark blue, O: dark red, H: light grey) geometries for the 30 FAF structures investigated in this work, displayed in the order given in Table S1. These structures were optimized at the B3LYP-D3/jun-cc-pVDZ level.

Table S3. RMSD analysis of structural differences between gas-phase and PCM optimized geometries (given in Figure S2).

Conformer	RMSD (Å)
FAF1	0.0357
FAF2	0.0340
FAF3	0.0519
FAF4	0.0289
FAF5	0.0615
FAF6	0.0474
FAF7	0.0321
FAF8	0.0886
FAF9	0.0312
FAF10	0.0192
FAF11	0.0273
FAF12	0.0300
FAF13	0.0178
FAF14	0.0321
FAF15	0.0210
FAF16	0.0287
FAF17	0.0335
FAF18	0.0277
FAF19	0.0498
FAF20	0.0189
FAF21	0.0361
FAF22	0.0194
FAF23	0.0466
FAF24	0.0325
FAF25	0.0928
FAF26	0.0266
FAF27	0.0544
FAF28	0.0433
FAF29	0.0183
FAF30	0.0319

5. Infrared Spectrum:

5.1. Fundamental bands

Table S4. Harmonic Vibrational Frequencies and IR Intensities of FAF12 at the revDSD/junTZ level of theory and scaled frequencies based on B3/junDZ anharmonic corrections.

Mode	Wave Numbers (cm^{-1})	Scaled wave numbers (cm-1)	Intensity (km/mol)		Mode	Wave Numbers (cm^{-1})	Scaled Wave numbers (cm-1)	Intensity (km/mol)
(A1)	3292.8	3210.5	0.01		(B2)	1297.2	1264.8	104.94
(A1)	3271.6	3189.8	0.5		(B2)	1231.1	1200.3	92.24
(A1)	3260.7	3179.2	1.4		(B2)	1191.7	1161.9	1.04
(A1)	3202.1	3122.1	4.5		(B2)	1116.3	1088.4	135.05
(A1)	3186	3106.3	0.27		(B2)	1105.6	1077.9	171.77
(A1)	1726.8	1683.6	25.1		(B2)	1037.1	1011.2	162.72
(A1)	1648.4	1607.2	157.4		(B2)	946.23	922.6	0.46
(A1)	1595.2	1555.3	10.58		(B2)	900.05	877.5	37.89
(A1)	1518.6	1480.6	31.95		(B2)	828.03	807.3	12.59
(A1)	1429.7	1394	2.28		(B2)	649.59	633.4	109.75
(A1)	1334.6	1301.2	0.21		(B2)	441.62	430.6	4.31
(A1)	1309	1276.2	22.32		(B2)	275.92	269.0	6.51
(A1)	1290.5	1258.2	0.23		(B2)	110.52	107.8	0.35
(A1)	1222.9	1192.3	4.01		(B1)	1018.1	992.61	62.42
(A1)	1192.1	1162.3	0.5		(B1)	902.78	880.2	11.67
(A1)	1111.1	1083.3	8.43		(B1)	888.7	866.5	1.63
(A1)	1039.3	1013.3	4.95		(B1)	831.9	811.1	23.37
(A1)	975.52	951.13	2.23		(B1)	764.46	745.3	127.38
(A1)	949.18	925.45	20.56		(B1)	731.64	713.3	3.51
(A1)	900.29	877.78	0.03		(B1)	27.55	-	1.7
(A1)	753.31	734.48	0.71		(B1)	100.41	-	0.9
(A1)	530.91	517.64	9.64		(B1)	134.86	-	2.54
(A1)	256.88	250.46	0.04		(B1)	339.15	330.7	5.82
(A1)	192.65	-	0.04		(B1)	606.3	591.1	16.72
(A1)	41.51	-	0.73		(B1)	641.77	625.7	0.19
(B2)	3292.8	3210.5	0.02		(A2)	1009.5	984.2	0
(B2)	3271.6	3189.8	0.08		(A2)	889.62	867.4	0
(B2)	3260.7	3179.2	5.02		(A2)	863	841.4	0
(B2)	3193.6	3113.8	1.1		(A2)	823.17	802.6	0
(B2)	3185.3	3105.7	0.44		(A2)	763.91	744.8	0
(B2)	1681.4	1639.4	861.72		(A2)	673.78	656.9	0
(B2)	1600.3	1560.3	146.51		(A2)	606.33	591.2	0
(B2)	1524.3	1486.2	16.24		(A2)	339.43	330.9	0
(B2)	1429.3	1393.6	33.65		(A2)	207.59	202.4	0
(B2)	1348.6	1314.9	180.39		(A2)	92.64	-	0
(B2)	1306.5	1273.8	31.69		(A2)	39.2	-	0

Table S5. Harmonic Vibrational Frequencies and IR Intensities of FAF29 at the revDSD/junTZ level of theory and scaled Frequencies based on B3/junDZ anharmonic corrections.

Mode	Wave numbers (cm^{-1})	Scaled wave numbers (cm^{-1})	Intensity (km/mol)		Mode	Wave numbers (cm^{-1})	Scaled wave numbers (cm^{-1})	Intensity (km/mol)
(A')	3293.9	3211.6	0		(A')	920.1	897.1	4.1
(A')	3292.8	3210.5	0		(A')	900.4	877.9	10.3
(A')	3271.6	3189.8	0.2		(A')	900.0	877.5	21.1
(A')	3271.4	3189.6	0.4		(A')	798.8	778.8	7.1
(A')	3260.8	3179.3	2.9		(A')	757.5	738.6	3.8
(A')	3259.7	3178.2	3.6		(A')	621.0	605.5	65.6
(A')	3224.3	3143.7	4.2		(A')	536.5	523.1	8.2
(A')	3200.7	3120.7	4.3		(A')	411.3	401.0	12.3
(A')	3195.2	3115.3	0		(A')	369.3	360.1	3.1
(A')	3189	3109.3	0.2		(A')	241.8	235.8	4.1
(A')	1709.5	1666.8	286.9		(A')	186.4	181.7	1.1
(A')	1686.1	1643.9	156.3		(A')	111.4	108.6	0.7
(A')	1656.8	1615.4	302.3		(A')	43.5	-	0.5
(A')	1599.3	1559.3	85		(A")	764.9	745.8	80.7
(A')	1594.3	1554.4	10.1		(A")	1015.8	990.4	62.2
(A')	1524.2	1486.1	28.6		(A")	1001.9	976.9	3.3
(A')	1517.2	1479.3	6.7		(A")	897.1	874.7	3
(A')	1431.6	1395.8	14.2		(A")	889.0	866.8	0
(A')	1429.1	1393.4	21.2		(A")	887.4	865.2	1.2
(A')	1367.1	1332.9	89.8		(A")	865.9	844.3	1.9
(A')	1322.7	1289.6	152.5		(A")	830.0	809.3	22.9
(A')	1313.7	1280.9	1.2		(A")	822.1	801.5	0.2
(A')	1309.7	1277.0	19.8		(A")	762.6	743.5	47.1
(A')	1297.2	1264.8	80.6		(A")	728.6	710.4	3.3
(A')	1290.4	1258.1	2.1		(A")	674.9	658.0	0.1
(A')	1245.2	1214.1	139		(A")	629.2	613.5	0.2
(A')	1222.8	1192.2	37.2		(A")	606.4	591.2	8.8
(A')	1202.8	1172.7	102.4		(A")	606.3	591.1	7.8
(A')	1191.3	1161.5	3.2		(A")	344.1	335.5	0.1
(A')	1185.6	1156.0	64.5		(A")	319.0	311.0	5.5
(A')	1110.5	1082.7	2.9		(A")	181.1	-	0.1
(A')	1108.2	1080.5	30.6		(A")	139.0	-	3.4
(A')	1038.8	1012.8	44.5		(A")	99.1	-	0
(A')	1038.3	1012.3	59.2		(A")	86.1	-	0.3
(A')	954.6	930.7	14.7		(A")	33.6	-	0.3
(A')	948.8	925.1	11.4		(A")	23.0	-	0.3

Table S6. Harmonic Vibrational Frequencies and IR Intensities of FAF1 at the revDSD/junTZ level of theory scaled frequencies based on B3/junDZ anharmonic corrections.

Mode	Wave numbers (cm^{-1})	Scaled wave numbers (cm^{-1})	Intensity (km/mol)		Mode	Wave numbers (cm^{-1})	Scaled wave numbers (cm^{-1})	Intensity (km/mol)
(A')	3294.7	3212.3	0.15		(A')	947.2	923.5	13.79
(A')	3294.1	3211.8	0.05		(A')	901.08	878.6	11.5
(A')	3273.1	3191.3	0.24		(A')	900.08	877.6	21.7
(A')	3272.7	3190.9	0.37		(A')	824.38	803.8	12.26
(A')	3262.4	3180.8	2.97		(A')	751.51	732.7	2.31
(A')	3261.4	3179.9	3.82		(A')	646.81	630.6	111.71
(A')	3199	3119.0	4.52		(A')	529.41	516.2	11.83
(A')	3186.8	3107.2	0.68		(A')	438.81	427.8	2.5
(A')	3186	3106.3	0.81		(A')	277.77	270.8	6.01
(A')	3180.8	3101.2	5.15		(A')	251.25	245.0	0.18
(A')	1724.2	1681.1	34.67		(A')	195.53	-	0.05
(A')	1674.9	1633.0	892.59		(A')	113.82	-	0.19
(A')	1643.3	1602.2	182.04		(A')	45.68	-	0.58
(A')	1608.8	1568.6	1.65		(A")	1019.7	994.2	60.89
(A')	1596.4	1556.5	74.44		(A")	1010.6	985.3	0.75
(A')	1520.8	1482.8	20.65		(A")	900.89	878.4	13.81
(A')	1512.4	1474.6	80.09		(A")	892.11	869.8	0.85
(A')	1428.5	1392.8	34.71		(A")	891.92	869.6	1.16
(A')	1427.7	1392.0	12.71		(A")	859.05	837.6	1.78
(A')	1358.7	1324.7	292.19		(A")	831.31	810.5	18.61
(A')	1332.5	1299.2	0.16		(A")	826.31	805.7	0.01
(A')	1315.1	1282.2	20.92		(A")	766.67	747.5	95.78
(A')	1305.6	1273.0	22.45		(A")	764.98	745.9	29.48
(A')	1291.8	1259.5	53.7		(A")	731.66	713.4	4.74
(A')	1275.5	1243.6	10.22		(A")	672.45	655.6	0.01
(A')	1226.1	1195.4	60.93		(A")	639.36	623.4	0.39
(A')	1213.9	1183.5	11.81		(A")	608.81	593.6	1.7
(A')	1191.4	1161.6	0.71		(A")	608.04	592.8	14.74
(A')	1170.1	1140.8	7.32		(A")	342.45	333.9	2.58
(A')	1122.9	1094.9	19.76		(A")	326.31	318.2	4.13
(A')	1112	1084.2	63		(A")	200.02	195.0	0.41
(A')	1105.1	1077.4	290.54		(A")	131.09	-	0.62
(A')	1039.5	1013.6	16.66		(A")	100.35	-	0.28
(A')	1038	1012.1	121.42		(A")	95.1	-	0.82
(A')	984.73	960.1	4.38		(A")	42.02	-	0.69
(A')	957.12	933.2	14.01		(A")	31.78	-	0.47

Table S7. Harmonic Vibrational Frequencies and IR Intensities of FAF13 at the revDSD/junTZ level of theory scaled frequencies based on B3/junDZ anharmonic corrections.

Mode	Wave numbers (cm^{-1})	Scaled wave numbers (cm^{-1})	Intensity (km/mol)		Mode	Wave numbers (cm^{-1})	Intensity (km/mol)	Intensity (km/mol)
(A')	3294.1	3211.75	0.04		(A')	922.91	899.84	10
(A')	3292.9	3210.61	0.02		(A')	900.94	878.42	12.76
(A')	3272.4	3190.58	0.22		(A')	900.09	877.59	16.76
(A')	3271.6	3189.79	0.19		(A')	794.9	775.03	9.31
(A')	3261.1	3179.61	3.16		(A')	757.31	738.38	1.58
(A')	3260.7	3179.22	3		(A')	621.42	605.88	60.31
(A')	3224.7	3144.03	3.77		(A')	535.36	521.98	7.4
(A')	3198.3	3118.33	4.91		(A')	412.63	402.31	14.92
(A')	3189.2	3109.47	0.13		(A')	358.31	349.35	3.11
(A')	3181.4	3101.87	4.89		(A')	241.27	235.24	4.25
(A')	1709	1666.31	298.92		(A')	193.08	188.25	0.65
(A')	1678.4	1636.47	126.43		(A')	112.75	109.93	1.61
(A')	1654.8	1613.47	326.57		(A')	41.94	40.89	0.57
(A')	1610.3	1570.00	17.63		(A")	1016.1	990.67	60.44
(A')	1598.8	1558.83	65.73		(A")	1000.4	975.40	4.47
(A')	1523	1484.94	23.04		(A")	893.01	870.68	5.4
(A')	1514.5	1476.59	88.54		(A")	890.12	867.87	0.23
(A')	1429.8	1394.08	4.72		(A")	888.75	866.53	1.28
(A')	1428	1392.27	20.2		(A")	863.63	842.04	0.18
(A')	1367.4	1333.24	86.42		(A")	830	809.25	18.95
(A')	1334.3	1300.94	165.09		(A")	825.25	804.62	2.99
(A')	1315	1282.13	25.62		(A")	763.53	744.44	99.41
(A')	1302.4	1269.88	2.6		(A")	761.88	742.83	25.01
(A')	1297.6	1265.19	87.33		(A")	725.31	707.18	4.39
(A')	1279.1	1247.16	20.3		(A")	670.31	653.55	0.1
(A')	1246	1214.84	155.08		(A")	627.69	612.00	1.02
(A')	1216.7	1186.29	4.48		(A")	606.3	591.14	5.93
(A')	1196.5	1166.56	153.49		(A")	604.28	589.17	9.69
(A')	1191.5	1161.75	4.26		(A")	340.97	332.45	0.65
(A')	1170.7	1141.38	19.73		(A")	308.23	300.52	5.94
(A')	1117.2	1089.27	17.07		(A")	165.01		0.81
(A')	1110	1082.25	7.72		(A")	122.8		1.49
(A')	1040.1	1014.12	34.41		(A")	96.94		0.15
(A')	1038.3	1012.38	75.84		(A")	89.03		0.54
(A')	968.18	943.98	14.92		(A")	31.84		1.75
(A')	949.16	925.43	9.27		(A")	24.79		0.003

5.2. Combinations bands and Overtones

In the following the combinations and Overtones for the four most stable species of FAF are reported considering only 20 km/mol.

Table S8. Anharmonic Vibrational Frequencies and IR Intensities for combination bands and overtones of FAF12 at the B3LYP/junDZ level of theory.

Mode 1	Mode 2	Harmonic Combination (cm^{-1})	Anharmonic Value (cm^{-1})	Anharmonic Intensity (km/mol)
1417.98	258.12	1676.10	1638.46	221.3
275.93	1322.40	1598.33	1572.10	113.8
1024.86	192.97	1217.83	1201.08	87.2
1011.08	677.63	1688.71	1639.5	78.1
938.56	756.24	1694.80	1666.29	52.7
275.93	940.83	1216.76	1194.36	29.0
912.78	677.63	1590.41	1543.84	20.3

Table S9. Anharmonic Vibrational Frequencies and IR Intensities for combination bands and overtones of FAF29 at the B3LYP/junDZ level of theory.

Mode 1	Mode 2	Harmonic Combination (cm^{-1})	Anharmonic Value (cm^{-1})	Anharmonic Intensity (km/mol)
413.55	901.35	1314.90	1299.37	124.1
623.51	1026.38	1649.89	1632.18	109.8
346.89	1009.56	1356.44	1306.43	106.7
413.55	1231.43	1644.98	1619.83	77.2
153.74	871.65	1025.39	977.65	62.5
111.45	1168.70	1280.14	1242.93	58.7
370.72	1314.38	1685.10	1652.88	45.9
370.72	1301.40	1672.12	1640.06	38.4
799.73	921.85	1721.57	1692.85	36.7
413.55	940.98	1354.53	1331.90	33.8
540.46	1171.68	1712.13	1678.89	31.1
89.19	921.85	1011.03	989.58	29.9
760.87	940.98	1701.84	1671.28	29.8
733.85	909.18	1643.02	1585.53	26.8
413.55	948.63	1362.18	1340.58	24.9
413.55	1273.64	1687.19	1658.33	24.7
540.46	1105.08	1645.54	1620.61	22.0
760.87	948.63	1709.50	1680.51	21.5

Table S10. Anharmonic Vibrational Frequencies and IR Intensities for combination bands and overtones of FAF1 at the B3LYP/junDZ level of theory.

Mode 1	Mode 2	Harmonic Combination (cm^{-1})	Anharmonic Value (cm^{-1})	Anharmonic Intensity (km/mol)
251.72	1420.89	1672.61	1631.48	1111.7
343.50	1011.91	1355.41	1314.76	415.9
96.02	1003.27	1099.28	1063.59	306.3
112.18	984.96	1097.13	1074.79	188.2
753.58	754.40	1507.98	1469.48	181.9
649.57	1026.97	1676.54	1658.31	170.7
196.48	1156.98	1353.46	1329.29	146.8
673.33	1003.27	1676.59	1630.35	110.8
604.11	753.58	1357.69	1326.63	68.8
603.54	753.58	1357.12	1326.81	53.7
649.57	1025.78	1675.35	1656.10	46.3
251.72	1418.11	1669.82	1630.06	38.2
278.45	1355.15	1633.60	1602.03	37.9
673.33	1011.91	1685.23	1638.90	35.1
196.48	1308.72	1505.20	1468.87	34.8
343.50	753.58	1097.08	1067.54	34.2
251.72	1099.95	1351.66	1325.88	32.3
196.48	902.55	1099.03	1082.19	29.6
222.10	908.30	1130.40	1086.67	27.8
649.57	984.96	1634.53	1612.41	25.7
251.72	939.36	1191.07	1168.47	22.9

Table S11. Anharmonic Vibrational Frequencies and IR Intensities for combination bands and overtones of FAF13 at the B3LYP/junDZ level of theory.

Harmonic Mode 1	Harmonic Mode 2	Harmonic Combination (cm^{-1})	Anharmonic Value (cm^{-1})	Anharmonic Intensity (km/mol)
623.88	1025.76	1649.65	1634.40	75.83
604.20	673.83	1278.03	1248.29	70.78
760.98	941.79	1702.77	1674.52	58.80
358.80	1292.94	1651.75	1628.35	55.89
821.86	827.14	1649.00	1597.94	50.06
753.39	892.01	1645.40	1593.44	49.99
753.39	890.79	1644.18	1591.35	40.32
539.53	902.40	1643.51	1620.76	37.52
821.86	821.86	1643.72	1593.96	36.71
314.92	1009.60	1324.52	1278.11	33.66
539.53	1171.35	1710.89	1677.18	31.61
796.63	925.38	1722.01	1694.89	31.53
630.65	729.74	1360.39	1328.29	30.50
113.50	1596.10	1709.60	1664.26	30.46
796.63	796.63	1593.26	1569.16	28.31

752.15	892.01	1644.16	1588.68	27.57
604.20	630.65	1234.85	1208.98	26.63
358.80	925.38	1284.18	1265.34	25.34
415.04	1180.09	1595.14	1559.05	24.04
343.95	890.79	1234.74	1193.33	23.44

Table S12. B3LYP-D3/jun-cc-pVDZ gas-phase and PCM harmonic frequencies (in cm^{-1}) for all 30 FAF conformers together with the solvation induced shifts (Δ in cm^{-1}), $\Delta = \omega_{gas} - \omega_{solvent}$.

FAF1				FAF2		
gas	PCM	Δ		gas	PCM	Δ
28.1	27.3	-0.8		35.5	35.2	-0.3
39.7	41.6	2.0		36.8	38.1	1.4
42.2	42.2	0.0		40.7	43.2	2.6
96.0	95.9	-0.1		84.2	85.8	1.6
99.3	99.9	0.6		111.4	111.1	-0.4
112.2	111.5	-0.7		113.4	111.7	-1.6
141.4	148.0	6.6		143.6	154.4	10.8
196.5	198.2	1.7		161.3	162.8	1.4
222.2	227.6	5.4		176.9	184.3	7.4
251.7	252.0	0.3		275.5	276.3	0.7
278.5	276.3	-2.3		311.3	310.8	-0.4
327.4	326.8	-0.6		344.4	347.0	2.6
343.5	342.7	-0.8		351.3	351.2	-0.1
440.1	438.8	-1.3		399.3	401.8	2.5
532.6	534.5	1.9		541.5	545.0	3.5
603.5	600.0	-3.5		582.9	583.3	0.4
604.1	601.5	-2.6		601.9	600.3	-1.7
640.7	642.1	1.5		605.0	602.1	-2.8
649.6	644.5	-5.1		622.8	626.2	3.4
673.3	670.5	-2.9		671.5	669.4	-2.1
733.1	734.1	1.0		710.8	709.7	-1.1
753.6	755.1	1.5		750.5	757.0	6.5
754.4	760.2	5.8		750.8	757.0	6.2
755.5	762.8	7.3		771.2	772.5	1.2
821.2	823.4	2.2		780.0	781.4	1.3
823.4	832.9	9.4		823.7	833.8	10.1
826.6	839.6	13.0		825.7	836.3	10.7
863.6	868.4	4.8		873.7	874.2	0.5
890.1	897.5	7.4		890.1	893.9	3.8
890.9	898.5	7.6		890.7	897.3	6.6
901.5	901.3	-0.2		895.3	899.3	4.0
902.5	903.6	1.1		897.3	901.1	3.8
908.3	908.6	0.3		902.9	901.6	-1.3
939.4	936.5	-2.9		903.9	902.8	-1.0
953.1	951.0	-2.1		959.8	956.0	-3.8
985.0	988.8	3.8		968.4	965.8	-2.6
1003.3	998.4	-5.0		1002.5	999.6	-2.8
1012.0	1007.0	-4.9		1009.0	1007.0	-2.1
1025.8	1021.3	-4.5		1026.3	1021.2	-5.1

1027.0	1023.2	-3.7		1027.4	1022.6	-4.8
1099.9	1094.9	-5.1		1113.6	1107.3	-6.3
1108.9	1105.9	-3.0		1115.0	1108.7	-6.3
1122.4	1125.9	3.4		1154.1	1149.4	-4.7
1157.0	1150.1	-6.9		1159.8	1153.5	-6.3
1172.0	1164.2	-7.7		1185.4	1180.0	-5.4
1191.6	1185.0	-6.6		1198.4	1194.6	-3.8
1213.5	1213.8	0.3		1259.4	1261.5	2.1
1258.3	1259.3	0.9		1261.8	1263.9	2.1
1272.5	1270.1	-2.5		1282.2	1282.0	-0.3
1297.6	1294.3	-3.2		1295.2	1296.4	1.3
1308.7	1310.0	1.3		1311.5	1315.6	4.2
1321.8	1319.8	-1.9		1321.1	1322.6	1.6
1355.1	1355.5	0.4		1330.3	1330.8	0.5
1418.1	1414.8	-3.3		1418.8	1415.8	-3.0
1420.9	1418.2	-2.7		1418.9	1416.4	-2.6
1507.7	1499.7	-8.0		1510.2	1499.8	-10.4
1515.4	1509.8	-5.6		1510.6	1503.2	-7.4
1593.9	1576.3	-17.6		1608.7	1589.8	-18.9
1608.4	1596.1	-12.3		1611.8	1608.0	-3.8
1633.3	1605.9	-27.4		1672.0	1623.6	-48.4
1673.2	1650.9	-22.2		1672.5	1657.1	-15.4
1722.6	1700.6	-22.1		1683.9	1670.9	-13.0
3167.9	3173.8	5.9		3171.9	3170.5	-1.4
3176.8	3181.8	5.1		3173.5	3172.2	-1.3
3177.5	3183.4	6.0		3186.1	3188.5	2.4
3188.1	3194.6	6.4		3191.4	3193.6	2.3
3250.4	3252.9	2.4		3251.4	3252.7	1.4
3251.0	3253.6	2.6		3251.4	3252.7	1.3
3262.2	3263.7	1.5		3263.6	3263.6	0.0
3262.6	3264.0	1.4		3263.6	3263.6	0.0
3283.8	3284.6	0.8		3285.7	3285.7	-0.1
3285.1	3285.6	0.5		3285.7	3285.7	-0.1
FAF3				FAF4		
gas	PCM	Δ		gas	PCM	Δ
4.6	23.5	18.9		23.1	22.8	-0.3
32.1	35.6	3.6		39.7	39.5	-0.2
44.1	48.8	4.7		54.0	52.4	-1.6
63.6	70.6	7.0		84.8	79.0	-5.7
96.8	97.2	0.5		107.0	103.2	-3.8
119.3	117.6	-1.8		135.2	132.2	-3.0
146.6	151.2	4.6		152.2	155.4	3.1
194.9	200.2	5.3		220.2	218.5	-1.7
215.1	218.2	3.1		220.8	221.2	0.4

244.5	244.5	0.0		241.1	240.6	-0.5
276.0	274.8	-1.3		282.8	279.9	-2.9
324.7	323.6	-1.1		326.8	324.7	-2.2
427.7	430.8	3.1		422.9	421.5	-1.4
463.7	463.4	-0.3		462.4	461.0	-1.4
557.2	557.7	0.5		561.1	559.8	-1.2
604.0	600.7	-3.3		603.9	600.9	-3.0
604.5	601.2	-3.3		606.4	603.7	-2.7
634.6	637.2	2.6		632.3	633.7	1.4
656.4	653.8	-2.5		659.5	656.9	-2.6
667.6	666.1	-1.6		664.5	662.6	-1.9
733.9	733.5	-0.3		737.0	735.4	-1.5
742.4	744.9	2.5		746.2	747.5	1.3
751.9	758.0	6.1		753.6	759.5	5.9
763.7	765.4	1.7		767.6	771.1	3.5
793.1	801.4	8.3		801.9	807.4	5.5
823.5	832.1	8.6		825.8	828.5	2.8
832.9	836.4	3.5		829.6	837.4	7.8
838.3	848.8	10.5		859.7	863.0	3.3
887.8	892.5	4.7		889.3	893.9	4.5
889.6	898.5	8.9		892.4	897.3	4.9
891.6	899.5	7.8		902.0	898.7	-3.3
902.5	901.2	-1.2		902.8	902.4	-0.4
904.8	904.0	-0.8		923.8	922.2	-1.6
933.4	929.9	-3.4		928.7	922.6	-6.1
948.3	944.7	-3.7		958.1	954.8	-3.3
974.6	975.0	0.4		986.7	986.1	-0.6
995.7	1000.8	5.1		1001.2	1000.8	-0.4
1008.8	1006.7	-2.1		1009.3	1008.7	-0.6
1025.9	1022.5	-3.4		1023.5	1012.2	-11.2
1030.4	1027.1	-3.3		1026.7	1023.3	-3.4
1095.8	1093.4	-2.4		1109.5	1103.2	-6.3
1112.2	1108.3	-3.9		1114.9	1110.7	-4.2
1121.6	1123.3	1.8		1126.5	1127.7	1.2
1155.8	1148.9	-6.8		1155.7	1147.8	-7.9
1177.5	1165.5	-12.0		1164.3	1154.7	-9.6
1190.7	1184.9	-5.7		1184.6	1179.4	-5.3
1223.5	1221.1	-2.4		1191.2	1185.0	-6.2
1243.4	1244.5	1.1		1236.0	1235.5	-0.5
1257.7	1259.0	1.4		1258.2	1259.3	1.1
1311.4	1307.4	-4.0		1282.2	1282.1	-0.1
1333.6	1340.3	6.7		1312.4	1308.3	-4.1
1338.6	1342.0	3.4		1337.1	1340.9	3.7
1417.7	1415.7	-2.0		1410.7	1409.1	-1.7

1424.4	1422.9	-1.5		1419.7	1416.7	-3.1
1430.3	1425.1	-5.2		1453.8	1448.9	-4.9
1507.8	1500.6	-7.2		1508.3	1501.0	-7.3
1513.2	1513.0	-0.2		1515.6	1510.4	-5.2
1576.8	1571.7	-5.1		1600.0	1577.4	-22.6
1608.3	1599.7	-8.7		1606.7	1599.3	-7.4
1640.4	1607.0	-33.4		1616.7	1607.1	-9.6
1668.1	1647.2	-20.9		1657.6	1636.7	-20.9
1727.2	1703.2	-24.0		1712.8	1694.3	-18.4
3144.6	3156.7	12.1		3153.3	3158.9	5.6
3163.8	3171.2	7.4		3169.1	3172.3	3.2
3172.1	3176.1	4.1		3176.0	3180.8	4.8
3177.2	3186.4	9.2		3181.3	3189.8	8.4
3246.9	3250.9	4.0		3250.4	3252.4	2.0
3250.2	3252.6	2.5		3252.1	3255.1	3.0
3259.7	3262.0	2.3		3262.6	3263.4	0.7
3262.5	3263.6	1.1		3278.0	3280.5	2.6
3282.8	3282.3	-0.5		3285.1	3285.5	0.4
3285.0	3285.2	0.2		3300.6	3302.8	2.2
FAF5				FAF6		
gas	PCM	Δ		gas	PCM	Δ
14.5	10.5	-4.0		7.6	25.4	17.8
17.9	19.6	1.7		34.8	36.5	1.7
36.5	38.5	2.0		53.2	54.2	1.1
57.7	58.0	0.3		61.3	70.9	9.6
117.2	113.2	-4.0		96.4	95.1	-1.4
127.4	130.1	2.8		111.0	111.2	0.2
160.0	158.6	-1.4		158.3	159.1	0.8
199.9	201.0	1.1		196.9	202.6	5.7
202.8	202.2	-0.6		218.7	221.1	2.4
248.8	249.4	0.7		251.4	251.2	-0.2
273.0	275.0	2.0		273.6	271.9	-1.7
389.4	392.7	3.3		342.3	341.2	-1.1
435.4	436.3	0.9		431.9	435.2	3.3
494.0	492.5	-1.5		468.7	467.0	-1.6
600.4	598.9	-1.4		560.5	560.4	0.0
603.4	600.4	-3.0		604.2	600.9	-3.2
608.2	608.3	0.1		605.2	601.3	-3.9
633.3	630.5	-2.8		641.1	642.9	1.8
658.1	659.1	1.0		655.1	652.2	-3.0
660.8	661.0	0.2		675.6	673.0	-2.6
691.6	689.1	-2.4		736.7	735.8	-0.9
739.3	740.5	1.2		746.0	748.9	2.9
756.0	759.0	3.0		752.5	760.2	7.6

757.1	759.6	2.5		764.8	767.5	2.7
757.9	759.6	1.7		795.9	803.4	7.5
819.1	823.4	4.3		826.9	835.3	8.3
825.8	827.3	1.5		836.3	838.3	2.0
832.2	841.5	9.3		839.9	851.0	11.1
843.5	848.4	4.9		887.4	894.2	6.8
886.0	898.9	12.9		891.4	897.5	6.0
886.4	899.3	12.9		900.4	899.6	-0.7
904.5	899.6	-4.8		901.5	903.1	1.6
905.0	900.0	-5.1		904.7	905.6	0.9
940.4	936.6	-3.8		936.3	931.4	-4.9
942.3	936.6	-5.7		940.1	936.6	-3.6
955.0	953.7	-1.3		958.6	960.5	1.9
984.3	989.8	5.5		998.9	999.5	0.7
991.6	997.4	5.7		1008.0	1009.3	1.2
1027.6	1023.5	-4.1		1025.3	1020.6	-4.6
1029.1	1025.4	-3.7		1030.7	1027.5	-3.2
1088.2	1087.5	-0.7		1093.5	1088.8	-4.7
1109.0	1103.7	-5.3		1106.2	1102.8	-3.4
1114.1	1115.2	1.1		1115.6	1118.7	3.1
1172.8	1161.5	-11.3		1171.8	1163.1	-8.7
1176.9	1164.5	-12.4		1177.8	1166.0	-11.8
1206.9	1203.0	-3.9		1212.2	1210.9	-1.2
1222.6	1220.0	-2.6		1224.9	1222.5	-2.4
1233.9	1233.4	-0.5		1245.6	1245.9	0.3
1244.9	1244.9	0.0		1272.2	1270.1	-2.2
1325.6	1324.1	-1.4		1306.3	1304.5	-1.8
1327.7	1326.4	-1.3		1323.0	1323.0	0.0
1415.0	1415.8	0.8		1341.8	1344.1	2.3
1418.4	1417.0	-1.4		1417.5	1413.6	-3.9
1426.8	1420.6	-6.2		1423.7	1422.8	-0.9
1427.9	1421.1	-6.7		1430.9	1425.5	-5.5
1508.9	1507.0	-1.9		1509.6	1505.8	-3.7
1516.0	1515.2	-0.7		1517.7	1515.4	-2.3
1579.7	1576.4	-3.3		1575.3	1569.7	-5.6
1583.4	1580.6	-2.8		1594.3	1585.4	-8.9
1657.1	1626.7	-30.4		1641.8	1605.6	-36.2
1675.1	1658.9	-16.3		1673.0	1651.6	-21.4
1733.5	1708.7	-24.8		1729.4	1707.2	-22.1
3143.8	3153.7	9.8		3144.9	3154.7	9.8
3149.1	3158.6	9.5		3168.7	3176.7	8.0
3164.8	3172.1	7.3		3174.8	3178.5	3.7
3172.8	3180.5	7.7		3190.0	3195.1	5.0
3247.2	3250.4	3.2		3246.8	3250.8	4.1

3247.2	3250.4	3.2		3250.4	3252.8	2.3
3259.8	3261.6	1.8		3259.5	3262.0	2.5
3259.8	3261.6	1.8		3261.7	3263.3	1.6
3283.7	3282.7	-1.0		3282.3	3282.0	-0.2
3283.8	3282.7	-1.0		3283.7	3284.4	0.7
FAF7				FAF8		
gas	PCM	Δ		gas	PCM	Δ
26.7	28.1	1.4		14.6	16.9	2.2
36.1	42.4	6.3		27.5	27.1	-0.4
43.0	43.9	0.9		43.7	45.3	1.6
88.7	93.5	4.7		75.1	72.5	-2.5
101.6	102.0	0.4		96.8	93.0	-3.8
115.1	113.1	-2.0		145.8	146.5	0.6
140.4	148.1	7.7		177.5	176.7	-0.8
197.9	199.0	1.0		211.0	211.8	0.8
213.3	218.2	4.9		225.4	223.9	-1.4
247.7	247.0	-0.7		253.9	255.6	1.7
280.6	279.3	-1.3		260.1	257.8	-2.3
326.9	325.6	-1.3		381.8	382.6	0.8
326.9	326.7	-0.3		456.4	459.5	3.2
435.9	435.0	-0.9		482.0	480.1	-1.9
530.9	533.9	3.0		603.6	600.7	-2.9
603.3	601.1	-2.2		606.5	604.0	-2.5
604.4	601.6	-2.8		625.9	629.6	3.6
636.5	638.6	2.1		637.7	633.8	-3.9
647.5	642.5	-5.0		652.9	656.0	3.1
667.7	665.7	-2.0		664.8	664.9	0.0
730.5	731.5	1.0		686.5	684.1	-2.4
753.5	755.5	2.0		746.1	748.6	2.6
754.6	760.5	5.9		759.2	762.6	3.5
754.9	760.9	6.0		765.0	769.6	4.6
821.0	820.9	-0.1		776.0	781.9	5.9
821.4	833.8	12.4		823.0	826.5	3.4
825.3	839.1	13.8		831.4	841.0	9.6
857.7	863.6	5.9		855.5	857.0	1.5
890.1	897.9	7.9		860.1	863.0	2.9
890.2	898.7	8.5		888.3	897.7	9.3
902.3	902.1	-0.1		902.6	899.7	-2.8
902.7	903.7	1.1		904.7	901.5	-3.2
902.8	906.9	4.1		924.6	921.7	-2.9
946.9	944.4	-2.4		926.2	923.7	-2.5
961.6	958.9	-2.6		942.7	936.6	-6.1
989.7	993.2	3.5		971.6	970.6	-1.0
1004.2	998.9	-5.4		990.5	998.9	8.5

1012.7	1007.4	-5.3		1003.1	1007.6	4.5
1025.8	1020.8	-5.0		1023.0	1012.1	-11.0
1027.7	1024.2	-3.5		1029.4	1025.8	-3.7
1102.4	1097.6	-4.8		1101.6	1095.2	-6.4
1117.4	1111.7	-5.8		1114.2	1109.5	-4.7
1125.6	1129.9	4.3		1119.4	1119.1	-0.3
1156.5	1148.9	-7.5		1162.7	1152.2	-10.5
1157.7	1152.0	-5.7		1176.0	1164.9	-11.0
1191.3	1183.8	-7.5		1184.2	1178.5	-5.7
1192.3	1186.4	-5.9		1217.4	1216.9	-0.5
1258.1	1259.6	1.5		1232.6	1232.0	-0.6
1259.0	1260.2	1.3		1245.1	1247.2	2.1
1298.5	1294.2	-4.4		1284.8	1285.8	1.0
1315.5	1314.9	-0.7		1332.5	1336.3	3.8
1321.5	1325.1	3.6		1409.3	1407.8	-1.5
1362.9	1364.1	1.2		1418.8	1420.8	2.0
1419.2	1416.8	-2.3		1428.4	1423.1	-5.3
1422.9	1420.7	-2.2		1450.7	1446.9	-3.8
1507.7	1499.5	-8.2		1510.3	1506.7	-3.7
1508.0	1501.1	-7.0		1517.5	1514.8	-2.7
1606.8	1581.4	-25.4		1579.1	1573.6	-5.5
1609.1	1604.9	-4.2		1601.6	1592.8	-8.7
1631.4	1606.8	-24.6		1623.6	1601.7	-21.9
1667.5	1645.5	-22.1		1663.0	1641.3	-21.6
1720.0	1696.0	-24.0		1720.1	1701.4	-18.7
3163.0	3172.1	9.1		3145.9	3155.2	9.3
3171.3	3174.3	2.9		3153.0	3157.4	4.4
3176.8	3178.1	1.3		3168.1	3176.3	8.3
3178.9	3186.6	7.6		3179.9	3188.2	8.3
3250.5	3253.4	2.9		3247.1	3250.3	3.1
3250.5	3253.4	2.9		3251.3	3254.5	3.2
3262.7	3264.3	1.6		3259.7	3261.5	1.8
3262.7	3264.3	1.6		3277.8	3280.3	2.5
3285.3	3285.7	0.4		3282.9	3282.2	-0.7
3285.3	3285.7	0.4		3301.4	3304.6	3.2
FAF9				FAF10		
gas	PCM	Δ		gas	PCM	Δ
22.7	23.8	1.1		28.1	28.5	0.4
46.1	44.0	-2.1		39.5	38.0	-1.6
54.5	54.0	-0.6		62.9	61.8	-1.1
83.8	81.3	-2.5		71.3	70.2	-1.1
107.6	103.3	-4.3		108.9	95.6	-13.3
127.5	125.9	-1.7		170.9	168.2	-2.7
163.5	164.8	1.3		184.3	184.9	0.5

219.7	218.2	-1.5		221.7	221.8	0.0
226.1	228.0	1.9		235.2	232.4	-2.7
250.6	250.6	0.0		251.1	250.8	-0.3
278.3	274.9	-3.4		259.2	257.9	-1.3
344.4	343.8	-0.5		380.2	379.5	-0.7
423.5	422.7	-0.9		468.6	469.3	0.6
468.1	466.6	-1.5		476.1	474.4	-1.7
563.1	562.0	-1.1		606.4	603.8	-2.6
604.0	600.5	-3.5		607.3	604.3	-3.0
606.4	603.6	-2.8		631.4	632.7	1.3
634.3	635.5	1.2		639.1	636.0	-3.1
659.2	657.2	-2.0		655.7	656.5	0.9
673.3	670.6	-2.8		664.7	664.0	-0.7
738.1	736.1	-2.0		684.0	683.3	-0.7
749.0	749.9	0.9		756.8	757.7	0.9
753.9	761.4	7.5		765.0	769.8	4.8
767.1	771.4	4.3		767.2	770.4	3.1
802.8	808.2	5.4		783.1	786.5	3.4
829.9	831.5	1.6		831.5	829.9	-1.6
832.1	839.9	7.9		852.3	854.5	2.2
859.3	863.7	4.5		857.2	863.1	5.9
888.5	894.7	6.2		861.3	864.9	3.6
901.2	897.6	-3.7		902.2	897.6	-4.6
901.5	897.7	-3.8		902.8	897.9	-4.9
902.4	903.9	1.5		917.7	916.2	-1.5
927.4	923.5	-4.0		925.4	924.8	-0.6
927.9	926.6	-1.2		928.3	925.1	-3.2
939.9	936.5	-3.5		945.2	942.8	-2.4
981.0	980.7	-0.2		985.1	983.3	-1.8
1001.5	1000.3	-1.2		999.2	1003.7	4.6
1008.8	1008.7	0.0		1006.8	1010.6	3.8
1022.7	1012.8	-9.9		1019.6	1011.0	-8.6
1026.2	1021.9	-4.4		1024.1	1014.8	-9.3
1101.8	1095.5	-6.3		1112.7	1106.6	-6.1
1114.5	1110.0	-4.4		1115.5	1111.5	-4.0
1120.4	1122.7	2.3		1125.6	1123.0	-2.6
1163.4	1152.9	-10.5		1162.0	1150.5	-11.5
1172.1	1164.0	-8.0		1165.1	1156.0	-9.1
1184.6	1179.5	-5.1		1184.2	1178.9	-5.3
1214.0	1213.0	-0.9		1185.1	1179.1	-6.0
1236.0	1235.8	-0.2		1229.0	1227.6	-1.4
1273.7	1270.7	-3.0		1243.1	1242.2	-0.8
1282.3	1282.9	0.6		1279.9	1278.8	-1.0
1308.0	1306.6	-1.4		1292.8	1293.1	0.4

1325.1	1326.2	1.1		1410.0	1408.4	-1.6
1410.7	1409.1	-1.6		1410.2	1408.4	-1.8
1417.9	1413.9	-4.0		1446.1	1444.3	-1.8
1453.1	1448.4	-4.7		1458.6	1452.3	-6.3
1512.5	1506.1	-6.4		1513.8	1508.1	-5.7
1518.3	1513.5	-4.8		1517.3	1513.0	-4.3
1593.0	1574.5	-18.5		1595.9	1576.8	-19.1
1600.0	1591.6	-8.5		1605.7	1597.4	-8.3
1617.4	1602.2	-15.2		1607.5	1601.8	-5.8
1663.8	1642.4	-21.4		1644.1	1626.4	-17.7
1716.4	1699.4	-16.9		1706.6	1691.8	-14.8
3154.1	3159.2	5.1		3152.8	3157.7	4.9
3175.6	3178.8	3.2		3155.6	3159.4	3.8
3177.3	3184.1	6.8		3174.7	3180.5	5.7
3192.8	3196.3	3.4		3186.2	3192.5	6.3
3250.8	3253.0	2.2		3251.7	3254.7	3.0
3251.9	3255.1	3.1		3251.8	3254.7	2.9
3262.0	3263.6	1.5		3278.4	3280.5	2.2
3277.9	3280.3	2.4		3278.4	3280.5	2.2
3283.8	3284.5	0.7		3305.2	3305.0	-0.3
3300.8	3302.7	1.9		3308.0	3305.6	-2.4
FAF11				FAF12		
gas	PCM	Δ		gas	PCM	Δ
26.3	31.0	4.6		30.6	30.7	0.1
35.1	36.7	1.6		41.4	41.8	0.4
44.5	43.8	-0.7		43.5	43.2	-0.3
82.6	84.4	1.8		95.2	94.8	-0.3
107.0	108.0	1.1		103.2	101.8	-1.3
114.1	113.5	-0.5		110.5	109.6	-1.0
138.7	145.1	6.5		147.4	149.8	2.4
180.2	180.9	0.7		193.0	195.1	2.1
189.7	194.6	4.9		231.4	231.2	-0.1
249.2	248.8	-0.4		258.1	258.2	0.1
314.2	315.2	1.0		275.9	274.3	-1.7
340.8	342.4	1.6		342.6	342.6	0.0
370.4	370.8	0.4		345.4	346.4	1.1
407.0	407.1	0.2		444.2	441.4	-2.8
537.8	540.3	2.5		534.4	535.4	1.0
603.6	600.3	-3.4		603.9	600.3	-3.5
604.1	601.3	-2.8		603.9	600.4	-3.5
620.7	618.2	-2.5		647.0	646.4	-0.6
629.9	632.4	2.5		651.6	647.4	-4.2
673.7	670.4	-3.3		677.6	673.9	-3.7
731.7	733.4	1.7		735.7	736.9	1.2

752.7	759.0	6.4		754.2	755.4	1.2
755.1	760.5	5.4		754.4	762.7	8.3
759.9	762.6	2.7		756.2	762.8	6.5
799.3	798.4	-0.9		820.2	825.5	5.3
818.7	832.8	14.1		826.1	832.0	5.9
823.9	837.9	14.0		828.2	841.1	12.9
863.6	868.5	4.9		869.2	869.2	0.1
888.3	897.9	9.5		889.7	897.3	7.6
888.6	898.8	10.1		891.9	897.8	6.0
902.0	900.8	-1.2		901.5	901.0	-0.5
902.6	901.3	-1.3		901.7	903.7	2.0
907.3	911.0	3.7		912.8	909.4	-3.4
916.4	921.5	5.1		938.6	935.2	-3.4
947.2	942.6	-4.7		940.8	937.0	-3.8
967.7	966.7	-1.0		977.9	983.7	5.8
995.9	990.7	-5.2		1003.0	998.4	-4.6
1010.9	1006.1	-4.8		1011.1	1006.5	-4.6
1026.0	1022.0	-4.1		1024.9	1019.8	-5.1
1026.9	1023.0	-3.9		1026.9	1022.7	-4.2
1102.7	1096.6	-6.1		1098.1	1092.2	-6.0
1115.7	1108.8	-6.9		1105.6	1101.7	-4.0
1155.0	1149.6	-5.4		1113.9	1119.4	5.6
1170.8	1162.5	-8.3		1171.7	1163.2	-8.5
1189.5	1185.0	-4.5		1172.6	1164.6	-8.0
1203.7	1204.5	0.8		1208.7	1209.3	0.5
1222.5	1224.6	2.2		1217.6	1217.4	-0.2
1258.9	1260.3	1.4		1268.2	1267.3	-1.0
1273.7	1276.5	2.9		1277.5	1272.4	-5.0
1301.1	1300.5	-0.6		1297.1	1294.6	-2.5
1306.4	1308.1	1.8		1304.4	1307.2	2.8
1321.8	1318.7	-3.1		1322.4	1318.4	-4.0
1366.5	1370.3	3.8		1343.6	1342.8	-0.8
1420.0	1415.7	-4.3		1418.0	1414.2	-3.8
1421.0	1418.7	-2.3		1418.3	1414.7	-3.6
1508.3	1499.5	-8.8		1512.6	1505.6	-6.9
1513.8	1507.2	-6.6		1517.6	1512.3	-5.3
1592.4	1577.7	-14.8		1591.4	1572.8	-18.6
1609.1	1601.2	-7.9		1596.9	1590.4	-6.6
1644.2	1608.7	-35.5		1635.6	1599.6	-36.0
1686.8	1667.6	-19.2		1678.7	1657.1	-21.6
1704.3	1681.1	-23.1		1724.9	1704.6	-20.3
3177.8	3177.6	-0.3		3176.0	3179.5	3.5
3181.9	3182.8	1.0		3177.1	3180.7	3.6
3191.7	3196.8	5.1		3184.0	3187.6	3.6

3199.7	3211.8	12.1		3192.7	3196.7	4.0
3249.7	3252.3	2.6		3250.8	3253.3	2.4
3250.0	3253.0	3.0		3250.9	3253.3	2.4
3261.6	3263.3	1.7		3262.1	3263.8	1.8
3262.1	3263.9	1.7		3262.1	3263.9	1.8
3285.2	3285.1	-0.1		3283.8	3284.7	1.0
3285.3	3285.9	0.6		3283.8	3284.7	0.9
FAF13				FAF14		
gas	PCM	Δ		gas	pcm	Δ
GAS frequency (cm^{-1})	PCM frequency (cm^{-1})	Δ (cm^{-1})		GAS frequency (cm^{-1})	PCM frequency (cm^{-1})	Δ (cm^{-1})
31.1	31.3	0.2		36.5	35.6	-0.9
35.2	36.1	0.9		37.1	37.6	0.5
41.8	42.1	0.3		45.8	47.8	2.0
92.2	92.6	0.4		82.8	83.4	0.6
100.2	100.2	0.0		110.8	110.6	-0.2
113.5	113.2	-0.3		113.2	113.0	-0.2
143.7	154.9	11.2		150.1	157.6	7.5
182.1	185.8	3.7		157.9	159.0	1.1
193.9	194.9	1.0		186.5	189.3	2.8
241.5	239.9	-1.6		279.2	280.6	1.4
314.9	314.4	-0.5		316.5	316.8	0.3
343.9	343.7	-0.2		350.5	351.1	0.7
358.8	358.6	-0.2		356.2	358.3	2.1
415.0	414.0	-1.0		404.9	406.1	1.1
539.5	541.4	1.8		543.2	545.9	2.7
603.1	600.4	-2.8		583.3	583.9	0.6
604.2	601.1	-3.1		603.6	601.0	-2.5
623.9	621.1	-2.8		604.2	601.1	-3.1
630.6	633.2	2.6		624.5	627.5	3.0
673.8	670.6	-3.3		677.2	673.6	-3.6
729.7	731.3	1.6		714.6	713.6	-1.0
752.2	760.6	8.4		750.2	757.0	6.8
753.4	760.8	7.4		753.4	759.5	6.1
761.0	760.8	-0.1		772.3	772.9	0.7
796.6	795.8	-0.8		780.0	781.0	1.0
821.9	832.6	10.7		821.0	832.8	11.7
827.1	838.9	11.8		825.4	836.4	11.0
870.3	871.7	1.4		876.6	875.6	-1.0
890.8	897.8	7.0		888.3	893.7	5.4
892.0	898.4	6.4		890.6	897.7	7.0
901.5	901.4	-0.1		896.4	898.8	2.4
902.4	902.7	0.3		899.8	900.9	1.1
905.5	907.6	2.1		902.4	901.8	-0.7

925.4	931.2	5.8		903.8	905.0	1.2
941.8	940.1	-1.7		945.3	940.4	-4.8
961.8	958.2	-3.6		963.9	961.0	-2.9
994.9	991.0	-3.9		1001.9	998.7	-3.2
1009.6	1004.4	-5.2		1008.7	1006.6	-2.1
1025.8	1022.1	-3.7		1025.6	1020.6	-5.0
1027.3	1023.4	-3.9		1026.7	1022.5	-4.2
1104.0	1099.3	-4.6		1103.1	1097.9	-5.1
1114.5	1109.3	-5.2		1114.1	1108.6	-5.4
1157.1	1152.7	-4.4		1156.7	1151.7	-5.1
1171.4	1163.2	-8.2		1171.8	1163.7	-8.1
1180.1	1176.2	-3.9		1189.9	1186.4	-3.5
1199.9	1199.7	-0.2		1210.6	1210.5	-0.1
1233.1	1235.1	2.0		1259.9	1262.0	2.1
1262.5	1264.3	1.7		1270.3	1271.5	1.2
1279.2	1274.7	-4.5		1287.4	1288.3	0.9
1292.9	1299.0	6.1		1302.0	1302.1	0.2
1307.2	1305.9	-1.3		1304.0	1303.8	-0.2
1327.4	1326.4	-1.0		1312.5	1316.5	4.0
1357.8	1357.7	-0.1		1326.4	1327.2	0.8
1417.7	1414.2	-3.5		1418.4	1414.3	-4.1
1419.8	1417.6	-2.2		1418.9	1416.0	-2.9
1509.0	1498.3	-10.7		1510.5	1500.2	-10.2
1516.9	1511.7	-5.2		1514.5	1508.3	-6.3
1596.1	1579.4	-16.7		1593.7	1583.4	-10.3
1608.5	1595.2	-13.3		1610.6	1600.3	-10.3
1647.1	1615.7	-31.4		1673.2	1620.6	-52.6
1678.0	1661.0	-16.9		1678.2	1661.4	-16.8
1708.7	1686.4	-22.3		1689.8	1678.9	-10.8
3174.6	3173.2	-1.3		3172.2	3170.9	-1.3
3180.6	3184.0	3.4		3182.8	3180.1	-2.7
3188.5	3192.1	3.5		3188.1	3189.9	1.8
3212.4	3216.8	4.3		3192.6	3197.0	4.4
3250.7	3253.2	2.4		3249.5	3252.1	2.5
3251.9	3253.7	1.8		3251.3	3252.8	1.5
3262.0	3263.7	1.7		3261.2	3263.0	1.8
3264.2	3264.5	0.3		3263.6	3263.7	0.1
3283.9	3284.7	0.9		3285.1	3285.2	0.1
3285.6	3285.9	0.3		3285.6	3285.7	0.0
FAF15				FAF16		
gas	PCM	Δ		gas	pcm	Δ
27.4	15.8	-11.6		21.8	20.4	-1.4
33.9	29.7	-4.2		35.5	35.1	-0.5
42.8	42.0	-0.9		39.2	40.1	0.9

85.3	88.3	3.1		88.9	78.5	-10.4
105.8	104.0	-1.8		112.9	113.3	0.4
116.2	111.6	-4.6		127.1	125.0	-2.1
134.5	127.7	-6.8		171.0	168.7	-2.4
170.7	174.9	4.2		203.2	202.0	-1.1
187.7	186.2	-1.6		222.9	223.2	0.3
248.8	248.5	-0.3		234.5	233.9	-0.6
307.1	299.8	-7.4		326.3	329.0	2.6
333.9	331.5	-2.4		395.0	393.1	-1.9
358.5	358.5	0.0		436.6	437.3	0.8
408.8	407.0	-1.9		482.5	478.1	-4.5
537.1	537.9	0.8		590.5	595.6	5.1
601.8	599.4	-2.4		604.3	601.1	-3.2
604.7	602.0	-2.7		607.3	604.8	-2.4
621.2	616.9	-4.3		639.2	638.9	-0.3
627.3	628.3	1.1		653.1	652.5	-0.6
667.8	665.1	-2.7		664.5	663.1	-1.4
727.1	728.3	1.2		698.0	699.0	0.9
752.0	758.7	6.7		731.2	730.8	-0.4
753.4	760.4	7.1		751.1	755.2	4.1
760.1	760.7	0.6		764.9	769.6	4.6
796.5	795.5	-0.9		775.2	774.8	-0.4
822.2	834.4	12.2		792.2	793.2	1.0
826.4	838.2	11.8		826.1	836.3	10.2
862.4	866.7	4.3		856.8	859.3	2.5
889.4	898.5	9.1		866.1	864.7	-1.4
891.9	898.8	6.9		886.8	894.9	8.2
902.3	900.3	-2.0		897.7	897.8	0.1
902.6	902.1	-0.6		904.1	900.3	-3.8
903.6	905.0	1.3		905.6	903.5	-2.1
919.6	923.4	3.8		924.4	919.9	-4.5
960.0	957.3	-2.8		940.4	936.5	-3.9
969.0	966.6	-2.4		960.5	958.7	-1.8
995.4	985.3	-10.1		984.4	986.0	1.6
1011.5	1005.4	-6.2		1007.7	1011.5	3.9
1026.4	1021.8	-4.6		1024.5	1014.5	-10.0
1027.8	1023.8	-4.0		1026.2	1022.2	-3.9
1114.2	1107.7	-6.5		1105.0	1101.1	-3.9
1115.7	1109.4	-6.3		1114.9	1110.2	-4.7
1154.7	1148.2	-6.5		1138.3	1137.4	-0.9
1157.9	1153.3	-4.7		1162.6	1153.6	-9.0
1183.3	1178.0	-5.3		1170.6	1163.5	-7.1
1191.2	1185.1	-6.1		1184.2	1179.0	-5.2
1224.6	1225.7	1.2		1228.4	1228.8	0.4

1259.4	1260.6	1.3		1236.3	1235.4	-0.9
1262.8	1264.7	1.9		1240.0	1241.3	1.3
1292.8	1292.7	-0.2		1284.3	1284.3	0.0
1310.8	1307.8	-3.0		1311.2	1312.5	1.3
1331.1	1327.9	-3.2		1396.1	1397.3	1.2
1367.5	1369.2	1.7		1409.9	1408.4	-1.5
1419.4	1417.2	-2.1		1419.3	1415.3	-3.9
1421.5	1418.8	-2.7		1453.6	1449.3	-4.3
1508.7	1498.0	-10.7		1512.0	1505.1	-7.0
1509.7	1503.0	-6.7		1518.4	1514.2	-4.2
1608.8	1583.4	-25.4		1586.8	1576.1	-10.7
1609.1	1606.6	-2.6		1602.6	1590.9	-11.7
1642.9	1613.4	-29.5		1620.9	1600.8	-20.1
1678.1	1660.5	-17.6		1687.9	1671.1	-16.8
1703.2	1678.1	-25.1		1700.8	1680.8	-20.0
3174.8	3172.3	-2.5		3154.4	3158.0	3.5
3178.8	3177.6	-1.2		3156.1	3160.1	4.0
3185.0	3186.8	1.8		3177.3	3175.2	-2.1
3199.9	3209.3	9.4		3199.9	3207.3	7.4
3250.2	3252.5	2.4		3249.0	3251.0	2.0
3252.0	3254.3	2.3		3251.8	3254.8	3.1
3262.2	3263.5	1.3		3260.8	3262.1	1.2
3264.2	3265.3	1.1		3278.1	3280.7	2.6
3285.3	3285.7	0.4		3285.2	3284.8	-0.4
3285.8	3286.0	0.2		3303.6	3304.8	1.3
FAF17				FAF18		
gas	PCM	Δ		gas	pcm	Δ
26.9	26.6	-0.2		24.4	23.4	-1.0
34.1	32.6	-1.5		39.9	38.5	-1.4
40.0	40.7	0.8		49.3	49.8	0.5
76.0	76.9	0.8		73.0	73.2	0.3
87.5	88.0	0.5		91.1	90.6	-0.5
106.9	108.7	1.8		116.3	114.9	-1.3
166.1	166.2	0.1		149.6	149.6	0.0
201.2	201.5	0.3		180.6	183.2	2.6
232.7	232.8	0.1		212.5	212.9	0.5
241.7	242.6	0.8		258.1	258.5	0.5
324.9	327.4	2.5		341.9	345.6	3.8
346.0	348.9	2.9		363.9	365.6	1.6
425.6	424.5	-1.1		375.8	376.8	1.0
466.9	464.2	-2.7		482.5	481.7	-0.8
549.9	550.3	0.5		537.0	537.9	0.9
593.0	597.9	4.8		573.8	575.8	2.0
604.0	600.7	-3.3		604.0	600.2	-3.8

604.5	602.7	-1.8		604.4	601.7	-2.7
657.6	657.2	-0.3		651.9	652.0	0.2
678.5	675.2	-3.3		686.2	683.3	-2.9
721.2	719.2	-2.0		714.5	712.8	-1.7
738.9	739.3	0.3		749.3	750.4	1.0
751.4	756.6	5.2		752.0	755.1	3.1
752.3	759.8	7.5		754.4	760.1	5.7
781.1	780.8	-0.3		760.1	763.0	2.9
808.5	812.3	3.8		798.6	796.5	-2.1
825.4	837.1	11.8		822.7	833.1	10.4
838.3	844.7	6.5		826.3	838.0	11.8
886.8	894.9	8.1		869.4	873.1	3.7
887.7	897.8	10.1		883.9	893.4	9.5
900.7	898.7	-1.9		888.9	898.0	9.0
901.8	900.3	-1.5		902.0	900.7	-1.3
906.1	904.6	-1.5		904.6	902.5	-2.1
924.6	927.2	2.6		913.8	910.8	-3.1
939.9	936.7	-3.2		942.5	938.3	-4.3
943.4	941.4	-2.1		945.0	940.3	-4.6
986.5	987.6	1.1		981.3	983.5	2.3
1007.1	1003.9	-3.2		1003.8	1002.3	-1.5
1025.0	1020.9	-4.1		1024.8	1019.7	-5.1
1026.6	1023.0	-3.6		1025.9	1022.0	-3.8
1103.1	1098.5	-4.6		1102.5	1097.5	-4.9
1105.6	1101.8	-3.9		1105.1	1100.3	-4.8
1135.6	1138.9	3.3		1163.0	1156.3	-6.7
1170.3	1162.9	-7.5		1171.4	1163.6	-7.8
1172.0	1164.9	-7.1		1180.3	1176.2	-4.0
1205.3	1205.4	0.1		1207.7	1208.0	0.3
1228.7	1229.3	0.6		1231.0	1230.2	-0.7
1236.7	1238.7	2.1		1264.1	1264.8	0.7
1277.1	1274.6	-2.5		1273.9	1275.7	1.8
1306.5	1306.0	-0.5		1303.6	1302.4	-1.2
1310.9	1311.5	0.6		1307.0	1308.5	1.5
1337.5	1338.6	1.1		1314.6	1313.2	-1.4
1399.1	1399.8	0.8		1397.6	1396.7	-0.9
1417.2	1413.8	-3.4		1418.5	1413.0	-5.5
1419.7	1415.8	-3.9		1419.1	1415.4	-3.7
1511.5	1504.3	-7.3		1512.7	1503.9	-8.7
1518.3	1513.7	-4.7		1517.5	1514.2	-3.3
1586.5	1575.6	-10.8		1591.3	1585.1	-6.2
1595.3	1584.9	-10.4		1593.9	1589.7	-4.2
1648.3	1607.2	-41.1		1684.0	1624.1	-60.0
1685.9	1671.6	-14.3		1694.9	1678.8	-16.2

1714.3	1691.0	-23.3		1696.0	1689.1	-7.0
3154.3	3158.6	4.3		3156.1	3161.5	5.4
3178.3	3176.3	-2.1		3177.1	3178.7	1.6
3180.9	3185.1	4.2		3185.1	3183.7	-1.4
3217.5	3221.5	4.0		3194.2	3194.9	0.7
3249.0	3251.5	2.4		3248.3	3251.0	2.8
3250.2	3252.9	2.7		3250.1	3252.6	2.5
3261.0	3262.5	1.5		3260.4	3261.9	1.5
3261.6	3263.5	1.9		3261.6	3263.6	2.0
3283.5	3284.5	1.1		3284.6	3284.9	0.3
3285.8	3284.9	-0.8		3286.2	3285.2	-1.0
FAF19				FAF20		
gas	PCM	Δ		gas	PCM	Δ
24.6	24.3	-0.3		16.4	17.4	0.9
30.5	34.5	4.0		35.6	38.4	2.8
47.1	49.8	2.7		60.5	60.1	-0.5
73.4	72.5	-0.9		84.0	77.2	-6.7
90.9	92.9	2.0		97.8	97.0	-0.8
120.0	122.2	2.2		145.2	151.0	5.8
151.5	151.2	-0.3		154.7	157.3	2.6
185.6	189.4	3.8		172.6	172.7	0.1
218.5	217.1	-1.4		203.2	203.4	0.2
256.1	258.4	2.3		229.8	229.3	-0.5
344.1	348.2	4.1		306.7	308.1	1.4
360.0	361.7	1.7		368.4	367.6	-0.8
373.6	375.3	1.7		421.6	421.5	-0.1
480.3	477.5	-2.8		432.6	431.3	-1.3
537.5	539.4	1.9		550.0	550.5	0.5
571.7	575.9	4.1		603.4	601.1	-2.3
603.7	600.1	-3.5		606.7	604.3	-2.4
606.2	604.0	-2.2		622.4	625.6	3.2
651.9	652.1	0.2		663.8	662.1	-1.8
679.1	676.2	-2.9		664.6	662.5	-2.2
714.5	712.7	-1.8		740.6	742.8	2.2
744.7	748.1	3.4		745.4	745.9	0.5
752.4	755.6	3.2		751.8	758.8	6.9
757.1	761.0	3.8		766.7	769.9	3.2
760.7	765.1	4.5		794.9	794.3	-0.6
798.9	796.6	-2.3		798.2	803.1	4.8
825.9	831.1	5.2		826.5	837.8	11.3
826.6	840.7	14.1		858.6	861.5	2.9
867.4	870.7	3.3		890.9	892.6	1.7
888.5	894.9	6.4		893.9	898.5	4.6
891.3	897.9	6.6		902.1	900.6	-1.5

902.0	900.2	-1.8		902.5	902.6	0.1
904.0	901.6	-2.4		905.2	905.9	0.7
910.2	907.5	-2.7		927.0	920.9	-6.1
944.6	940.4	-4.2		956.8	954.9	-1.9
957.6	955.1	-2.5		963.7	960.7	-3.0
983.4	985.6	2.2		993.5	994.1	0.6
1005.2	1002.2	-3.0		1006.2	1007.8	1.6
1026.8	1023.8	-2.9		1023.8	1013.7	-10.1
1031.2	1026.9	-4.4		1027.3	1022.6	-4.7
1102.7	1098.0	-4.7		1113.9	1108.2	-5.7
1113.3	1110.0	-3.3		1115.3	1110.9	-4.4
1160.1	1151.9	-8.2		1154.4	1148.4	-6.1
1171.0	1163.7	-7.3		1163.4	1155.8	-7.6
1186.4	1186.5	0.1		1181.9	1174.3	-7.6
1204.9	1203.1	-1.8		1187.6	1182.9	-4.7
1211.8	1211.0	-0.8		1235.7	1237.4	1.6
1258.2	1259.9	1.7		1245.2	1243.4	-1.8
1266.6	1269.4	2.8		1263.2	1265.3	2.1
1283.1	1285.3	2.3		1289.0	1292.2	3.2
1305.8	1310.3	4.5		1297.0	1301.1	4.1
1313.8	1313.6	-0.2		1327.1	1327.2	0.0
1395.1	1394.9	-0.2		1411.7	1409.7	-2.0
1419.7	1416.3	-3.3		1419.5	1417.2	-2.3
1429.7	1427.8	-1.9		1461.0	1457.5	-3.4
1511.5	1502.8	-8.7		1509.3	1499.2	-10.1
1518.4	1513.0	-5.3		1517.5	1513.6	-3.9
1592.6	1585.1	-7.4		1599.5	1579.6	-19.9
1614.3	1608.3	-6.0		1609.6	1602.6	-7.0
1677.3	1624.5	-52.8		1624.7	1610.6	-14.1
1686.6	1676.0	-10.6		1678.2	1661.9	-16.3
1694.0	1679.8	-14.2		1692.8	1670.7	-22.1
3156.7	3158.9	2.2		3155.9	3160.3	4.5
3178.1	3177.1	-1.0		3172.6	3171.2	-1.4
3185.9	3185.0	-0.9		3185.9	3190.5	4.6
3193.1	3195.8	2.7		3203.1	3211.5	8.3
3250.8	3253.7	2.9		3251.9	3253.7	1.8
3252.5	3254.0	1.5		3251.9	3254.7	2.8
3262.1	3264.3	2.2		3264.3	3264.6	0.3
3272.7	3273.1	0.4		3278.2	3280.5	2.3
3284.6	3285.1	0.5		3286.0	3286.0	0.0
3285.3	3285.4	0.1		3303.9	3306.3	2.4
FAF21				FAF22		
gas	PCM	Δ		gas	PCM	Δ
26.6	27.8	1.1		16.9	17.9	0.9

34.3	31.5	-2.8		36.8	41.8	5.0
35.6	36.7	1.1		61.6	63.1	1.5
70.8	71.8	1.0		85.9	79.2	-6.7
86.9	87.0	0.0		94.7	92.6	-2.0
112.6	112.8	0.2		158.8	155.5	-3.3
163.6	163.7	0.1		162.4	163.3	0.9
199.0	199.7	0.6		166.4	172.1	5.7
227.2	227.6	0.4		209.5	209.7	0.2
246.8	248.0	1.2		229.4	229.5	0.0
322.4	325.2	2.8		322.1	325.9	3.8
328.1	326.7	-1.5		377.9	377.5	-0.4
421.0	421.2	0.2		422.9	423.3	0.4
463.5	459.7	-3.8		432.7	432.3	-0.4
547.6	548.2	0.6		550.7	550.9	0.2
590.9	596.7	5.8		604.0	601.0	-3.1
604.1	600.6	-3.6		606.6	604.2	-2.4
605.6	603.5	-2.1		622.8	626.6	3.8
652.4	652.2	-0.2		665.0	662.7	-2.3
673.6	670.7	-2.9		672.8	670.0	-2.8
720.0	718.4	-1.6		743.0	744.8	1.8
736.4	737.9	1.6		743.6	745.3	1.7
750.8	757.4	6.6		754.2	760.8	6.5
752.9	758.2	5.3		766.6	769.6	3.0
781.4	782.1	0.6		795.9	798.7	2.8
810.4	812.5	2.1		799.0	803.2	4.2
823.8	835.7	11.9		825.6	839.3	13.6
835.4	843.0	7.6		858.7	861.4	2.7
887.4	893.0	5.6		887.5	892.6	5.1
888.6	898.8	10.2		893.6	898.2	4.6
893.8	899.1	5.3		902.0	899.3	-2.7
902.7	900.8	-1.9		904.7	901.8	-2.9
904.6	901.6	-3.1		906.2	909.6	3.3
919.0	920.3	1.3		927.4	921.0	-6.4
941.4	937.8	-3.6		944.7	940.2	-4.6
968.9	967.5	-1.4		959.8	958.5	-1.3
986.8	988.3	1.5		994.1	993.1	-1.0
1008.5	1002.3	-6.2		1005.9	1008.4	2.5
1025.5	1022.3	-3.2		1023.9	1013.7	-10.2
1026.9	1024.2	-2.7		1026.3	1023.3	-3.1
1104.9	1101.7	-3.2		1102.5	1097.0	-5.5
1114.3	1108.1	-6.2		1114.9	1110.6	-4.3
1137.6	1141.3	3.7		1160.9	1152.1	-8.9
1156.7	1150.0	-6.7		1170.7	1161.7	-9.0
1170.2	1163.9	-6.3		1183.6	1177.3	-6.3

1191.4	1185.8	-5.6		1206.2	1206.8	0.6
1225.1	1227.0	1.9		1235.4	1237.5	2.1
1235.2	1238.0	2.9		1241.2	1238.7	-2.5
1258.6	1260.2	1.6		1276.2	1278.6	2.4
1309.6	1308.2	-1.4		1288.0	1290.2	2.2
1313.1	1313.1	0.0		1305.9	1307.6	1.7
1350.0	1354.4	4.4		1313.9	1315.8	1.8
1399.0	1400.1	1.1		1411.7	1409.7	-2.0
1418.8	1415.7	-3.0		1419.8	1415.7	-4.1
1421.2	1418.6	-2.6		1461.0	1458.3	-2.7
1508.4	1499.6	-8.7		1512.0	1503.4	-8.6
1514.5	1509.4	-5.1		1518.6	1514.8	-3.7
1586.7	1577.2	-9.5		1590.5	1575.5	-15.0
1609.2	1596.2	-13.0		1604.0	1599.5	-4.5
1643.3	1607.6	-35.7		1624.6	1603.0	-21.6
1686.1	1670.5	-15.6		1687.1	1663.5	-23.6
1710.2	1684.0	-26.2		1693.8	1678.3	-15.5
3154.7	3160.1	5.4		3155.2	3160.4	5.2
3178.7	3177.4	-1.4		3182.1	3180.5	-1.6
3180.1	3179.1	-1.0		3191.1	3198.0	6.9
3201.6	3212.4	10.7		3202.4	3212.9	10.6
3249.1	3251.9	2.8		3249.8	3253.7	3.9
3249.9	3252.3	2.4		3251.9	3254.6	2.7
3260.9	3262.8	2.0		3261.6	3264.3	2.7
3262.1	3263.2	1.1		3278.1	3280.3	2.3
3285.0	3285.0	0.1		3285.2	3285.2	0.0
3285.1	3285.5	0.3		3304.6	3306.3	1.7
FAF23				FAF24		
gas	PCM	Δ		gas	PCM	Δ
29.8	28.9	-0.9		24.3	23.7	-0.5
36.3	37.6	1.2		36.4	36.1	-0.3
42.8	41.3	-1.5		43.6	42.9	-0.7
79.0	77.6	-1.3		89.8	79.1	-10.7
90.4	90.7	0.3		117.8	117.7	-0.2
111.2	111.2	-0.1		128.5	127.5	-1.0
171.6	171.5	-0.1		174.8	172.3	-2.5
214.6	214.8	0.2		214.8	213.6	-1.2
230.5	230.9	0.4		220.4	220.9	0.6
241.4	241.9	0.6		235.6	234.7	-0.8
313.4	315.1	1.7		316.1	318.0	1.8
349.5	349.2	-0.3		389.7	386.5	-3.1
425.1	422.9	-2.2		440.1	440.3	0.2
463.3	459.5	-3.9		478.7	473.1	-5.5
550.8	551.1	0.3		593.9	598.3	4.4

596.3	599.8	3.5		605.3	602.0	-3.3
604.1	602.2	-1.9		607.5	605.8	-1.7
605.2	602.8	-2.5		641.2	640.1	-1.1
654.9	654.7	-0.2		651.7	650.7	-1.0
674.3	670.4	-3.9		655.7	654.5	-1.2
726.1	725.5	-0.6		700.5	701.2	0.8
738.9	738.9	0.1		737.5	738.2	0.6
752.3	758.3	6.0		751.9	756.5	4.6
754.3	760.9	6.6		767.0	771.0	4.0
783.0	782.7	-0.3		776.9	776.2	-0.6
809.4	812.1	2.7		790.7	791.6	1.0
829.0	839.4	10.4		834.9	841.5	6.6
840.9	845.6	4.7		857.8	858.7	0.9
889.0	896.4	7.4		865.5	864.9	-0.6
892.5	897.8	5.3		892.3	894.9	2.6
901.6	899.5	-2.1		897.3	899.2	1.9
903.0	901.6	-1.4		903.4	900.7	-2.7
905.4	905.9	0.5		905.9	903.8	-2.1
922.8	926.1	3.3		925.6	920.8	-4.8
940.0	936.3	-3.8		948.5	945.3	-3.3
954.1	953.6	-0.5		962.1	960.7	-1.4
989.8	989.9	0.1		988.2	988.4	0.2
1009.3	1002.6	-6.8		1008.3	1011.0	2.6
1026.0	1022.3	-3.7		1025.3	1015.3	-10.0
1031.9	1027.7	-4.2		1032.0	1029.4	-2.6
1103.3	1098.6	-4.7		1111.7	1107.7	-3.9
1112.3	1109.2	-3.2		1115.5	1111.7	-3.7
1149.3	1152.5	3.2		1153.8	1151.4	-2.5
1162.6	1156.3	-6.3		1161.3	1152.6	-8.7
1172.1	1164.4	-7.8		1166.1	1161.5	-4.6
1197.5	1193.3	-4.2		1184.8	1180.0	-4.8
1211.4	1211.1	-0.3		1200.2	1195.5	-4.8
1237.7	1240.2	2.4		1236.3	1235.3	-0.9
1269.4	1269.9	0.5		1243.0	1244.8	1.9
1281.0	1279.2	-1.9		1275.8	1278.0	2.1
1307.2	1305.4	-1.8		1284.6	1284.4	-0.1
1337.2	1339.1	1.9		1396.3	1397.4	1.1
1397.9	1398.7	0.8		1410.4	1408.9	-1.5
1417.7	1414.0	-3.6		1428.7	1427.2	-1.6
1430.9	1429.1	-1.8		1454.5	1450.0	-4.5
1514.5	1506.7	-7.8		1515.2	1507.8	-7.4
1517.5	1512.4	-5.1		1517.4	1513.0	-4.5
1594.3	1581.7	-12.7		1600.6	1583.2	-17.4
1607.7	1597.5	-10.2		1609.1	1600.9	-8.2

1647.1	1613.0	-34.2		1620.0	1607.5	-12.5
1672.3	1659.7	-12.6		1674.4	1663.5	-10.8
1711.8	1688.9	-23.0		1698.6	1675.3	-23.3
3154.5	3155.2	0.8		3154.8	3155.5	0.7
3180.0	3176.8	-3.2		3155.5	3159.9	4.4
3180.8	3185.6	4.8		3179.4	3175.2	-4.1
3210.6	3213.1	2.5		3195.9	3200.6	4.7
3250.6	3253.0	2.5		3252.4	3255.1	2.7
3253.4	3255.8	2.4		3252.9	3255.3	2.4
3261.8	3263.6	1.8		3272.5	3273.1	0.6
3273.3	3273.6	0.3		3278.4	3280.6	2.3
3283.8	3284.3	0.6		3284.8	3285.1	0.3
3284.8	3285.3	0.5		3303.5	3304.6	1.1
FAF25				FAF26		
gas	PCM	Δ		gas	PCM	Δ
11.0	12.4	1.4		19.0	19.3	0.3
28.8	24.1	-4.7		28.0	30.9	2.8
38.5	37.6	-0.9		30.6	32.4	1.8
54.0	53.5	-0.4		54.6	53.1	-1.4
103.3	103.6	0.3		110.5	109.1	-1.4
129.9	132.3	2.4		142.2	142.2	0.0
153.5	154.1	0.6		154.5	153.4	-1.1
199.1	194.7	-4.4		202.2	198.0	-4.3
220.1	216.2	-4.0		217.9	216.9	-1.1
246.6	249.3	2.6		248.1	248.2	0.1
330.9	335.1	4.2		329.1	329.1	-0.1
371.6	373.0	1.3		368.6	368.6	0.0
422.5	421.2	-1.4		420.6	420.0	-0.6
482.0	478.6	-3.4		479.1	476.8	-2.2
571.7	576.8	5.2		577.4	578.8	1.4
602.7	599.8	-2.9		603.7	601.4	-2.3
605.2	601.3	-3.9		604.9	601.8	-3.1
627.8	630.1	2.3		633.1	632.8	-0.3
643.4	642.1	-1.2		639.2	640.0	0.8
656.3	657.9	1.6		647.6	646.5	-1.2
720.4	717.5	-2.9		722.1	719.6	-2.5
725.3	730.0	4.7		727.0	731.0	4.0
744.1	746.6	2.5		745.0	746.2	1.2
749.4	753.9	4.5		751.5	755.6	4.1
750.2	755.4	5.2		752.8	756.2	3.4
788.1	787.6	-0.5		791.8	789.9	-1.9
823.2	833.7	10.5		828.9	837.5	8.6
827.5	836.0	8.5		831.6	838.3	6.7
855.8	853.1	-2.7		852.7	852.9	0.2

864.8	862.7	-2.1		861.1	854.8	-6.2
886.8	897.0	10.2		890.6	899.0	8.3
887.2	897.4	10.2		891.1	899.2	8.1
904.3	900.9	-3.4		904.0	900.2	-3.8
905.2	901.7	-3.5		904.8	901.7	-3.1
942.6	939.4	-3.2		945.8	942.7	-3.2
951.9	947.9	-4.0		956.7	953.4	-3.3
986.2	988.3	2.2		991.2	990.7	-0.4
989.2	990.2	1.1		991.8	990.7	-1.1
1026.6	1023.6	-2.9		1031.3	1027.4	-3.9
1029.2	1026.0	-3.2		1031.5	1027.7	-3.8
1106.2	1103.0	-3.2		1111.6	1108.3	-3.3
1112.9	1110.2	-2.7		1117.9	1114.4	-3.6
1153.5	1152.7	-0.8		1160.8	1153.0	-7.8
1162.6	1155.3	-7.3		1164.0	1155.0	-8.9
1172.2	1168.3	-3.9		1174.1	1175.1	1.0
1194.3	1188.7	-5.6		1193.7	1188.9	-4.8
1215.6	1218.1	2.5		1197.5	1193.0	-4.4
1233.5	1234.5	1.0		1229.7	1228.8	-0.9
1262.2	1265.7	3.5		1261.5	1262.4	0.9
1294.9	1300.2	5.3		1275.7	1276.6	1.0
1319.0	1321.6	2.5		1311.9	1311.1	-0.8
1396.3	1398.1	1.8		1398.8	1398.6	-0.1
1399.1	1401.0	1.9		1399.5	1399.1	-0.3
1420.8	1417.3	-3.5		1431.5	1429.0	-2.5
1431.7	1431.3	-0.4		1436.2	1433.7	-2.5
1513.5	1506.2	-7.4		1514.5	1506.2	-8.2
1517.6	1511.9	-5.7		1515.9	1510.4	-5.5
1589.0	1582.7	-6.3		1611.0	1601.0	-10.0
1613.4	1605.4	-7.9		1614.1	1610.6	-3.5
1678.5	1622.2	-56.3		1672.7	1626.9	-45.8
1683.3	1670.4	-13.0		1677.5	1666.0	-11.4
1694.4	1686.0	-8.4		1685.5	1677.1	-8.4
3152.2	3152.7	0.5		3150.3	3153.0	2.7
3152.4	3159.7	7.3		3150.3	3153.0	2.8
3173.8	3175.2	1.4		3173.3	3173.7	0.3
3180.9	3176.6	-4.3		3174.4	3174.9	0.5
3249.7	3251.9	2.2		3253.8	3255.7	2.0
3251.9	3255.2	3.2		3253.8	3255.8	2.0
3261.4	3262.7	1.3		3280.5	3280.1	-0.4
3277.9	3279.4	1.5		3280.6	3280.1	-0.5
3285.1	3284.7	-0.3		3286.5	3286.0	-0.5
3285.1	3285.7	0.6		3286.6	3286.1	-0.5
FAF27				FAF28		

gas	PCM	Δ		gas	PCM	Δ
15.8	18.5	2.7		11.1	16.4	5.2
30.2	28.8	-1.4		40.6	42.6	2.1
39.1	41.3	2.2		44.4	46.2	1.8
47.7	51.8	4.1		81.2	82.6	1.4
109.8	111.3	1.4		92.9	93.2	0.3
129.3	129.4	0.1		126.6	127.4	0.7
147.0	148.1	1.1		150.4	150.5	0.1
194.6	194.9	0.2		174.6	181.1	6.5
222.2	225.4	3.2		217.0	220.4	3.4
233.0	232.9	-0.1		236.5	237.3	0.8
326.4	331.4	5.1		334.0	338.1	4.0
389.1	390.4	1.3		372.1	372.6	0.5
448.9	449.7	0.8		393.2	397.4	4.2
465.6	461.9	-3.7		462.0	462.3	0.3
593.7	599.4	5.7		538.7	541.2	2.4
603.9	600.7	-3.1		598.2	599.0	0.9
604.6	602.4	-2.2		604.1	600.7	-3.4
636.4	639.1	2.7		605.8	605.6	-0.2
645.0	644.4	-0.6		658.5	657.5	-1.0
667.9	667.4	-0.5		681.2	677.9	-3.3
704.6	705.1	0.4		721.2	719.7	-1.6
731.8	733.8	1.9		749.6	752.1	2.4
751.5	757.0	5.5		753.9	755.0	1.1
756.7	758.5	1.7		754.0	762.6	8.6
768.1	771.1	3.0		764.4	771.6	7.1
778.5	780.7	2.2		806.2	809.2	3.0
823.0	833.9	10.9		821.6	836.7	15.1
831.4	842.4	11.0		830.4	839.4	9.0
853.7	855.6	1.9		869.2	873.6	4.4
885.8	898.8	13.0		884.6	896.1	11.6
886.3	898.8	12.5		889.4	898.2	8.8
904.4	900.0	-4.4		902.0	900.0	-2.0
904.8	900.7	-4.1		904.6	903.3	-1.3
917.8	917.3	-0.6		926.6	924.2	-2.3
941.4	937.4	-4.0		944.7	939.7	-5.0
947.3	941.9	-5.4		947.2	942.5	-4.7
989.4	991.4	2.0		986.1	994.5	8.4
992.6	998.5	5.9		1000.6	998.7	-1.9
1025.8	1022.9	-2.9		1025.8	1022.5	-3.3
1028.1	1025.2	-2.9		1026.6	1023.5	-3.1
1105.2	1101.5	-3.7		1101.6	1096.4	-5.2
1107.1	1102.7	-4.4		1107.1	1101.9	-5.2
1133.2	1136.7	3.5		1168.7	1160.1	-8.7

1170.5	1162.7	-7.8		1172.7	1163.8	-9.0
1175.2	1164.6	-10.6		1200.4	1202.0	1.7
1215.0	1213.7	-1.3		1202.3	1204.4	2.1
1228.3	1229.5	1.3		1227.0	1229.7	2.7
1236.5	1237.9	1.4		1234.9	1235.1	0.2
1239.9	1242.7	2.7		1269.5	1274.2	4.8
1312.5	1316.0	3.5		1302.6	1306.5	3.9
1325.0	1326.6	1.5		1310.3	1310.7	0.4
1395.9	1398.2	2.3		1314.9	1315.1	0.2
1417.2	1416.4	-0.8		1413.2	1415.1	1.9
1420.5	1418.6	-2.0		1419.1	1415.8	-3.3
1426.5	1421.0	-5.5		1423.7	1418.0	-5.7
1510.0	1504.5	-5.6		1511.7	1504.0	-7.7
1516.1	1514.7	-1.3		1517.1	1515.3	-1.8
1582.6	1578.8	-3.8		1589.2	1586.0	-3.2
1586.5	1580.0	-6.5		1593.6	1587.9	-5.8
1658.3	1615.3	-43.0		1673.1	1627.9	-45.2
1686.8	1671.5	-15.3		1689.2	1674.2	-15.1
1715.1	1691.9	-23.2		1712.4	1692.4	-20.0
3150.1	3158.1	8.0		3151.3	3162.9	11.5
3152.5	3160.3	7.8		3171.2	3180.1	8.9
3176.1	3175.9	-0.2		3177.5	3183.5	6.0
3177.1	3184.6	7.5		3193.4	3190.2	-3.2
3246.6	3250.5	3.9		3247.5	3250.6	3.1
3248.7	3251.9	3.2		3249.8	3252.9	3.1
3259.5	3261.6	2.1		3260.1	3261.5	1.4
3260.6	3262.8	2.2		3261.5	3263.8	2.3
3283.8	3282.8	-1.0		3284.9	3283.6	-1.3
3285.1	3284.8	-0.3		3285.0	3285.4	0.4
FAF29				FAF30		
gas	PCM	Δ		gas	PCM	Δ
30.7	25.5	-5.2		37.2	36.5	-0.7
37.1	35.7	-1.3		38.9	40.8	1.8
43.3	42.1	-1.1		50.0	51.4	1.3
89.2	88.8	-0.4		81.3	82.3	0.9
101.7	100.5	-1.1		111.9	112.4	0.5
111.4	110.7	-0.8		113.2	112.6	-0.6
153.7	154.0	0.2		151.6	153.5	1.9
186.5	186.4	-0.1		159.2	164.7	5.4
198.2	201.5	3.3		195.4	197.2	1.7
242.1	242.0	-0.1		281.0	283.2	2.1
326.1	323.8	-2.2		324.2	326.7	2.5
346.9	347.6	0.7		358.0	358.0	0.0
370.7	370.5	-0.2		358.8	362.9	4.1

413.6	412.9	-0.6		410.2	410.0	-0.2
540.5	541.5	1.1		544.6	548.4	3.7
604.1	600.6	-3.5		583.2	584.8	1.6
604.2	601.1	-3.1		604.4	601.2	-3.2
623.5	620.4	-3.1		604.4	601.3	-3.1
634.2	635.7	1.5		627.3	629.7	2.4
679.2	675.3	-3.8		683.3	678.4	-4.8
733.9	735.3	1.5		717.5	717.2	-0.3
752.6	760.3	7.7		753.2	759.5	6.3
754.8	760.4	5.6		753.3	759.5	6.2
760.9	762.2	1.4		774.0	773.8	-0.2
799.7	798.4	-1.3		779.5	780.7	1.2
818.6	831.2	12.6		819.0	831.6	12.6
826.4	839.3	12.9		824.5	837.3	12.8
871.7	873.2	1.5		878.9	877.6	-1.3
888.5	897.4	8.9		886.9	894.0	7.0
891.4	898.2	6.8		890.7	897.8	7.0
901.4	900.2	-1.2		898.3	898.2	-0.1
902.0	901.9	-0.2		900.7	901.1	0.4
909.2	909.3	0.2		902.8	902.5	-0.3
921.8	927.2	5.4		903.0	907.3	4.3
941.0	938.0	-3.0		944.2	939.2	-5.0
948.6	946.1	-2.5		946.4	941.9	-4.5
996.2	987.5	-8.7		1001.5	998.5	-2.9
1009.6	1004.8	-4.8		1007.6	1007.4	-0.2
1025.8	1021.3	-4.5		1025.1	1020.6	-4.5
1026.4	1022.8	-3.6		1025.8	1021.1	-4.7
1101.8	1095.6	-6.2		1101.1	1095.4	-5.8
1105.1	1100.8	-4.3		1104.8	1100.6	-4.2
1168.7	1160.8	-7.9		1170.7	1162.3	-8.4
1171.7	1164.0	-7.7		1173.0	1165.1	-8.0
1189.9	1190.7	0.8		1204.5	1205.2	0.7
1210.1	1210.1	0.1		1213.0	1212.9	-0.1
1231.4	1232.3	0.9		1264.9	1267.0	2.1
1273.6	1274.7	1.0		1276.0	1277.9	1.9
1278.8	1277.3	-1.5		1292.4	1289.9	-2.6
1301.4	1298.9	-2.5		1300.0	1302.3	2.3
1306.4	1308.0	1.6		1302.6	1304.7	2.1
1314.4	1311.8	-2.5		1313.0	1311.6	-1.3
1357.8	1356.7	-1.1		1314.2	1317.5	3.3
1417.5	1413.8	-3.7		1418.5	1414.0	-4.5
1420.1	1416.4	-3.7		1418.7	1414.6	-4.1
1511.6	1502.6	-9.0		1513.0	1503.4	-9.6
1518.1	1512.8	-5.2		1515.7	1510.6	-5.1

1592.3	1575.7	-16.6		1593.3	1581.8	-11.6
1596.3	1589.2	-7.1		1594.1	1589.4	-4.7
1649.2	1609.1	-40.1		1677.8	1615.9	-61.9
1686.8	1669.2	-17.6		1684.2	1669.4	-14.8
1709.8	1688.2	-21.6		1692.2	1682.5	-9.7
3180.3	3182.5	2.1		3180.5	3179.7	-0.9
3184.7	3183.6	-1.1		3184.6	3181.3	-3.3
3194.2	3196.2	2.0		3190.5	3193.5	3.0
3211.8	3217.0	5.2		3192.3	3198.2	5.9
3250.0	3252.8	2.9		3249.3	3252.4	3.2
3250.8	3252.9	2.1		3249.3	3252.5	3.2
3261.7	3263.5	1.8		3261.1	3263.2	2.2
3262.0	3263.8	1.8		3261.1	3263.3	2.2
3283.8	3284.5	0.7		3285.2	3285.2	0.0
3285.1	3285.4	0.3		3285.2	3285.2	0.0

6. Vertical excitation energies for the FAF species

Table S13. Vertical excitation energies (in eV) of the lowest electronic states (S_1, T_1 and T_2) at the B3/junDZ(geom)//MRCI-F12+Q/aug-cc-pVDZ level for the 30 structures of FAF studied. These energies are given with respect to the S_0 energy.

	**FAF1**	**FAF2**	**FAF3**	**FAF4**	**FAF5**	**FAF6**	**FAF7**	**FAF8**	**FAF9**	**FAF10**	**FAF11**	**FAF12**
S_0	0.00	0.00	0.00	0.00	0.00	0.00	0.00	0.00	0.00	0.00	0.00	0.00
S_1	4.41	4.79	4.36	4.06	4.40	4.43	4.40	4.22	4.18	3.94	4.54	4.38
T_1	2.61	3.23	2.79	2.85	3.10	2.98	2.61	3.01	2.65	2.83	3.32	3.21
T_2	5.18	3.22	2.88	3.04	3.16	3.11	5.17	3.14	5.04	3.18	3.42	3.21
	**FAF13**	**FAF14**	**FAF15**	**FAF16**	**FAF17**	**FAF18**	**FAF19**	**FAF20**	**FAF21**	**FAF22**	**FAF23**	**FAF24**
S_0	0.00	0.00	0.00	0.00	0.00	0.00	0.00	0.00	0.00	0.00	0.00	0.00
S_1	4.57	4.77	4.39	4.30	4.58	5.66	4.24	4.24	4.35	4.27	4.26	4.20
T_1	3.29	2.96	3.26	3.20	3.10	3.02	3.44	3.04	2.90	3.13	3.31	3.15
T_2	3.52	3.10	3.39	3.50	3.30	4.63	3.69	3.15	3.39	3.25	3.36	3.33
	**FAF25**	**FAF26**	**FAF27**	**FAF28**	**FAF29**[a]	**FAF30**						
S_0	0.00	0.00	0.00	0.00	0.00	0.00						
S_1	*4.15*	4.10	5.19	5.43	4.88	5.52						
T_1	*3.08*	3.25	2.71	2.88	3.92	2.98						
T_2	*3.25*	3.37	4.45	4.58	4.05	3.15						

[a] For FAF29, values are computed at the CASSCF/aug-cc-pVDZ level of theory.

7. Configurations and Frontiers orbital for the thirty FAF species at the CASSCF(8,8)/aug-cc-pVDZ level

Table S14. Weights of the leading configurations in the SA-CASSCF(8,8)/aug-cc-pVDZ wavefunctions of the lowest singlet and triplet excited states of the FAF conformers.

	FAF1	FAF2	FAF3	FAF4	FAF5	FAF6	FAF7	FAF8	FAF9	FAF10
S_0	0.94	0.80	0.81	0.95	0.73	0.83	0.94	0.79	0.93	0.97
S_1	0.95	0.79	0.80	0.95	0.63	0.82	0.96	0.77	0.94	0.97
T_1	0.73	0.46	0.40	0.85	0.83	0.35	0.73	0.55	0.77	0.76
T_2	0.72	0.43	0.36	0.86	0.82	0.31	0.73	0.46	0.77	0.76
	**FAF11**	**FAF12**	**FAF13**	**FAF14**	**FAF15**	**FAF16**	**FAF17**	**FAF18**	**FAF19**	**FAF20**
S_0	0.95	0.97	0.96	0.79	0.95	0.79	0.81	0.80	0.88	0.94
S_1	0.95	0.97	0.96	0.40	0.96	0.33	0.80	0.19	0.86	0.95
T_1	0.60	0.88	0.65	0.75	0.98	0.77	0.27	0.75	0.26	0.80
T_2	0.60	0.88	0.65	0.40	0.98	0.44	0.24	0.41	0.22	0.81
	**FAF21**	**FAF22**	**FAF23**	**FAF24**	**FAF25**	**FAF26**	**FAF27**	**FAF28**	**FAF29**	**FAF30**
S_0	0.81	0.94	0.85	0.88	0.79	0.85	0.79	0.83	0.96	0.88
S_1	0.80	0.95	0.84	0.87	0.30	0.85	0.23	0.83	0.97	0.86
T_1	0.29	0.95	0.30	0.37	0.75	0.52	0.76	0.27	0.90	0.26
T_2	0.24	0.95	0.25	0.28	0.42	0.42	0.44	0.26	0.90	0.31

The following figure reports the HOMO and LUMO molecular orbitals, calculated at the CASSCF/aug-cc-pVDZ level, for the singlet ground state of the 30 FAF species considered. Frontier orbitals were identified by inspection of the active orbital occupation numbers. Isodensity surfaces are shown at 0.02 a.u.

**FAF1**		**FAF2**	
HOMO	LUMO	HOMO	LUMO
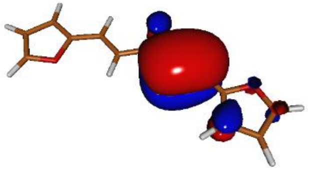	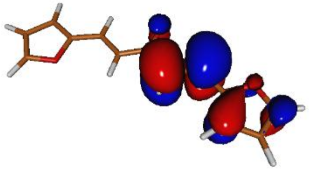	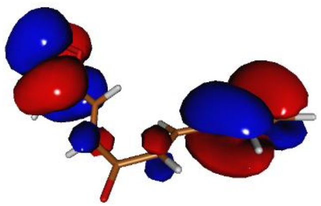	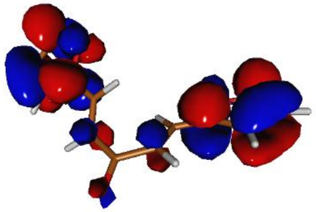
**FAF3**		**FAF4**	
HOMO	LUMO	HOMO	LUMO
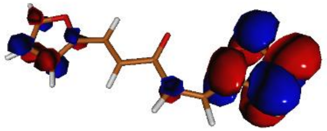	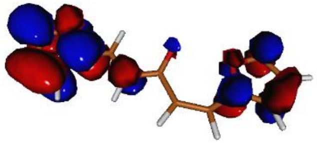	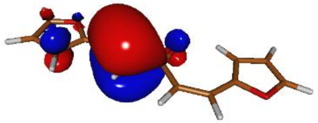	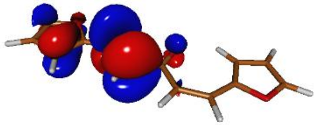
**FAF5**		**FAF6**	
HOMO	LUMO	HOMO	LUMO
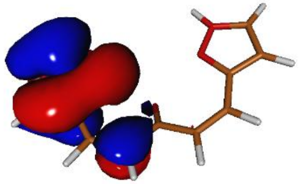	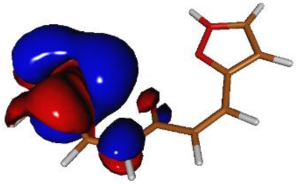	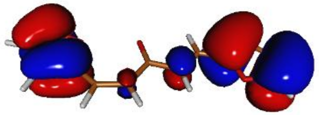	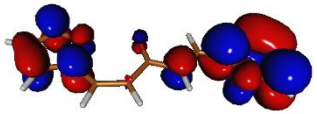

FAF7		FAF8	
HOMO	LUMO	HOMO	LUMO
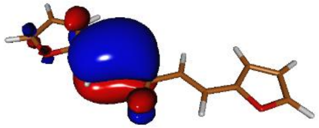	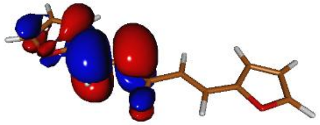	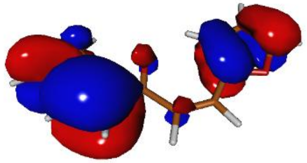	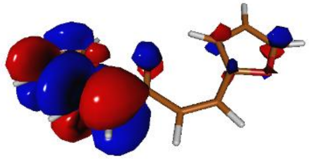
**FAF9**		**FAF10**	
HOMO	LUMO	HOMO	LUMO
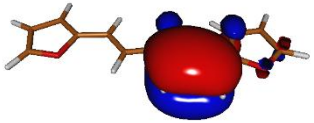	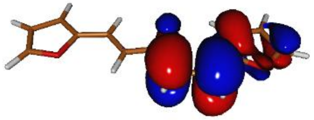	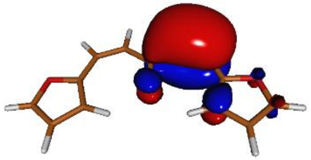	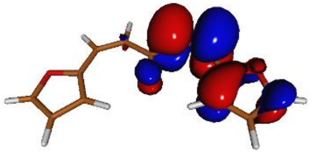
**FAF11**		**FAF12**	
HOMO	LUMO	HOMO	LUMO
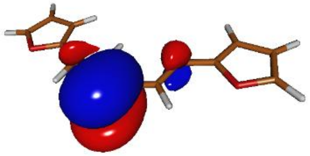	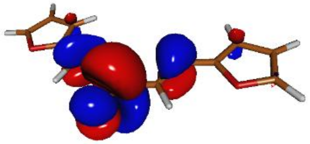	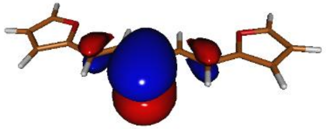	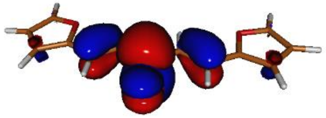

**FAF13**		**FAF14**	
HOMO	LUMO	HOMO	LUMO
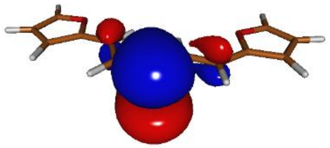	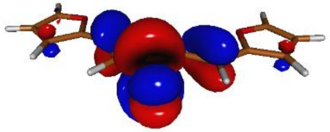	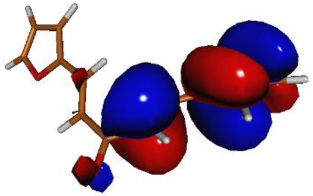	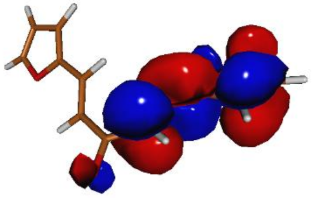
**FAF15**		**FAF16**	
HOMO	LUMO	HOMO	LUMO
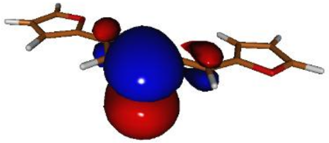	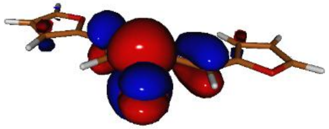	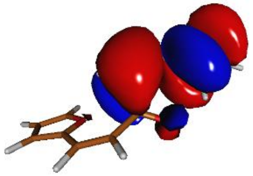	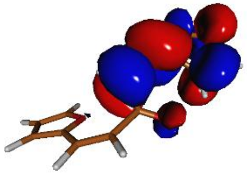
**FAF17**		**FAF18**	
HOMO	LUMO	HOMO	LUMO
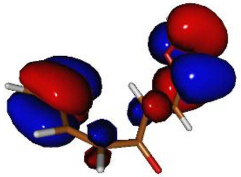	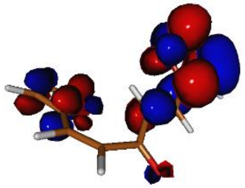	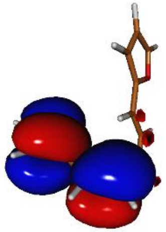	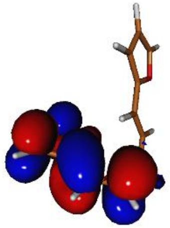

<table>
<tr><th colspan="2">FAF19</th><th colspan="2">FAF20</th></tr>
<tr><td>HOMO
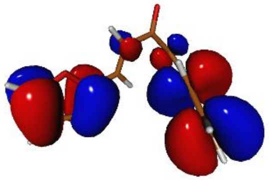</td><td>LUMO
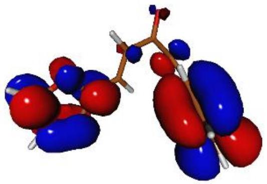</td><td>HOMO
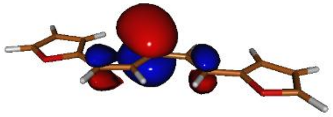</td><td>LUMO
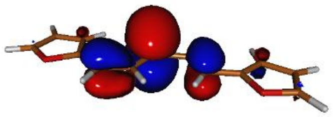</td></tr>
<tr><th colspan="2">FAF21</th><th colspan="2">FAF22</th></tr>
<tr><td>HOMO
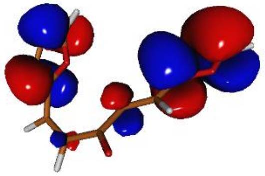</td><td>LUMO
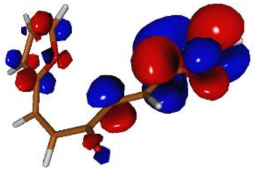</td><td>HOMO
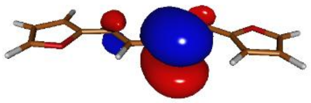</td><td>LUMO
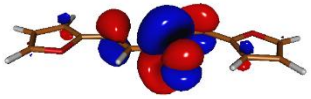</td></tr>
<tr><th colspan="2">FAF23</th><th colspan="2">FAF24</th></tr>
<tr><td>HOMO
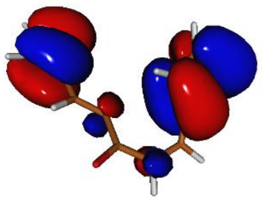</td><td>LUMO
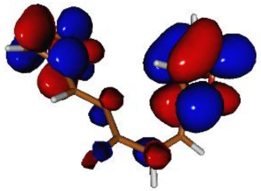</td><td>HOMO
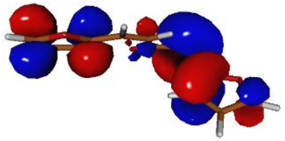</td><td>LUMO
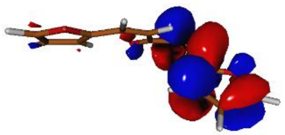</td></tr>
<tr><th colspan="2">FAF25</th><th colspan="2">FAF26</th></tr>
<tr><td>HOMO
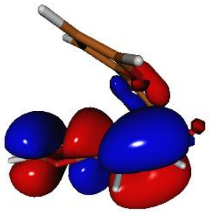</td><td>LUMO
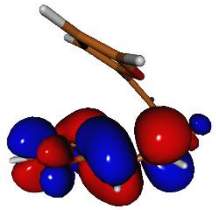</td><td>HOMO
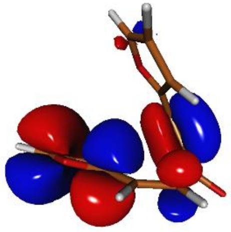</td><td>LUMO
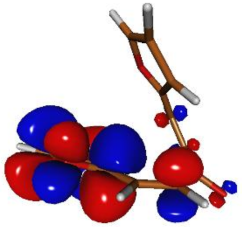</td></tr>
</table>

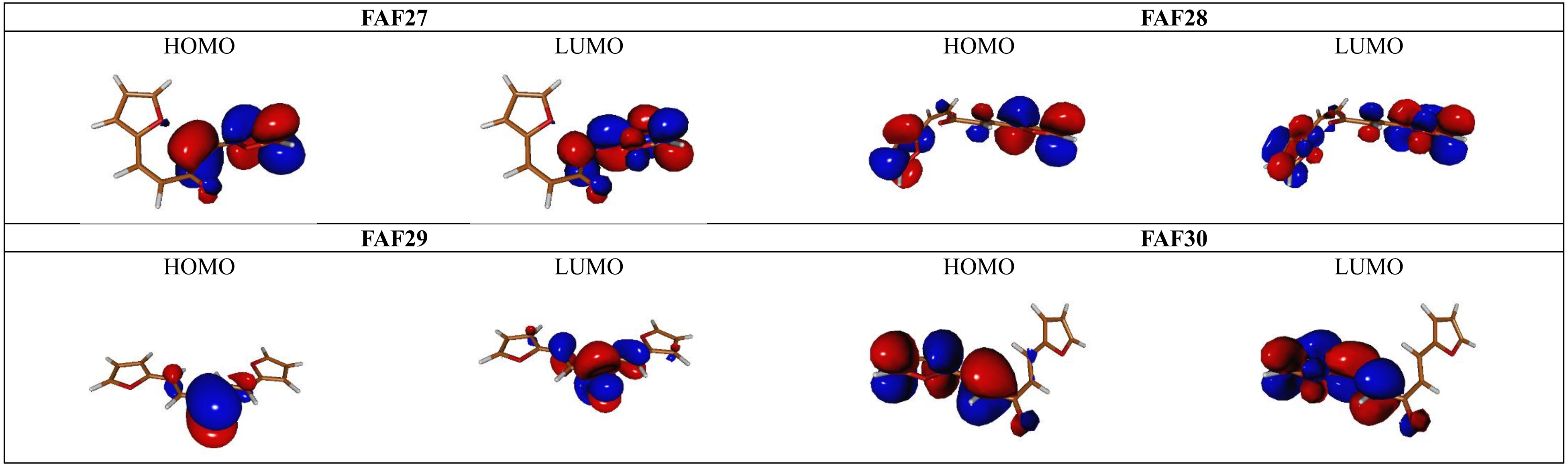

Figure S3. HOMO and LUMO molecular orbitals, calculated at the CASSCF/aug-cc-pVDZ level, for the singlet ground state of the 30 FAF species considered. Frontier orbitals were identified by inspection of the active orbital occupation numbers. Isodensity surfaces are shown at 0.02 a.u.

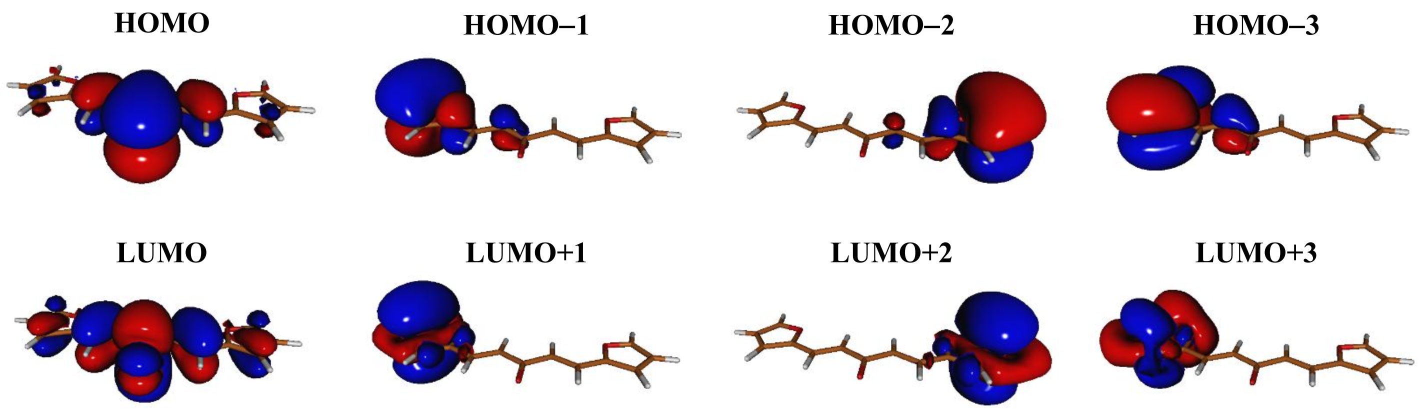

Figure S4. Outermost molecular orbitals of FAF12 as computed at the (8,8) CASSCF/aug-cc-pVDZ level for the singlet ground state. Isodensity surfaces are displayed at 0.01 a.u.
